# High Rate Capability and Cycling Stability in Multi‐Domain Nanocomposite LiNi_1–_ *_x_* Ti_3_ *_x_*_/4_O_2_ Positive Electrodes

**DOI:** 10.1002/adma.202417899

**Published:** 2025-07-22

**Authors:** Jungwoo Lim, Manel Sonni, Luke M. Daniels, Mounib Bahri, Marco Zanella, Ruiyong Chen, Zhao Li, Alex R. Neale, Hongjun Niu, Nigel D. Browning, Matthew S. Dyer, John B. Claridge, Laurence J. Hardwick, Matthew J. Rosseinsky

**Affiliations:** ^1^ Department of Chemistry University of Liverpool Crown Street Liverpool L69 7ZD UK; ^2^ Stephenson Institute for Renewable Energy Department of Chemistry University of Liverpool Liverpool L69 7ZF UK; ^3^ The Faraday Institution Didcot OX11 0RA UK; ^4^ Albert Crewe Centre University of Liverpool Research Technology Building, Elisabeth Street, Pembroke Place Liverpool L69 3GE UK; ^5^ School of Engineering Department of Mechanical Materials and Aerospace Engineering University of Liverpool Liverpool L69 3GH UK; ^6^ Leverhulme Research Centre for Functional Materials Design Materials Innovation Factory Oxford Street Liverpool L7 3NY UK

**Keywords:** high rate, Li ion batteries, nanocomposite electrode, Ni‐rich positive electrode material, rock salt

## Abstract

LiNiO_2_ positive electrode materials for lithium‐ion batteries have experienced a revival of interest due to increasing technological energy demands. Herein a specific Ti^4+^ substitution is targeted into LiNiO_2_ to access new compositions by synthesizing the LiNi_1–_ *_x_* Ti_3_ *_x_*_/4_O_2_ solid solution with the aim of retaining Ni^3+^. Compositions in the range 0.025 ≤ *x* ≤ 0.2 form nanocomposites of compositionally homogeneous ordered *R* $\bar{3}$ *m* and disordered *Fm* $\bar{3}$ *m* rock salt domains as observed via X‐ray and neutron diffraction, and STEM. The disordered rock salt domains stabilize the ordered structure to provide excellent structural reversibility via the formation of coherent interfaces during cycling and enable deep delithiation using a constant voltage charging step without structural degradation. The detrimental structural phase transitions associated with the poor cyclability of LiNiO_2_ are suppressed to yield a low strain positive electrode material with high capacity retention that offers high‐rate capability even under increased cell electrode mass loadings. The composition *x* = 0.075 (LiNi_0.925_Ti_0.05625_O_2_) affords a 93% capacity retention after 100 cycles (100 mA g^−1^) and demonstrates high reversible capacities of 125 mAh g^−1^ even under rates of 3200 mA g^−1^. LiNi_0.925_Ti_0.05625_O_2_ exhibits exceptional performance at electrode mass loadings (13.6 mg cm^−2^) comparable to those required for commercial cell applications.

## Graphical abstract

By targeting nominally non‐stoichiometric LiNi_1–_ *_x_* Ti_3_ *_x_*_/4_O_2_ compositions, a multi‐domain nanocomposite rock salt positive electrode material with exceptional cycling stability and excellent rate performance is obtained. This enhanced performance particularly under high oxidative potential protocols and cell electrode mass loadings applicable to commercial cells represents a significant advance for LiNiO_2_‐based positive electrode materials.

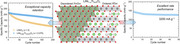

## 1 Introduction

**Figure 1:**
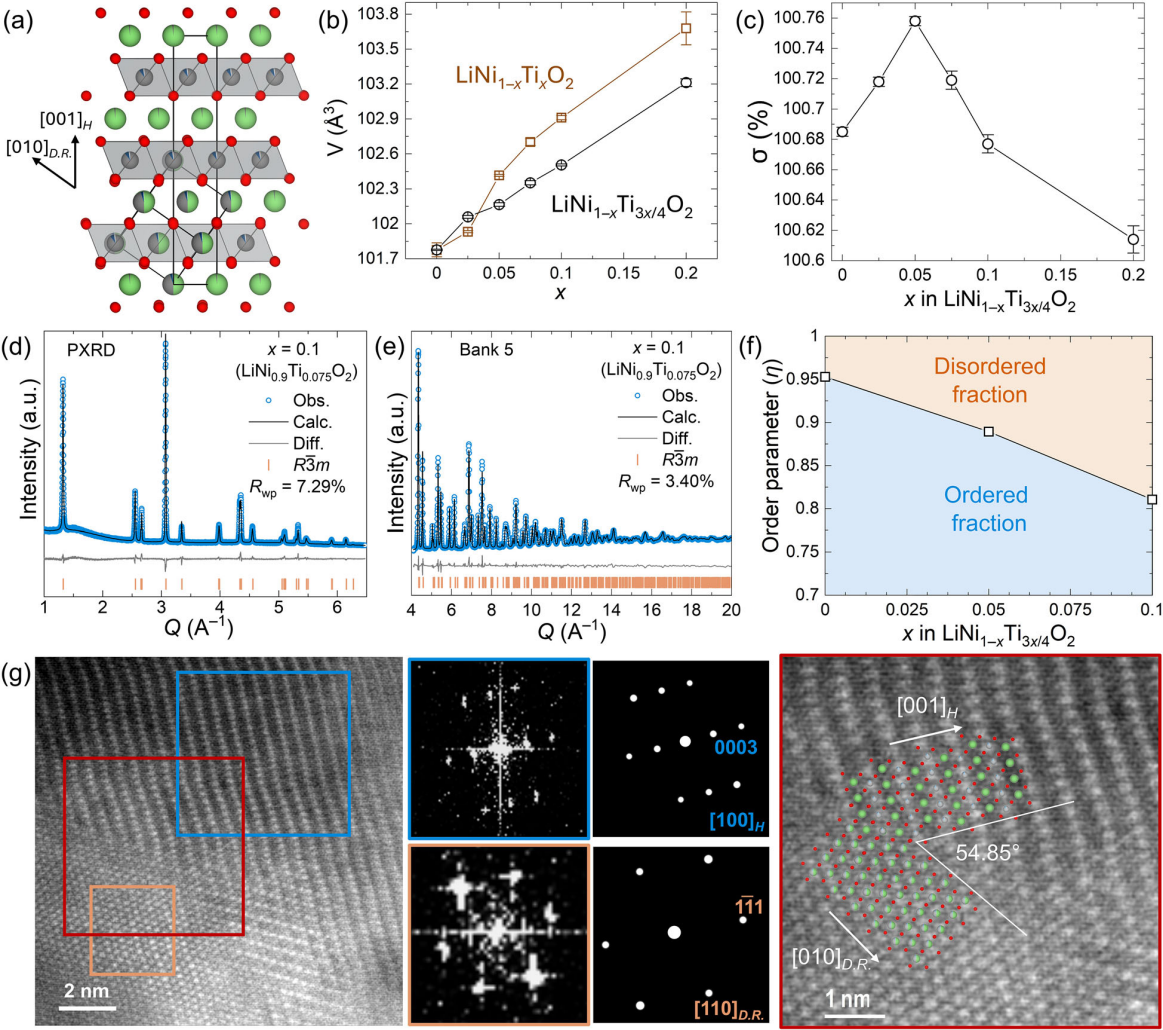
Structural Characterisation. a) The ordered *R* $\bar{3}$ *m* crystal structure of *x* = 0.1 (LiNi_0.9_Ti_0.075_O_2_) in LiNi_1–_ *_x_* Ti_3_ *_x_*_/4_O_2_, with Li (green) and transition metal sites (Ni and Ti, grey) layered along (111) direction of the disordered *Fm* $\bar{3}$ *m* unit cell which is shown inset. Trends in b) hexagonal unit cell volume (V) for LiNi_1–_ *_x_* Ti_3_ *_x_*_/4_O_2_ and LiNi_1–_ *_x_* Ti*_x_* O_2_ (0 ≤ *x* ≤ 0.2) and c) hexagonal distortion parameter (σ) (error bars are multiplied by 3) for LiNi_1–_ *_x_* Ti_3_ *_x_*_/4_O_2_ (0 < *x* < 0.2). Combined Rietveld refinement at 300 K of *x* = 0.1 (LiNi_0.9_Ti_0.075_O_2_) against d) PXRD and e) time‐of‐flight neutron powder diffraction data (Bank 5 of NOMAD). Observed (blue circles), calculated (black line), and difference (grey line) traces with Bragg reflections for *R* $\bar{3}$ *m* (orange tick marks) are shown. The combined refinement including PXRD data and NPD data across four detector banks has an R_wp_ of 3.2267%, R_exp_ of 0.4%, and GoF of 7.94 (Figure S13 and Table S8, Supporting Information). f) The chemical order parameter (*η*) extracted from Rietveld refinements for *x* = 0, 0.05 and 0.1 decreases with increasing *x*, indicating a concomitant increase in the fraction of disordered *Fm* $\bar{3}$ *m* domains. g) HAADF‐STEM image of an interfacial region observed within *x* = 0.075 (LiNi_0.925_Ti_0.05625_O_2_) showing co‐existence of the ordered *R* $\bar{3}$ *m* (blue) and disordered *Fm* $\bar{3}$ *m* rock salt (orange) domains oriented along [100]*_H_* and [110]*_DR_*_._ respectively compared with the simulated patterns (right). A schematic depiction of the coherent interface between the ordered and disordered domains is overlaid on an enlarged region of the HAADF‐STEM image (red square).

Lithium‐ion batteries remain the state‐of‐the‐art technology for an ever‐expanding variety of energy storage applications. Recent efforts have focused on maximizing the reversible capacity from positive electrode materials to improve overall lithium‐ion cell performance in markets such as the automotive industry. Ni‐rich compounds (e.g., LiNi*_x_* Co*_y_* Mn_1–_ *_x_*_–_ *_y_* O_2_, or LiNi*_x_* Co*_y_* Al_1–_ *_x_*_–_ *_y_* O_2_) are widely used as positive electrode materials, and are based on the compositional and structural chemistry of the extensively‐studied LiNiO_2_.^[^ ^1^ ^]^ LiNiO_2_ is isostructural with LiCoO_2_ but contains a strategically available transition metal element, and exhibits good initial performance with high Li^+^ ion mobility and a theoretical capacity of 275 mA g^−1^ (based on full de‐lithiation).^[^ ^2^ ^]^ The *R* $\bar{3}$ *m* layered structure of LiNiO_2_ arises from the size‐driven ordering of Li^+^ and Ni^3+^ cations into alternate rock salt (111) planes within a cubic close‐packed array of oxide anions (**Figure** **1**a), analogous to the *α*‐NaFeO_2_ structure.^[^ ^3^ ^]^ LiNiO_2_ exhibits a number of drawbacks that limit its use in commercial devices. It is well‐known that the synthesis of perfectly stoichiometric LiNiO_2_ is highly challenging with many studies confirming that the material commonly displays a slight deficiency in Li^+^ even under well‐controlled conditions, with the excess Ni present in the form of Ni^2+^.^[^ ^1^, ^4^, ^5^ ^]^ The ionic radius of Ni^2+^(*r* = 0.69 Å) is closer in size to that of Li^+^ (*r* = 0.76 Å) than Ni^3+^ (low spin *r* = 0.56 Å),^[^ ^6^ ^]^ which consequently results in unavoidable Li/Ni mixing within the layers yielding a disordered cubic *Fm* $\bar{3}$ *m* rock salt structure (Figure 1a) and a significant reduction in cycling stability. Furthermore, LiNiO_2_ undergoes irreversible first‐order structural phase transitions and large volume changes upon cycling that induce fast capacity fade.^[^ ^1^, ^2^ ^]^

Various routes have been explored to improve the performance of LiNiO_2_‐based positive electrode materials, which include the use of alternative electrolytes,^[^ ^7^ ^]^ or the optimization of particle morphology and surface design.^[^ ^8^ ^]^ Two specific materials modification routes under active exploration include the introduction of redox‐inactive cations,^[^ ^9^, ^10^, ^11^, ^12^, ^13^, ^14^, ^15^, ^16^, ^17^, ^18^, ^19^ ^]^ or the control of microstructure through the incorporation of nanodomains.^[^ ^20^, ^21^ ^]^ Substitution of Ni^3+^ for high‐valence redox‐inactive cations can be used to increase the Li content in rock salt materials,^[^ ^22^ ^]^ to activate the anionic‐redox of oxygen yielding higher capacities,^[^ ^23^, ^24^, ^25^, ^26^, ^27^ ^]^ or to stabilize the structures of Ni‐rich compounds during cycling subsequently enhancing the capacity retention.^[^ ^11^, ^28^, ^29^, ^30^, ^31^ ^]^ The direct substitution of Ti for Ni into LiNiO_2_ in the LiNi_1–_ *_x_* Ti*_x_* O_2_ (0 ≤ *x* ≤ 0.4) phases has been widely explored (Figure S1 and Table S1, Supporting Information); all of the studies report the synthesis of single‐phase ordered *R* $\bar{3}$ *m* materials where Ti^4+^ is present in the material bulk resulting in the reduction of Ni^3+^ to Ni^2+^.^[^ ^32^, ^33^ ^]^ Several of these studies demonstrate that initial capacity decreases with increasing *x*, however capacity retention is improved with increasing *x* by suppression of the phase transitions upon deintercalation.^[^ ^32^, ^33^, ^34^, ^35^, ^36^, ^37^ ^]^ Retention of the ordered *R* $\bar{3}$ *m* structure is confirmed via ex situ measurements of the materials post‐cycling in a small number of studies, but with significant volume changes in all cases.^[^ ^32^, ^34^ ^]^ Improvements in positive electrode material performance are also possible via extrinsic effects in which multiple domain structures are introduced to yield so‐called composite electrode materials. The formation of disordered rock salt structures as surface layers on bulk particles of ordered rock salt is known to enhance rate capability and capacity retention in Li_0.95_Ni_0.95_Mn_0.05_O_2_,^[^ ^38^ ^]^ LiNi_0.62_Co_0.14_Mn_0.24_O_2_,^[^ ^39^ ^]^ and Ti doped LiNi_0.8_Co_0.2_O_2_.^[^ ^40^ ^]^ Various combinations of intergrowth phases have been observed in Ni‐rich rock salt oxides which include interfaces between separate ordered domains in LiNi_0.98_W_0.02_O_2_,^[^ ^11^ ^]^ or ordered and disordered domains in Li‐deficient materials such as Li_1–_ *_m_*_(_Ni_0.94_Al_0.06_)_1+_ *_m_* O_2_,^[^ ^38^, ^41^ ^]^ and Li‐rich compounds like Li_1.2_Ni_0.4_Ru_0.4_O_2_.^[^ ^42^, ^43^ ^]^ Surface segregation of W has been observed through W^6+^ substitution into LiNiO_2_,^[^ ^44^ ^]^ or in the case of Mo^6+^ in Li_1.09_Ni_0.85_Mo_0.06_O_2_, segregation into distinct ordered LiNiO_2_‐rich domains and disordered Li_4_MoO_5_‐rich domains was observed.^[^ ^45^ ^]^ Despite this compositional heterogeneity, synergy is achieved where the disordered Li_4_MoO_5_‐rich domains epitaxially stabilize the LiNiO_2_‐rich domains during cycling, enhancing the capacity retention. Such effects significantly reduce the lattice parameter change and structural evolution during cycling which is the origin of the enhanced cycling stabilities of these low‐strain compounds.^[^ ^42^, ^43^, ^45^ ^]^

We explore an alternative compositional route to the introduction of Ti^4+^ into LiNiO_2_ via the nominally vacancy‐containing LiNi_1–_ *_x_* Ti_3_ *_x_*_/4_O_2_ solid solution, aiming to intentionally retain Ni^3+^, thus avoiding the reduction in cycling stability associated with the formation of Ni^2+^. This substitution generates intergrowths of ordered *R* $\bar{3}$ *m* and disordered *Fm* $\bar{3}$ *m* rock salt nanoscale domains in a Ni‐rich positive electrode material. The selection of Ti^4+^ arises from its d^0^ electronic configuration and comparable size to Ni^3+^ (Ti^4+^: 0.605 Å, Ni^3+^: 0.56 Å) which enables a homogeneous distribution of both cations throughout the bulk and precise control of cation disorder. The compositional homogeneity achieved across the ordered and disordered rock salt domains in a nanocomposite electrode blends the beneficial characteristics of both structures, the high rate of the former alongside the stability of the latter and yields one of the highest observed capacity retentions (93% over 100 cycles) in LiNiO_2_‐type positive electrode materials with very low strain. This enhanced stability enables cycling of the materials at high current densities (3200 mA g^−1^) and with cathode loadings (13.6 mg cm^−2^) that are comparable to commercial devices. This outperformance in compositions of LiNi_1–_ *_x_* Ti_3_ *_x_*_/4_O_2_ is related to the crystal and domain structures through detailed ex situ characterization.

## 2 Results and Discussion

### 2.1 . Synthesis and Structural Characterization

Solid state synthesis of LiNi_1–_ *_x_* Ti_3_ *_x_*_/4_O_2_ was explored in the range of 0 ≤ *x* ≤ 0.3 by ball milling stoichiometric amounts of dried LiOH (Figure S2, Supporting Information), Ni(OH)_2_ and TiO_2_ precursor mixtures, followed by heating at 700 °C for 20 h under 100 mL min^−1^ flowing O_2_ atmosphere. The reflections observed in Synchrotron X‐ray powder diffraction (SPXRD) data (Figure S3, Supporting Information) for *x* = 0 correspond to the *R* $\bar{3}$ *m* cation ordered structure of LiNiO_2_. As *x* increases in LiNi_1–_ *_x_* Ti_3_ *_x_*_/4_O_2_, substantial peak broadening is observed most notably for the (003)*_H_* reflection at *Q* = 1.3 Å^−1^ (corresponding to the (003) Bragg reflection of the LiNiO_2_ *R* $\bar{3}$ *m* structure in the hexagonal setting). In addition, a significant change in relative peak intensities is observed between the (003)*_H_* and (104)*_H_* reflections at *Q* = 1.3 Å^−1^ and 3.1 Å^−1^, respectively (Figure S3, Supporting Information).

The experimental compositions, measured by Inductively coupled plasma mass spectrometry (ICP‐MS) and Transmission Electron Microscopy Energy‐dispersive X‐ray spectroscopy (TEM‐EDX) (Table S3, Supporting Information), confirm the incorporation of Ti and overall reduction in transition metal content (Ti + Ni). The LiNi_1–_ *_x_* Ti_3_ *_x_*_/4_O_2_ compositions nominally contain vacancies, however, it is not possible to unambiguously confirm the presence of vacancies at the expected compositions via ICP‐MS, particularly at the small vacancy concentrations targeted, in the materials arising from synthesis under the conditions used. Scanning Transmission Electron Microscopy Energy‐dispersive X‐ray spectroscopy (STEM‐EDX) elemental mapping confirms the homogeneous distribution of both Ti and Ni throughout the bulk of particles from *x* = 0.075 (Figure S7, Supporting Information). Based on this, a multidomain hexagonal *R* $\bar{3}$ *m* structural model was refined against the experimental data for LiNi_1–_ *_x_* Ti_3_ *_x_*_/4_O_2_ (0 ≤ *x* ≤ 0.2) which is designed to handle multiple domain types that share a common unit cell volume, thus minimizing the overall number of refined structural parameters. Such a model was used previously to describe the long‐range structure of the Li*_x_* Ni_2–_ *_x_* O_2_ (0 ≤ *x* ≤ 1) solid solution,^[^ ^46^, ^47^ ^]^ but it did not account for the observed short‐range order. Here, we utilize the improved multidomain hexagonal *R* $\bar{3}$ *m* structural model of Barton et al.,^[^ ^48^ ^]^ to model both short‐ and long‐range order accounting for the entire diffraction pattern. A key benefit of this is that it provides an accurate representation of the extent of disorder across domains of different size. This model uses one set of unit cell parameters and separate peak profiles to describe the reflections that arise from cubic *Fm* $\bar{3}$ *m* cation disordered and cation‐ordered *R* $\bar{3}$ *m* rock salt domains (Figure S8, Supporting Information). Evaluation of separate *hkl*_even_ and *hkl*_odd_ reflection peak profiles provides insight into the coherence lengths for the disordered *Fm* $\bar{3}$ *m* and ordered *R* $\bar{3}$ *m* domains respectively, defined by the volume‐weighted column heights for the disordered (*L*_cub_) and ordered (*L*_hex_) domains.^[^ ^49^ ^]^ The disorder associated with the *Fm* $\bar{3}$ *m* rock salt structure (in which the two cation sites are equivalent) is incorporated into the *R* $\bar{3}$ *m* symmetry thus impacting both sites within this multidomain hexagonal structural model. The occupancies of the oxygen positions were refined and found to converge to unity (e.g., 0.999(1) for LiNi_0.95_Ti_0.0375_O_2_) indicating that no oxygen vacancies were present. As such, the oxygen site occupancies were fixed to unity. The model fixes the compositions of both domain types to be identical, consistent with the homogeneous Ti and Ni distribution observed via STEM‐EDX, and the global compositions of each material in LiNi_1–_ *_x_* Ti_3_ *_x_*_/4_O_2_ were restrained to the compositions measured by ICP‐MS (Table S3, Supporting Information) via a penalty function (Tables S7 and S8, Supporting Information). Restraining the models to the nominal compositions defined by *x* = 0.05 and 0.1 in LiNi_1–_ *_x_* Ti_3_ *_x_*_/4_O_2_ yields refinements which are indistinguishable from those that utilize the information from ICP‐MS measurement (e.g., for *x* = 0.05 the *R*_wp_ for the nominal composition‐restrained refinement is 3.2994%, and for the ICP composition‐restrained refinement it is 3.2996%).

The parameters refined in this multidomain structural model were: the hexagonal *a* lattice parameter and hexagonal distortion parameter (σ) from which the hexagonal *c* lattice parameter can be calculated (*c* = *a* $\sigma \sqrt{24}$); the fractional *z* coordinate of the oxygen position; and the chemical order parameter (*η*). The chemical order parameter provides direct insight into the degree of Li/M site mixing (M = Ni, Ti) and is defined as the difference in Li content between the two distinct cation (Li and Ni) layers (*η* = |*occ*_Li_ – *occ*_Ni_|). For example, in perfectly ordered *R* $\bar{3}$ *m* LiNiO_2_, σ > 1 and *η* = 1, whereas in a fully disordered cubic *Fm* $\bar{3}$ *m* rock salt, σ = 1 and *η* = 0. Previously, the refinement of the average structures of stoichiometric LiNi_1–_ *_x_* Ti*_x_* O_2_ (*x* ≤ 0.2) materials did not account for multiple domain types (Table S1, Supporting Information).^[^ ^32^, ^36^, ^50^ ^]^ The multidomain *R* $\bar{3}$ *m* model accurately describes the data for the nominal compositions of LiNi_1–_ *_x_* Ti_3_ *_x_*_/4_O_2_ in the range 0 ≤ *x* ≤ 0.2. For *x* = 0.3 (LiNi_0.7_Ti_0.225_O_2_), peak splitting is observed (Figures S3,S9, Supporting Information) which indicates compositional inhomogeneity between separate *Fm* $\bar{3}$ *m* disordered and *R* $\bar{3}$ *m* ordered domains for compositions beyond *x* > 0.2.

The refined structural model of *x* = 0.1 (LiNi_0.9_Ti_0.075_O_2_) in which the ordered *R* $\bar{3}$ *m* and disordered *Fm* $\bar{3}$ *m* unit cells are shown is presented in Figure 1a. The extracted unit cell parameters and volumes increase as a function of *x* in the LiNi_1–_ *_x_* Ti_3_ *_x_*_/4_O_2_ solid solution following nominal partial substitution of Ni^3+^ (0.56 Å) by the larger Ti^4+^ (0.605 Å) (Figure 1b; Figure S10 and Table S5, Supporting Information).^[^ ^6^ ^]^ This is distinct from the trend in unit cell parameters extracted from stoichiometric LiNi_1–_ *_x_* Ti*_x_* O_2_ materials synthesized under the same conditions (Figure S10, Supporting Information), which agree well with multiple previous studies of such stoichiometric materials,^[^ ^32^, ^37^ ^]^ and indicate that an entirely separate solid solution can be accessed by targeting the non‐stoichiometric LiNi_1–_ *_x_* Ti_3_ *_x_*_/4_O_2_ compositions reported here.

Interestingly, the hexagonal distortion parameter (σ) for LiNi_1–_ *_x_* Ti_3_ *_x_*_/4_O_2_ increases from *x* = 0 to a maximum of 1.00758(1) at *x* = 0.05, before decreasing in an approximately linear fashion to 1.00614(3) at *x* = 0.2 (Figure 1c). Neutron powder diffraction data, which enable more accurate refinement of site occupancies, were collected at compositions of *x* = 0, 0.05 and 0.1 (representing LiNiO_2_, LiNi_0.95_Ti_0.0375_O_2_, and LiNi_0.9_Ti_0.075_O_2_, respectively) and were used in Rietveld refinements alongside X‐ray diffraction data (Figure 1d,e; Section S6, Figures S11‐S13, and Tables S6–S8, Supporting Information). The chemical order parameter (*η*) decreases from 0.95(3) for *x* = 0 to 0.81(1) for *x* = 0.1 (Figure 1f), showing that the fraction of disordered *Fm* $\bar{3}$ *m* domains increases with increasing Ti^4+^ substitution in LiNi_1–_ *_x_* Ti_3_ *_x_*_/4_O_2_ nanocomposites. This observation of both ordered *R* $\bar{3}$ *m* and disordered *Fm* $\bar{3}$ *m* domains is distinct from the previously reported ordered *R* $\bar{3}$ *m*‐only LiNi_1–_ *_x_* Ti*_x_* O_2_ materials,^[^ ^32^, ^36^, ^50^ ^]^ and emphasizes the impact of nominally targeting compositions of LiNi_1–_ *_x_* Ti_3_ *_x_*_/4_O_2_ on the degree of chemical ordering within the structure.

High Angle Annular Dark Field (HAADF) Scanning Transmission Electron Microscopy (STEM) images of particles from *x* = 0.075 (LiNi_0.925_Ti_0.05625_O_2_) further confirms the existence of a range of multi‐domain crystallites in Ti^4+^‐substituted LiNiO_2_ (Figure 1g), consistent with the reduced chemical order parameter (*η*) extracted from powder diffraction data for Ti^4+^ substituted compositions (0.05 ≤ *x* ≤ 0.1). In contrast, STEM analyses of *x* = 0 (LiNiO_2_) particles show domains of the ordered structure only (Figure S14, Supporting Information). Coherent interfaces between ordered (*R* $\bar{3}$ *m*) and disordered (*Fm* $\bar{3}$ *m*) rock salt nanoscale domains are observed within individual crystallites of *x* = 0.075 (Figure 1g); both domains share common anion lattices but with ordered and disordered cation arrangements separated by an interfacial angle of ≈55°. In addition, two separate ordered domains intergrown at an angle of ≈70° are observed (Figure S15, Supporting Information) oriented along [100]*_H_*. Such intergrowths with ordered‐ordered interfaces,^[^ ^11^ ^]^ or ordered‐disordered interfaces,^[^ ^38^, ^41^, ^42^, ^43^, ^45^ ^]^ have been observed previously in LiNiO_2_‐based composite electrodes, however such multi‐domain crystallites are previously unreported for Ti^4+^‐substituted LiNiO_2_. Continuous rotation electron diffraction (CRED) analysis performed on a single particle of *x* = 0.075 (LiNi_0.925_Ti_0.05625_O_2_) verifies the presence of both ordered and disordered rock salt domains, and rules out the presence of spinel‐like domains (See Section S6 and Figures S16‐S18, Supporting Information).^[^ ^41^ ^]^

The coherence lengths for both *L*_hex_ and *L*_cub_ refined against powder diffraction data with the multidomain model for *x* = 0 (LiNiO_2_) are equal within error so were constrained to the same value. *L*_cub_ decreases immediately upon Ti^4+^ substitution in LiNi_1–_ *_x_* Ti_3_ *_x_*_/4_O_2_ from 208(5) nm for *x* = 0 to 91(4) nm for *x* = 0.025 and remains comparable for 0.025 ≤ *x* ≤ 0.2 (Figure S19 and Table S9, Supporting Information). In contrast, *L*_hex_ decreases consistently as *x* increases in LiNi_1–_ *_x_* Ti_3_ *_x_*_/4_O_2_ from 208(5) nm for *x* = 0 to 21.0(4) nm for *x* = 0.2 (Figure S19, Supporting Information), a trend that correlates well with the chemical order parameter *η* (Figure 1f). Primary particle sizes obtained by Scanning Electron Microscopy (SEM) imaging (Figure S20 and Table S9, Supporting Information) exhibit a comparable trend to *L*_cub_ with increasing *x*, showing a significant decrease from 282(51) nm for *x* = 0 (LiNiO_2_) to ≈100 nm for the Ti^4+^ substituted samples (0.025 ≤ *x* ≤ 0.1). These observations through HAADF‐STEM, SEM, and powder diffraction methods highlight that targeting compositions of LiNi_1–_ *_x_* Ti_3_ *_x_*_/4_O_2_, even at low levels of *x*, plays a significant role in determining the size of chemically ordered domains (*L*_hex_). With increasing *x* a larger number of the interfaces observed via STEM will be generated, leading to larger numbers of ordered domains that are, on average, smaller in size.^[^ ^48^ ^]^

### 2.2 Electrochemistry

**Figure 2:**
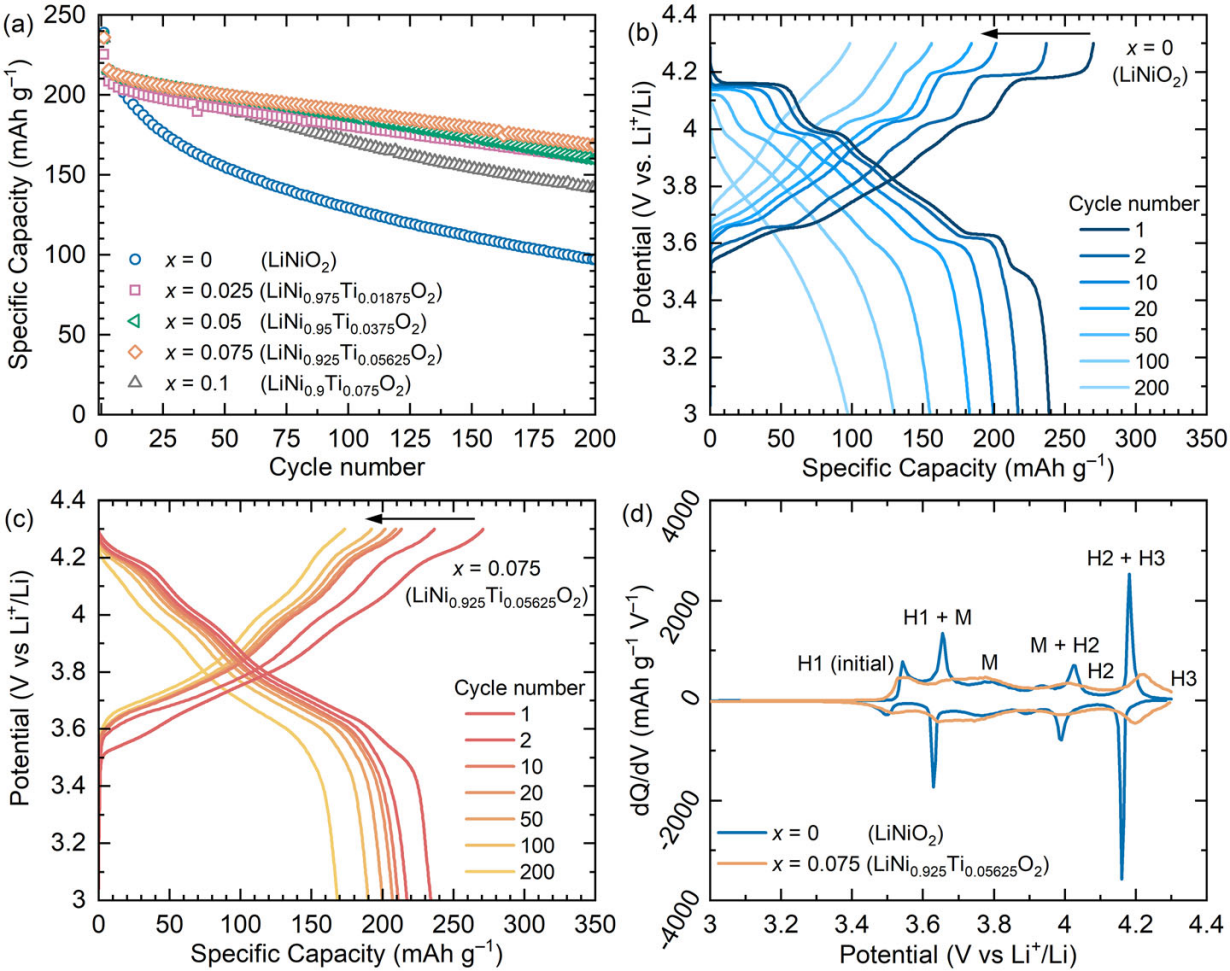
Electrochemical characterisation of LiNi_1–_ *_x_* Ti_3_ *_x_*_/4_O_2_ where 0 ≤ *x* ≤ 0.1. a) Cycling stability of LiNi_1–_ *_x_* Ti_3_ *_x_*_/4_O_2_ with cycle 1 at a current density of 20 mA g^−1^ followed by 100 mA g^−1^ for cycle 2 onwards between 3–4.3 V versus Li^+^/Li in 1 M LiPF_6_ in ethylene carbonate (EC): dimethyl carbonate (DMC) (1:1, v/v) at 30 °C. Potential profile of b) *x* = 0 (LiNiO_2_) and c) *x* = 0.075 (LiNi_0.925_Ti_0.05625_O_2_). d) Differential capacity plot on cycle 1 for *x* = 0 (LiNiO_2_) and *x* = 0.075 (LiNi_0.925_Ti_0.05625_O_2_) with features labelled based on the phase transitions known for LiNiO_2_ (H – hexagonal; M – monoclinic).

Electrochemical analysis of LiNi_1–_ *_x_* Ti_3_ *_x_*_/4_O_2_ (0 ≤ *x* ≤ 0.1) as the positive electrode materials against Li metal negative electrode is shown in **Figure** **2**. Galvanostatic cycling of *x* = 0 (LiNiO_2_) at a current density of 20 mA g^−1^ on cycle 1 followed by 100 mA g^−1^ from cycle 2 onwards shows a high initial capacity of 238 mAh g^−1^ before a continuous decline to 129 mAh g^−1^ by cycle 100 and 97 mAh g^−1^ by cycle 200, equivalent to a 59% and 45% capacity retention at 100 mA g^−1^, respectively (Figure 2a), which is comparable to previous reports.^[^ ^51^, ^52^, ^53^ ^]^ Compositions of 0.025 ≤ *x* ≤ 0.1 in LiNi_1–_ *_x_* Ti_3_ *_x_*_/4_O_2_ display similar initial capacities (ca. 230–240 mAh g^−1^) but exhibit significantly enhanced capacity retention relative to *x* = 0. The capacity of *x* = 0.075 (LiNi_0.925_Ti_0.05625_O_2_), which demonstrates the highest capacity retention at 100 mA g^−1^, decreases to 190 mAh g^−1^ by cycle 100 and 168 mAh g^−1^ by cycle 200, equivalent to capacity retentions of 87% and 77%, respectively (calculated using data collected under the same current density of 100 mA g^−1^). The LiNi_1–_ *_x_* Ti_3_ *_x_*_/4_O_2_ compositions offer substantial performance enhancement over that of the *x* = 0.1 stoichiometric LiNi_1–_ *_x_* Ti*_x_* O_2_ material (Figure S21, Supporting Information), which displays significantly reduced capacity. The cycling performance was replicated within a graphite|LiNi_0.925_Ti_0.05625_O_2_ full cell, reaching 80% of initial capacity after over 180 cycles (Figure S22, Supporting Information).

Examination of the load curves for *x* = 0 (LiNiO_2_) (Figure 2b) reveal numerous potential plateaus on charging at 3.65, 4.03, and 4.18 V versus Li^+^/Li, and on discharging at 3.5, 3.62 and 3.99 V versus Li^+^/Li which are absent in the cycle 100 data, concurrent with the observed loss of capacity. In contrast, no plateaus are observed for *x* = 0.075 (LiNi_0.925_Ti_0.05625_O_2_) from cycle 1 (Figure 2c) and this distinction is more apparent within the differential capacity plot (Figure 2d). The abrupt features observed in the differential capacity for *x* = 0 (LiNiO_2_) are associated with structural phase transitions known to have limited reversibility where the initial ordered hexagonal structure (H1) transforms to a different hexagonal structure (H3) via a monoclinic (M) and a second hexagonal (H2) phase during de‐lithiation.^[^ ^3^ ^]^ These features are significantly reduced in the differential capacity plot of *x* = 0.075 (LiNi_0.925_Ti_0.05625_O_2_), correlated to the absence of plateaus in the load curves (Figure 2c) and enhanced capacity retention observed for Ti‐substituted materials where 0.025 ≤ *x* ≤ 0.1 (Figure 2a).

### 2.3 Structural and Electrochemical Analysis of Capacity Decay

**Figure 3:**
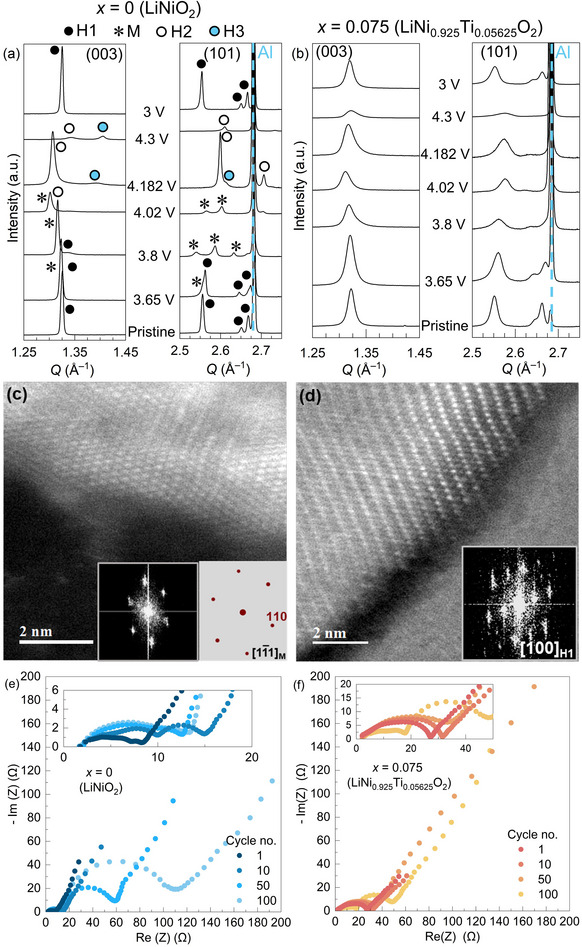
Ex situ SPXRD patterns (*Q* = 1.25–1.45 Å^−1^ and 2.5–2.75 Å^−1^) of a) *x* = 0 (LiNiO_2_) and b) *x* = 0.075 (LiNi_0.925_Ti_0.05625_O_2_) showing the (003)*_H_* and (101)*_H_* Bragg reflections (full diffraction patterns are provided in Figures S23 and S25, Supporting Information) for samples collected at different charge potentials (3.65, 3.8, 4.02, 4.182 and 4.3 V versus Li^+^/Li) and full discharge (3 V versus Li^+^/Li) on cycle 1. HAADF‐STEM images for ex situ samples of c) *x* = 0 (LiNiO_2_) and d) *x* = 0.075 (LiNi_0.925_Ti_0.05625_O_2_) after 50 cycles with corresponding fast Fourier transforms (FFT) inset. The simulated diffraction pattern of the monoclinic phase of Li*_x_* NiO_2_ is shown in (c) for comparison. Impedance spectra (collected over a frequency range of 0.5 MHz to 1 mHz) on cycles 1, 10, 50 and 100 for e) *x* = 0 (LiNiO_2_) and f) *x* = 0.075 (LiNi_0.925_Ti_0.05625_O_2_).

Ex situ SPXRD data were collected at a range of charge potentials for *x* = 0 and 0.075 (3.65, 3.8, 4.02, 4.182 and 4.3 V versus Li^+^/Li) following cycle 1 and after discharge to 3 V versus Li^+^Li (Figures S23–S25, Supporting Information) to investigate this significantly improved capacity retention and suppression of features associated with phase transitions observed for *x* = 0.075 (LiNi_0.925_Ti_0.05625_O_2_). Significant changes to the diffraction patterns at each charge potential are observed for LiNiO_2_, associated with the abrupt features observed in the differential capacity data (Figure 2d), and each of the three intermediate phases (M, H2 and H3) are observed at the relevant points of charge in the diffraction data (**Figure** **3**a; Figures S23,S24 and Table S10, Supporting Information).^[^ ^54^ ^]^ In contrast, only reflections associated with the *R* $\bar{3}$ *m* structure in *x* = 0.075 (LiNi_0.925_Ti_0.05625_O_2_) are visible at different states of charge/discharge for cycle 1, indicating that no structural transitions take place (Figure 3b; Figures S25,S26 and Table S11, Supporting Information). Ex situ differential scanning calorimetry (DSC) measurements of LiNiO_2_ and LiNi_0.925_Ti_0.05625_O_2_ in the delithiated state shows comparable thermal stabilities (Figure S27, Supporting Information). After 50 cycles, the reflections corresponding to the monoclinic phase persist in the diffraction pattern of *x* = 0 (LiNiO_2_) indicating that the M‐H1 phase transition is not fully reversible upon discharge (Figure S28a and Table S12, Supporting Information), correlated to the poor capacity retention (Figure 2a). Whereas for *x* = 0.075 (LiNi_0.925_Ti_0.05625_O_2_), there is minimal change to the diffraction pattern following 50 cycles (Figure S28b and Table S12, Supporting Information). The unit cell and structural distortion parameters are extracted from *x* = 0.075 over the course of the first cycle (Figure S26 and Table S11, Supporting Information) using the same multidomain hexagonal structural model described above. There are remarkably small changes in the unit cell parameters (*a* and *c*) and volume (*V*) between pristine *x* = 0.075 and the charged sample at 4.3 V versus Li^+^/Li where *a* decreases by 0.53%, *c* increases by 0.34% and *V* decreases by 0.72%. In contrast, significantly larger changes are observed for the *R* $\bar{3}$ *m* (H1) phase in *x* = 0 (LiNiO_2_) (Figure S24 and Table S10, Supporting Information) equating to a 9.4% decrease in unit cell volume from pristine to 4.02 V, consistent with the 6–10% range reported for LiNiO_2_.^[^ ^55^ ^]^ Though the phase transitions were suppressed in a small number of LiNi_1–_ *_x_* Ti*_x_* O_2_ materials that retain a single‐phase *R* $\bar{3}$ *m* structure during cycling, the observed changes in unit cell parameters were significantly larger than those for *x* = 0.075 (LiNi_0.925_Ti_0.05625_O_2_).^[^ ^32^, ^34^ ^]^ The composition *x* = 0.075 (LiNi_0.925_Ti_0.05625_O_2_) displays extremely low structural strain during electrochemical cycling, with a smaller change in unit cell parameters than other low‐strain LiNiO_2_‐based materials.^[^ ^42^, ^43^, ^45^ ^]^

HAADF‐STEM images were collected after 50 cycles for both *x* = 0 (LiNiO_2_) (Figure 3c,*x*) = 0.075 (LiNi_0.925_Ti_0.05625_O_2_) (Figure 3d). Fast Fourier transformations (FFT) of the images of *x* = 0 (LiNiO_2_) crystallites show reflections characteristic of the monoclinic structure (Figure 3c) and those characteristic of the ordered *R* $\bar{3}$ *m* structure (Figure S29, Supporting Information), which is consistent with the SPXRD data (Figure 3a, Figure S28a, Supporting Information). In contrast, HAADF‐STEM images for *x* = 0.075 (LiNi_0.925_Ti_0.05625_O_2_) demonstrate the persistence of the ordered *R* $\bar{3}$ *m* and disordered *Fm* $\bar{3}$ *m* structures both in the bulk material, consistent with SPXRD, and at the crystallite surface (Figure 3d; Figure S28b, S30, and S31, Supporting Information). These observations confirm that the property‐determining phase transitions of parent *x* = 0 (LiNiO_2_) are suppressed in *x* = 0.075 (LiNi_0.925_Ti_0.05625_O_2_), which is consistent with the higher capacity retentions observed for LiNi_1–_ *_x_* Ti_3_ *_x_*_/4_O_2_ (0.025 ≤ *x* ≤ 0.1) materials.

Based on the total resistance of the half‐cells, electrochemical impedance spectroscopy (Figure 3e,f) shows that *x* = 0.075 (LiNi_0.925_Ti_0.05625_O_2_) exhibits a high interfacial stability compared to *x* = 0 (LiNiO_2_), also consistent with the observations from HAADF‐STEM. For *x* = 0, the total cell resistance increases significantly after 50 and 100 cycles (an increase of 191% from cycle 50 to 100), whereas for *x* = 0.075, this increase in resistance is much lower (an increase of 145% from cycle 50 to 100).

### 2.4 Electrokinetics and Structural Stability in LiNi_1–_ *_x_* Ti_3_ *_x_*_/4_O_2_

**Figure 4:**
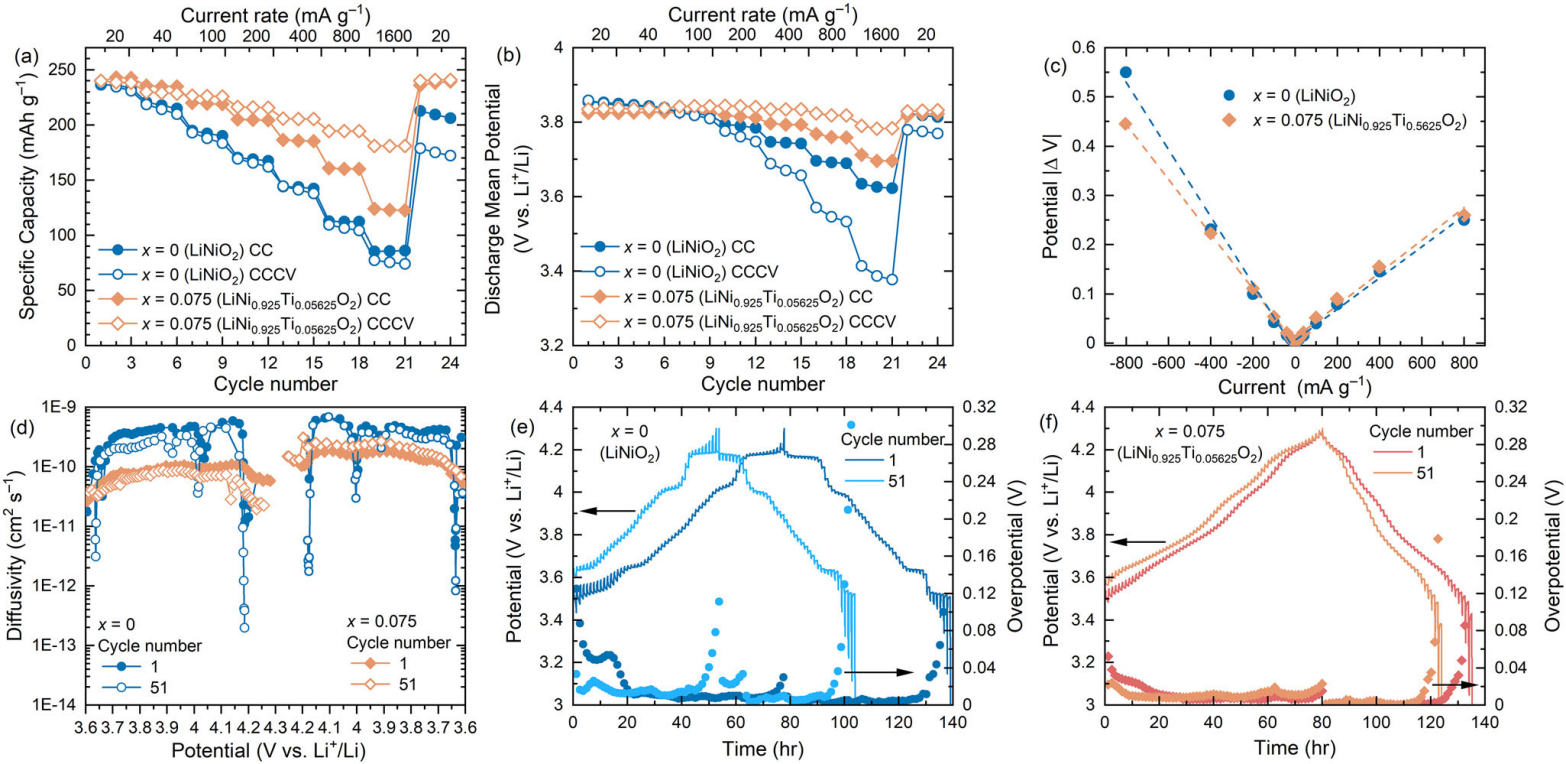
a) Rate capability and b) the corresponding discharge mean potential for *x* = 0 (LiNiO_2_) and *x* = 0.075 (LiNi_0.925_Ti_0.05625_O_2_) under constant current (CC) and constant current constant voltage (CCCV) modes using 1 M LiPF_6_ in EC:DMC (1:1, v/v). c) Direct current internal resistance plot for different current densities. d) Diffusivity plot for cycles 1 and 51 extracted via galvanostatic intermittent titration technique (GITT). Voltage profiles (lines) and overpotential (filled symbols) for cycles 1 and 51 for e) *x* = 0 and f) *x* = 0.075.

The enhanced performance of *x* = 0.075 (LiNi_0.925_Ti_0.05625_O_2_) is investigated further by assessing the structural stability and Li^+^ diffusion kinetics at deeply delithiated states (**Figure** **4**) and compared against *x* = 0 (LiNiO_2_). A constant voltage (CV) charge step (until the current decreases to below 5 mA g^−1^) is introduced once a charge potential of 4.3 V versus Li^+^/Li is reached at the galvanostatic constant current (CC) step. Such CCCV protocols typically lead to significantly accelerated degradation and an irreversible loss in performance in Ni‐rich rock salt positive electrode materials.^[^ ^56^, ^57^ ^]^ Pristine *x* = 0 (LiNiO_2_), with and without this CV charge step, shows lower capacity retention at high current rates of >40 mA g^−1^ compared to *x* = 0.075 (LiNi_0.925_Ti_0.05625_O_2_), with capacities of 80 and 181 mAh g^−1^, respectively, reached at a current rate of 1600 mA g^−1^ under CCCV (Figure 4a; Figure S32, Supporting Information). The enhanced structural stability of *x* = 0.075 enables extensive de‐lithiation by CCCV and the recovery of the same initial capacity (240 mAh g^−1^) at the final current rate of 20 mA g^−1^ (cycle 22 to 24), which is previously unreported for Ti‐substituted LiNiO_2_. LiNiO_2_ shows the lowest mean discharge potential of ≈3.4 V versus Li^+^/Li when cycled under CCCV at 1600 mA g^−1^ (Figure 4b), which is not recovered upon returning to a lower current density of 20 mA g^−1^. In contrast, for *x* = 0.075 the average discharge potential is ≈3.8 V and the initial potential is recovered following the high current steps. This enhanced stability and reversible performance under deep delithiation represents a significant advance for Ni‐rich ordered rock salt materials.

Direct current internal resistance (DCIR) analyses (using current pulses at 40% state of charge) is used to identify the internal resistance at different current densities (Figure 4c; Figure S33, Supporting Information). During discharge, a deviation between *x* = 0 and *x* = 0.075 is observed above –400 mA g^−1^. At –800 mA g^−1^ the difference in ΔV between the two compounds is >100 mV, whereby *x* = 0.075 displays the lower ΔV, explaining the enhanced performance at higher rates.

Galvanostatic intermittent titration technique (GITT) was used to compare Li^+^ kinetics of *x* = 0 and *x* = 0.075 on cycle 1 and cycle 51 (Figure 4d), and diffusion coefficients across the potential range were determined to be ≈3 × 10^−10^ and ≈1 × 10^−10^ cm^2^ s^−1^, respectively, consistent with pristine and doped LiNiO_2_ materials (Table S13, Supporting Information).^[^ ^7^, ^11^, ^14^, ^15^, ^58^, ^59^ ^]^ The slightly lower diffusivity measured for *x* = 0.075 results from the material being an intergrowth of ordered and disordered rock salt domains,^[^ ^60^ ^]^ however this stabilises the diffusion across a range of charge potentials which also reflects the enhanced interfacial stability observed via electrochemical impedance spectroscopy (Figure 3e,f). In contrast, several dramatic drops in diffusivity (of approximately three orders of magnitude) are observed for *x* = 0 (LiNiO_2_) which coincide with the phase transitions discussed above (Figure 2d). Note that best practice of GITT has been proposed recently for accurate determination of Li^+^ diffusion coefficients, together with other complementary methods.^[^ ^61^, ^62^ ^]^ This is also apparent in the increased overpotential observed for *x* = 0 compared to *x* = 0.075 at the end of charge and discharge (Figure 4e,f). The observed phase transitions in *x* = 0 impose significant polarisation and lower Li^+^ ion kinetics, especially on the H2‐H3 transition during charge and M‐H1 transition during discharge as observed previously. This is consistent with the observations from STEM and SPXRD that demonstrate the irreversibility once the monoclinic structure is formed in *x* = 0 (Figure 3c; Figure S28 and Table S12, Supporting Information).

### 2.5 High Current and High Loading Capability of *x* = 0.075 (LiNi_0.925_Ti_0.05625_O_2_)

**Figure 5:**
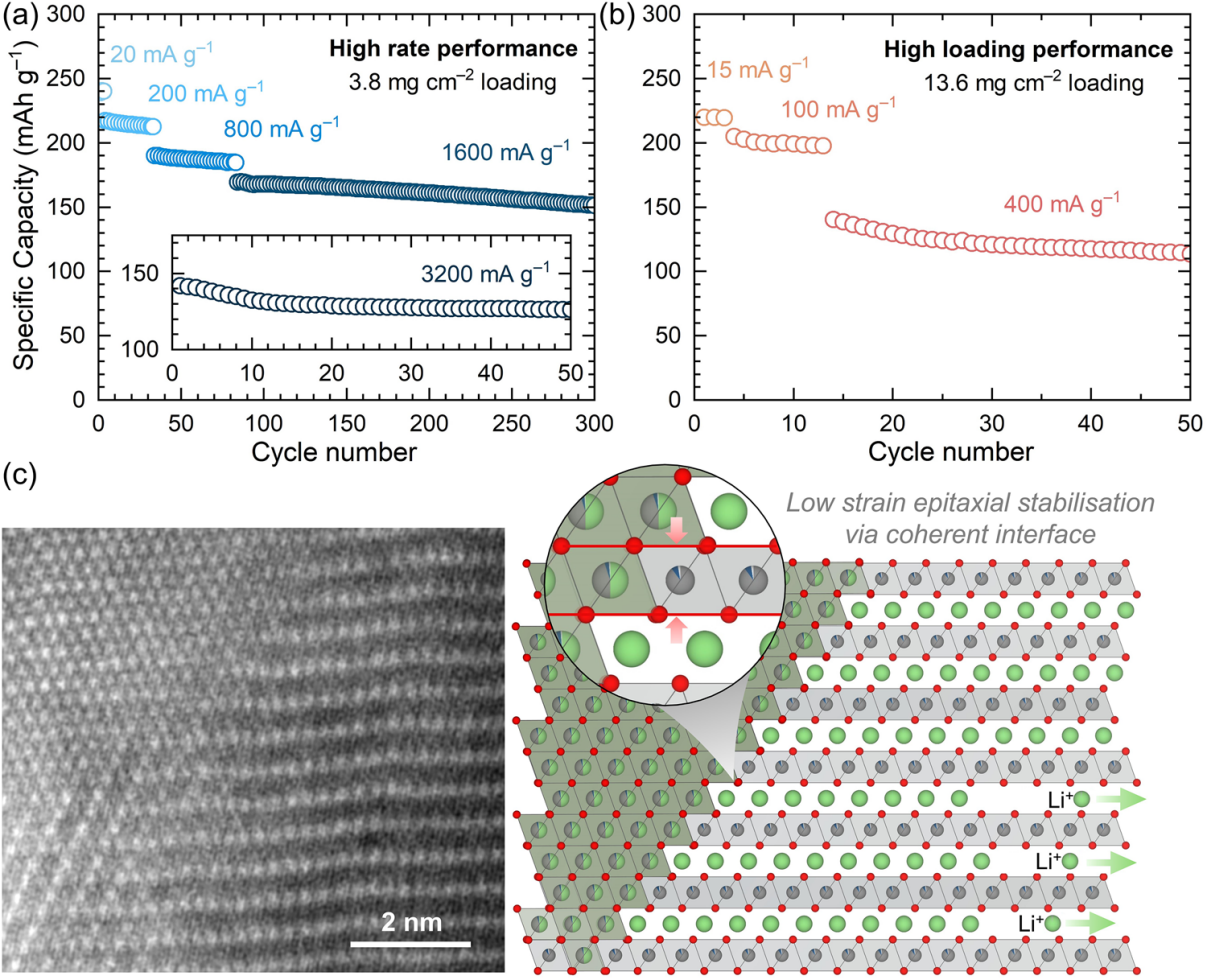
a) High rate and long term cycle performance for *x* = 0.075 (LiNi_0.925_Ti_0.05625_O_2_) tested at 20, 200, 800 and 1600 mA g^−1^ (loading amount ≈3.76 mg cm^−2^). b) Cycling performance of *x* = 0.075 (LiNi_0.925_Ti_0.05625_O_2_) high electrode loading (13.6 mg cm^−2^) tested at 15, 100, and 400 mA g^−1^ (or 0.204, 1.36 and 5.44 mA cm^−2^ for areal current densities) in 1 M LiPF_6_ in EC:ethyl methyl carbonate (EMC) 3:7 with 2 wt.% vinylene carbonate (VC). c) Schematic of a coherent interface between disordered and ordered rock salt domains observed by HAADF‐STEM in *x* = 0.075 (LiNi_0.925_Ti_0.05625_O_2_).

The composition of *x* = 0.075 (LiNi_0.925_Ti_0.05625_O_2_) demonstrates high performance even in real‐world device conditions which were evaluated under high current densities (400 mA g^−1^) and high loadings (≥3 mAh cm^−2^ areal capacity) using an additive‐containing electrolyte 1 M LiPF_6_, EC: ethyl methyl carbonate (EMC) 3:7, 2 wt.% vinylene carbonate (VC). Use of VC for cycling *x* = 0.075 (LiNi_0.925_Ti_0.05625_O_2_) at current densities of 100 mA g^−1^ improves the cycling retention (at 100 cycles) from 87% to 93% (Figure S34, Supporting Information) over the range of 3–4.3 V versus Li^+^/Li. Though the initial discharge capacities were slightly greater, reaching 256 mAh g^−1^ the cycling retention is reduced to 70% after 100 cycles when a wider potential range of 3–4.5 V versus Li^+^/Li is used (Figure S35, Supporting Information). This reduced cycling stability is associated with larger volume changes which occur between 4.3 and 4.5 V versus Li^+^/Li for *x* = 0.075 (LiNi_0.925_Ti_0.05625_O_2_) observed via ex situ PXRD, demonstrating that Ti^4+^ substitution through LiNi_1–_ *_x_* Ti_3_ *_x_*_/4_O_2_ shifts the structural transition of LiNiO_2_ to higher potentials (Figure S36 and Table S14, Supporting Information). High‐capacity retention is also achieved at a range of current densities (increasing from 20 to 1600 mA g^−1^), showing a stable capacity of ≈170 mAh g^−1^ over 200 cycles at 1600 mA g^−1^ (**Figure** **5**a). Exceptional capacity retention (125 mAh g^−1^) was achieved even at rates as high as 3200 mA g^−1^ (Figure 5a inset). Furthermore, *x* = 0.075 was tested at increased loadings (13.6 mg cm^−2^, or ≈3.4 mAh cm^−2^ theoretical areal capacity) and displays a capacity greater than 100 mAh g^−1^ at a current density of 400 mA g^−1^ (Figure 5b), yielding an areal capacity of 1.5 mAh cm^−2^. The measured performance at these electrode mass loadings approaches the practical loadings required for commercial cells, and provides a clear demonstration for LiNi_1–_ *_x_* Ti_3_ *_x_*_/4_O_2_ materials for practical application as positive electrodes.^[^ ^63^, ^64^ ^]^

Ex situ SPXRD data collected for both *x* = 0 (LiNiO_2_) and *x* = 0.075 (LiNi_0.925_Ti_0.05625_O_2_) discharged to 3 V versus Li^+^/Li after 50 cycles under 3200 mA g^−1^ further verify the enhanced structural stability offered by *x* = 0.075 (LiNi_0.925_Ti_0.05625_O_2_) over the parent material. LiNiO_2_ transforms predominantly to the monoclinic phase whereas no evidence for phase transitions is observed for *x* = 0.075 (LiNi_0.925_Ti_0.05625_O_2_), retaining its original structure that can be described by the multidomain hexagonal *R* $\bar{3}$ *m* model described above (Figure S38 and Table S15, Supporting Information).

The excellent performance of LiNi_1–_ *_x_* Ti_3_ *_x_*_/4_O_2_ materials, optimized in particular at *x* = 0.075, is a culmination of several property‐controlling structural factors which are shown schematically in Figure 5c. By targeting Ni‐rich compositions through LiNi_1–_ *_x_* Ti_3_ *_x_*_/4_O_2_ a nanocomposite with multi‐domain crystallites is obtained, previously unreported for Ti^4+^‐substituted LiNiO_2_. The homogeneous distribution of transition metals within the bulk material affords coherent interfaces via an anion sublattice common to both the ordered and disordered domains. This ensures that the cycling‐induced strain resulting from the deintercalation and intercalation of Li^+^ from the ordered *R* $\bar{3}$ *m* domains is alleviated via epitaxial stabilization with the disordered *Fm* $\bar{3}$ *m* domains to yield a low‐strain positive electrode material. Such a composite combines the benefits of both structures which enables the long‐term cycling stability, high‐rate and high‐loading performance previously unseen for LiNiO_2_‐based positive electrode materials.

## 3 Conclusion

By targeting the specific compositions of LiNi_1–_ *_x_* Ti_3_ *_x_*_/4_O_2_ (0 ≤ *x* ≤ 0.3), Ti^4+^ substitution into LiNiO_2_ yields nanocomposite materials with separate domains of ordered *R* $\bar{3}$ *m* and disordered *Fm* $\bar{3}$ *m* rock salt structures within single crystallites in compositions where *x* > 0, previously unseen for Ti‐substituted LiNiO_2_, where the degree and size of the ordered domains decreases with increasing *x*. The ordered and disordered domains, which are compositionally homogeneous, share a common anion sublattice via coherent interfaces which enables accurate structural refinement of both using a multidomain hexagonal model and single unit cell. The synergic combination of high Li^+^ ion diffusion offered by the ordered *R* $\bar{3}$ *m* structure and structural stability provided by the *Fm* $\bar{3}$ *m* structure within multi‐domain crystallites suppresses the capacity‐limiting phase transitions that are known to plague LiNiO_2_‐based positive electrode materials and establishes low strain (<1%) behavior and high cycling stability in LiNi_1–_ *_x_* Ti_3_ *_x_*_/4_O_2_ materials during deintercalation. As a result, the highest performing composition, *x* = 0.075 (LiNi_0.925_Ti_0.05625_O_2_) delivers a stable capacity of 203 mAh g^−1^ after 100 cycles and 93% capacity retention at a current density of 100 mA g^−1^. This enhanced stability enables cycling to deep delithiation under CCCV conditions and is retained even at high current densities of 3200 mAh g^−1^, representing a significant advance over LiNiO_2_. Excellent performance is retained at increased cell electrode mass loadings (13.6 mg cm^−2^) approaching those used in commercial cells for practical applications. The impact of the targeted composition on the structures of rock salt materials, as exemplified by the nominally vacancy‐based series studied here, enables access to new chemistries and enhanced performance beyond those of the well‐explored stoichiometric solid solutions, offering a promising new route for the design of high‐performance rock salt positive electrode materials.

## 4 Experimental Section

### Synthesis of LiNi_1–_ *_x_* Ti_3_ *_x_*_/4_O_2_

Starting materials of TiO_2_ (Anatase, 99.7%, Sigma Aldrich), LiOH·H_2_O (99.995%, Thermoscientific) and Ni(OH)_2_ (Sigma Aldrich) were used. LiOH·H_2_O was dried overnight under dynamic vacuum (<10^−4^ mbar) at 170 °C (see Figure S2 and Table S2, Supporting Information). TiO_2_ was dried overnight at 200 °C in air. Following these drying procedures, all starting materials were handled inside an argon‐filled glovebox (O_2_ < 0.1 ppm, H_2_O < 0.1 ppm).

LiNi_1–_ *_x_* Ti_3_ *_x_*_/4_O_2_ (0 ≤ *x* ≤ 0.3) and LiNi_1–_ *_x_* Ti*_x_* O_2_ (0 ≤ *x* ≤ 0.2) powders were synthesized by ball‐milling stoichiometric amounts of the starting materials (without Li excess) to yield a total mass of 3 g of the target compositions. The precursors were transferred into 45 mL sealed zirconia jars with seven 10 mm diameter zirconia balls. Ball milling was performed at 350 rpm for 150 min (10 min milling, 15 min rest) under Ar atmosphere (FRITSCH PULVERISETTE 7 premium line Planetary Ball Mill). The powders were then placed in an alumina crucible inside a quartz tube which was sealed at both ends using Swagelok end caps. Final black powders were obtained by annealing the ball‐milled mixtures at 700 °C for 20 h under flowing dry O_2_ gas with a flow rate of 100 mL min^−1^.

### Diffraction and Refinement

Routine analysis of polycrystalline powder samples was undertaken using a Bruker D8 Discover diffractometer with monochromatic Cu radiation (Kα_1_, λ = 1.54056 Å) in Debye–Scherrer transmission geometry with sample powders loaded into 0.3 mm diameter borosilicate glass capillaries and sealed in Ar atmosphere. Accurate unit cell parameters were extracted for the stoichiometric LiNi_1–_ *_x_* Ti*_x_* O_2_ (0 ≤ *x* ≤ 0.2) compositions via the internal standard method using NIST SRM 660c LaB_6_ powder.

Synchrotron X‐ray powder diffraction (SPXRD) data were collected at the I11 beamline at Diamond Light Source (Oxfordshire, U.K.) using a position sensitive detector (PSD, λ = 0.825005 Å). For ex situ data collection, electrodes were recovered from the cells and sealed inside aluminum pouches inside an inert atmosphere glove box. Data were collected using a position sensitive detector (PSD, λ = 0.823899 Å).

Time‐of‐flight neutron diffraction data were collected on a ^7^Li enriched samples of LiNi_1–_ *_x_* Ti_3_ *_x_*_/4_O_2_ (*x* = 0, 0.05 and 0.1) to minimize the effects of absorption. The ^7^Li enriched samples were synthesized as described above using ^7^LiOH·H_2_O dried overnight under dynamic vacuum (<10^−4^ mbar) at 170 °C. Time‐of‐flight neutron powder diffraction (NPD) data were collected at room temperature on the Nanoscale Ordered Materials Diffractometer (NOMAD) at Oak Ridge National Laboratory. Samples were sealed in vanadium cylindrical cans in an argon‐filled glovebox. Combined Rietveld refinements against SPXRD and NPD data were carried out using Topas (Version 6) software.

### Infrared Spectroscopy

Infrared spectroscopy measurements were performed in ATR mode using a Nicolet iS50 FTIR spectrometer with CsI beamsplitters (ThermoFisher Scientific) inside a nitrogen containing glovebox (O_2_, H_2_O < 0.1 ppm).

### Inductively Coupled Plasma Mass Spectroscopy

Inductively coupled plasma mass spectrometry (ICP‐MS) was used to determine cation contents of the as‐made materials. Solutions, measured in triplicate, were prepared by dissolving 10 mg of each powder into 2 mL concentrated HCl in an autoclave at 100 °C for 12 hours before diluting to 50 mL with ultra‐pure water. Measurements were collected on Perkin Elmer ICP‐MS NexION 2000.

### Magnetic Measurements

Powders of LiNiO_2_, LiNi_0.9_Ti_0.075_O_2_ and stoichiometric LiNi_0.9_Ti_0.1_O_2_ were pressed into pellets. A precisely measured portion of each pellet (≈2–8 mg) was loaded into custom‐made quartz tube and sealed under high vacuum (≈2 × 10^−5^ Torr). Magnetisation measurements were carried out using a commercial superconducting quantum interference device magnetometer MPMS3 (Quantum Design, USA). The contribution of the quartz tube to the magnetization was confirmed to be negligible prior to measuring the samples. Zero‐field cooled (ZFC) and field cooled (FC) measurements at 100 Oe and subsequent field‐cooled measurement at 45 kOe were performed from 2 to 300 K.

### Electron Microscopy

Sample morphologies were investigated via scanning electron microscopy (SEM) using a TESCAN S8000 microscope. Samples were dispersed onto carbon tape attached to a brass stub and coated with a thin layer of platinum before imaging. The samples were transported from the glovebox to the SEM using a Quorum transfer holder for air sensitive materials. Particle sizes were estimated directly from images using ImageJ.^[^ ^65^ ^]^

Transmission Electron Microscopy Energy‐dispersive X‐ray spectroscopy (TEM‐EDX) data were collected for all compositions using a JEOL2100+ operating at 200 kV equipped with SDD detector from Oxford Instruments (Model: X‐Max 65T with a 65 mm^2^ surface area detection). Samples were dispersed on carbon coated copper TEM grids and were inserted using a single tilt holder for air sensitive materials. Data acquisition and analysis were performed using Aztec software. Correction factors were determined by measuring standards for each chemical element. Small, isolated particles were picked for EDX analysis to avoid collecting data from agglomerates and 10 particles were measured to determine homogeneity.

The microstructures of the pristine samples LiNi_1–_ *_x_* Ti_3_ *_x_*_/4_O_2_ (*x* = 0, 0.075 and 0.1) were investigated by High Angle Annular Dark Field (HAADF) Scanning Transmission Electron Microscopy (STEM). For all samples, the images were collected using a Cs corrected TEM/STEM Jeol 2100FCs microscope operating at 200 kV. For LiNiO_2_, sample was dispersed on carbon coated copper TEM grids. LiNi_1–_ *_x_* Ti_3_ *_x_*_/4_O_2_ (0 ≤ *x* ≤ 0.1) particles were air‐ and beam‐sensitive and have the tendency to form large aggregates that were hard to image under the electron microscope. To separate the aggregates and to allow the study of single particles, ≈5 mg of LiNi_0.925_Ti_0.05625_O_2_ (*x* = 0.075) were added to 4 mL of anhydrous methanol and gently ground with a pestle and mortar for 3–5 min inside an argon filled glovebox (O_2_ < 0.1 ppm, H_2_O < 0.1 ppm). The suspension was transferred on a vial, sealed and sonicated for 30 s in a water bath. The final suspension was left decanting overnight inside the glovebox and the following morning an aliquot from the supernatant was deposited on a TEM grid. More than one grid can be prepared at the same time. Storing them in the glovebox allows multiple measurements to be performed on the same material. In order to prevent carbon deposition, the grid was then dried overnight under vacuum in the antechamber at 50 °C before STEM imaging. A FIB lamella was prepared for LiNi_0.9_Ti_0.075_O_2_ (*x* = 0.1). Sample was dispersed on an SEM stub with an adhesive carbon tape. Focused Ion Beam (Ga‐FIB) was performed on a dual beam Tescan S8000 SEM/FIB, equipped with a Quorum PP3010 Cryo‐FIB/SEM transfer pre‐chamber for air sensitive materials. First, a protective carbon and Pt layers (20 × 4 × 1 µm) were deposited on the selected area (30 keV, 150 pA). Then, the area was trenched with the FIB (30 keV, 10 nA). A primary thinning was done (30 keV, 3 nA) to obtain a ≈2 µm thick lamella. The lamella was then connected to the TEM grid and thinned at lower voltages and currents (down to 2 keV) for imaging.

Ex situ STEM images were collected on the same instrument on LiNiO_2_ and LiNi_0.925_Ti_0.05625_O_2_ (*x* = 0.075) discharged to 3 V versus Li^+^/Li after 50 cycles. For both samples, lamellae were prepared on a Thermo‐Fisher Helios G5 Cx Ga‐FIB‐SEM system operating at 30 keV. A layer of ≈200 nm of Carbon was deposited with the electron beam (E‐beam) to protect the surface from the Ion beam (I‐beam). Then, layers of Carbon and Pt (20 × 4 × 1 µm) were deposited on the selected area I beam (30 keV, 230 pA). A primary thinning was done (30 keV, 0.23 nA) to obtain a ≈2 µm thickness lamella. The lamella was then connected to the TEM grid and thinned at lower voltages and currents (down to 2 keV) for imaging. A Thermo‐Fisher vacuum transfer holder was used to transfer the samples between the glovebox and the Ga‐FIB to avoid air exposure.

### Continuous Rotation Electron Diffraction

Continuous Rotation Electron Diffraction (CRED) measurements were collected on a sample of LiNi_0.925_Ti_0.05625_O_2_ (*x* = 0.075) that was prepared as described above. CRED data were collected on single particles using a 200KV JEOL 2100+ equipped with a GATAN Rio Camera using insteaDMatic software.^[^ ^66^ ^]^ Energy Dispersive X‐ray spectroscopy (EDX) measurements were performed using an X‐Max detector from Oxford instruments data on the particles to confirm their composition prior to diffraction measurement. CRED data were initially processed using REDp software^[^ ^67^, ^68^ ^]^ and then further reduced using micro‐ED CrysAlisPro v43 from Rigaku. Multiple datasets merging and scaling was performed using XPREP software from Bruker. Space group and initial crystal structure was derived using ShelXT.^[^ ^69^ ^]^ The suggested space group symmetry was confirmed through the study of the diffraction systematic absences. Final crystal structures were refined using Olex2 from OlexSys.^[^ ^70^ ^]^

### Electrode Preparation

LiNi_1–_ *_x_* Ti_3_ *_x_*_/4_O_2_ (0 < *x* < 0.1) were mixed with carbon (Super C, Timcal) and polyvinylidene fluoride (PVDF, Kynar) in N‐methyl‐2‐pyrrolidone (NMP, anhydrous, Sigma–Aldrich) solvent. Further dehydration of NMP was achieved via use of molecular sieves (Alfa Aesar). The carbon conductive additive was dried at 250 °C under vacuum for one day, to remove trace water. The slurry ratio was 90:5:5 wt.% and slurries were cast onto Al foil inside the Ar‐filled glovebox before drying the cast electrodes at 80 °C under vacuum. The average loading amount of LiNi_1–_ *_x_* Ti_3_ *_x_*_/4_O_2_ was in the range of 2–5 mg cm^−2^. For the high ‐rate tests, electrode loading amounts were fixed at 3.76 mg cm^−2^, which were denoted in figures or captions. For higher electrode mass loading tests, the average loading amount was 13.6 mg cm^−2^ (which corresponds to 3 mAh cm^−2^ for areal capacity).

For full‐cell measurements, negative electrodes were prepared under ambient atmosphere using aqueous dispersions of graphite active material (SFG44, Imerys, synthetic graphite, average particle size 44 µm) with Super C carbon, sodium carboxymethyl cellulose, and styrene‐butadiene rubber with a slurry ratio of 94:1.7:1.7:2.6 wt.%. After successive mixing steps with controlled cooling between each step, the aqueous slurry was cast onto Cu foil. After initial drying, punched electrodes were further dried at 110 °C under vacuum in a Buchi drying oven and transferred into the Ar glovebox without exposure to ambient atmosphere. The loading amount of SFG44 graphite was in the range of 1.6–2.9 mg cm^−2^.

### Cell Cycling Tests

All components were handled and assembled within an argon containing glovebox (O_2_ <0.1 ppm, H_2_O <0.1 ppm). Electrodes, separators and electrolytes were stored within the glovebox. All electrochemical measurements, with the exception of full‐cell investigations, were conducted within coin cells, with Li metal foil (99.9%, 0.38 mm thickness, Sigma Aldrich) used as both the counter and reference electrodes. 1 M LiPF_6_ in ethylene carbonate (EC): dimethyl carbonate (DMC) = 1:1, volume ratio (BASF) was used as the baseline electrolyte and 1 M LiPF_6_, 2 wt.% vinylene carbonate in EC: ethyl methyl carbonate (EMC) = 3:7, vol % (Soulbrain) was used as the additive containing electrolyte. Pre‐cycling at 20 mA g^−1^ was performed for one cycle followed by 100 mA g^−1^ for all following cycles to assess all LiNi_1–_ *_x_* Ti_3/4_ *_x_* O_2_ (*x* = 0, 0.025, 0.5, 0.075, 0.1) materials. Three pre‐cycles at a rate of 15 mA g^−1^ were performed before the high rate or high loading tests were carried out. Constant voltage (CV) processes were added after 4.3 V versus Li^+^/Li charging by constant current (CC) until the current reached 5 mA g^−1^ for the CCCV tests. For high‐rate tests, the current densities were increased from 20, 40, 100, 200, 400, 800, to 1600 mA g^−1^ and reversibly back to 20 mA g^−1^ with and without the constant voltage processes. For high voltage range tests, the upper potential limit was extended to 4.5 V versus Li^+^/Li, maintaining the CCCV regime on charging. The mean discharge potential was calculated by integrating the discharge voltage profile followed by dividing into specific capacity. All electrochemical tests and analyses were done at 30 °C using a multichannel potentiostat (Maccor).

For full cell experiments, cells were prepared as above but the Li metal foil electrode was replaced by a graphite (SFG44) negative electrode and the additive containing electrolyte (1 M LiPF_6_ with 2 wt.% vinylene carbonate in EC:EMC (3:7 vol%)) was used. Electrode masses were balanced such that the capacity ratio between negative (Q_an_) and positive (Q_cat_) active materials was roughly Q_an_/Q_cat_ ≈1.03–1.1, calculated using the first half‐cycle capacities in Li half‐cells of positive and negative electrodes (i.e., 271 mAh g^−1^ and 376 mAh g^−1^ for LiNi_0.925_Ti_0.05625_O_2_ and SFG 44, respectively). SFG44 was selected for use herein due to the low irreversible capacity on the first cycle. After sealing inside the Ar glovebox, the cells were transferred to the potentiostat, housed at 30 °C as above. All cycling currents were normalized to the mass of positive electrode active material. Cells were first charged slowly (ca. 6 mA g^−1^) up to 1.5 V (E_cell_) to minimize corrosion. Cells were then rested for 10 h for full electrode wetting and then cycled between 4.25 V and 2.95 V (E_cell_), employing CCCV on charge and CC only discharge.

Full cells were initially cycled through three formation cycles, 1 cycle at 10 mA g^−1^, and 2 cycles at 15 mA g^−1^, and then cycled continuously at 100 mA g^−1^.

### Differential Scanning Calorimetry (DSC)

Heat flux profiles were measured on ex situ samples of LiNiO_2_ and LiNi_0.925_Ti_0.05625_O_2_ collected after charging to 4.3 V at 20 mA g^−1^ in Swagelok cell. Collected powders were then washed with dimethyl carbonate (DMC) and left to dry. Sample were sealed in a 25 µL aluminum crucible cold‐welded under an Ar atmosphere (O_2_ < 0.1 ppm, H_2_O < 0.1 ppm). Data were collected from 25 to 300 °C at heating and cooling rates of 10 °C min^−1^ under a flow of helium (50 mL min^−1^) using a Netzsch DSC 404 F1 differential calorimeter.

### Ex Situ Analysis for Cells Cycled 4.5 V

Ex situ samples of LiNiO_2_ and LiNi_0.925_Ti_0.05625_O_2_ were collected after charging to 4.5 V versus Li^+^/Li (fully charged) and after charging to 4.5 V and discharging to 3 V versus Li^+^/Li (fully discharged) at 20 mA g^−1^ in Swagelok cell. Collected powders were then washed with dimethyl carbonate (DMC) and left to dry. Samples were then loaded into 0.5 mm diameter borosilicate glass capillaries and sealed in Ar atmosphere. PXRD patterns were measured in Debye–Scherrer transmission geometry using a Cu Bruker diffractometer (λ = 1.541874 Å).

### Electrochemical Analyses

Electrochemical Impedance Spectroscopy (EIS) was performed after cycles 1, 10, 50, and 100 with the potential taken back to 3.9 V versus Li^+^/Li. Spectra were collected with a 10 mV AC amplitude from 0.5 MHz to 1 mHz. Before testing the Direct Current Internal Resistance (DCIR), cells were first charged to 40% state of charge (ca. 3.65 V). After one hour of rest time, a DC pulse was then imposed for 10 s followed by a further 20 min of rest time. Positive (+), and negative (−) direct currents were imposed alternately using the sequential current rates of 5, 10, 20, 40, 100, 200, 400, 800 mA g^−1^. For the Galvanostatic Intermittence Titration Technique (GITT), Li ion diffusivity (diffusion coefficient) was calculated by the following equation.

(1) $$D=\frac{4}{\pi \tau }{\left(\frac{m_{B}V_{M}}{M_{B}S}\right)}^{2}{\left(\frac{\Delta E_{S}}{\Delta E_{t}}\right)}^{2}\left(t\ll \frac{L^{2}}{D}\right)$$

Whereby *E_s_* represents steady‐state voltage change, *E_t_* imposed pulse driven voltage change, *τ* pulse time, *V_m_* molar volume, *m_B_* for mass and *M_B_* for Molecular weight. GITT curves were obtained with repeated procedures as 20 mA g^−1^ of pulse imposed for 15 min followed by 1 hour of rest, until the voltage reaches 4.3 V and 3.0 V (vs Li^+^/Li). The overpotential was calculated from the difference between closed circuit potential (measured potential during pulse imposed) and quasi‐open circuit voltage (equilibrium potential without polarisation). All the electroanalyses were done by using a potentiostat (Biologic, VMP3 series) with quasi four point probes at 30 °C.

## Conflict of Interest

The authors declare no conflict of interest.

## Supporting information



Supporting Information

## Data Availability

The data that support the findings of this study are openly available in University of Liverpool Data Catalogue at https://datacat.liverpool.ac.uk/id/eprint/2836, reference number 2836.
