# Supplementary material for: High Rate Capability and Cycling Stability in Multi‐Domain Nanocomposite LiNi1– x Ti3 x /4O2 Positive Electrodes
Source: Adv Mater. 2025 Jul 22;37(39):2417899. doi: 10.1002/adma.202417899 (PMC12506615; doi:10.1002/adma.202417899)
Supplement: Supplementary file 1 — Supporting Information [file ADMA-37-2417899-s001.pdf]

ADVANCED MATERIALS

Supporting Information

for *Adv. Mater.*, DOI 10.1002/adma.202417899

High Rate Capability and Cycling Stability in Multi-Domain Nanocomposite $LiNi_{1-x}Ti_{3x/4}O_2$ Positive Electrodes

Jungwoo Lim, Manel Sonni, Luke M. Daniels, Mounib Bahri, Marco Zanella, Ruiyong Chen, Zhao Li, Alex R. Neale, Hongjun Niu, Nigel D. Browning, Matthew S. Dyer, John B. Claridge, Laurence J. Hardwick and Matthew J. Rosseinsky**

Supporting Information

for

High rate capability and cycling stability in multi-domain nanocomposite $LiNi_{1-x}Ti_{3x/4}O_2$ Positive Electrodes

Jungwoo Lim,[1,2,3,†] Manel Sonni,[1,3,†] Luke M. Daniels,[1] Mounib Bahri,[4] Marco Zanella,[1] Ruiyong Chen,[1] Zhao Li,[1,2,3] Alex R. Neale,[1,2] Hongjun Niu,[1] Nigel D. Browning,[4,5] Matthew S. Dyer,[1,6] John B. Claridge,[1,6] Laurence J. Hardwick, [1,2,6,*] Matthew J. Rosseinsky[1,6,*]

[†]These authors contributed equally. Author names are listed alphabetically.

*Corresponding authors hardwick@liverpool.ac.uk, M.J.Rosseinsky@liverpool.ac.uk

[1]Department of Chemistry, University of Liverpool, Crown Street, Liverpool L69 7ZD, United Kingdom.

[2]Stephenson Institute for Renewable Energy, Department of Chemistry, University of Liverpool, Liverpool L69 7ZF, United Kingdom.

[3]The Faraday Institution, Didcot OX11 0RA, U.K.

[4]Albert Crewe Centre, University of Liverpool, Research Technology Building, Elisabeth Street, Pembroke Place, Liverpool L69 3GE, United Kingdom.

[5]School of Engineering, Department of Mechanical, Materials and Aerospace Engineering, University of Liverpool, Liverpool L69 3GH, United Kingdom.

[6]Leverhulme Research Centre for Functional Materials Design, Materials Innovation Factory, Oxford Street, Liverpool L7 3NY, United Kingdom.

1. Literature review

Table S1. Previously reported studies of Ti substitution into $LiNiO_2$ rock salt. Both the Li content and total transition metal content are constrained to unity through $LiNi_{1-x}Ti_xO_2$, which is distinct to the substitution strategy employed in this paper ($LiNi_{1-x}Ti_{3x/4}O_2$), where a Ni^{3+} oxidation state is retained through the simultaneous introduction of Ti^{4+} and cation vacancies.

**Structures/Compositions**	**Ref.**
$Fm\bar{3}m$ phase of $Li_{1+z/3}Ni_{1/2-z/2}Ti_{1/2+z/6}O_2$ ($0 \le z \le 0.5$)	[1]
$R\bar{3}m$ phases of $LiNi_{1-y}Ti_yO_2$ (y = 0.02, 0.05, 0.10, and 0.15)	[2]
$R\bar{3}m$ phases of $LiNi_{1-x}Ti_xO_2$ ($0.025 \le x \le 0.2$)	[3]
$R\bar{3}m$ phases of $LiNi_{0.9}Ti_{0.1}O_2$	[4]
$R\bar{3}m$ phases of $Li_xNi_{1-y}Ti_yO_2$ ($0.1 \le y \le 0.3$)	[5]
$R\bar{3}m$ phases of $Li_{1+x}Ni_{1-x}Ti_xO_{2+x}$ ($0.05 \le x \le 0.4$) and $Fm\bar{3}m$ for x=0.5	[6]
$R\bar{3}m$ phases $LiNi_{1-x}Ti_xO_2$ (x = 0, 0.025, 0.05, 0.1)	[7]
$R\bar{3}m$ phase $LiNi_{0.99}Ti_{0.01}O_2$	[8]
$R\bar{3}m$ phase $LiNi_{1-x}Ti_xO_2$ (x = 0, 0.001, 0.002 and 0.004)	[9]
$R\bar{3}m$ phases of $LiNi_{1-z}M_zO_2$ (M = Co, Mn, Ti, z = 0.1, 0.2, 0.3)	[10]
$R\bar{3}m$ phase of $LiNi_{0.9925}Ti_{0.0075}O_2$	[11]

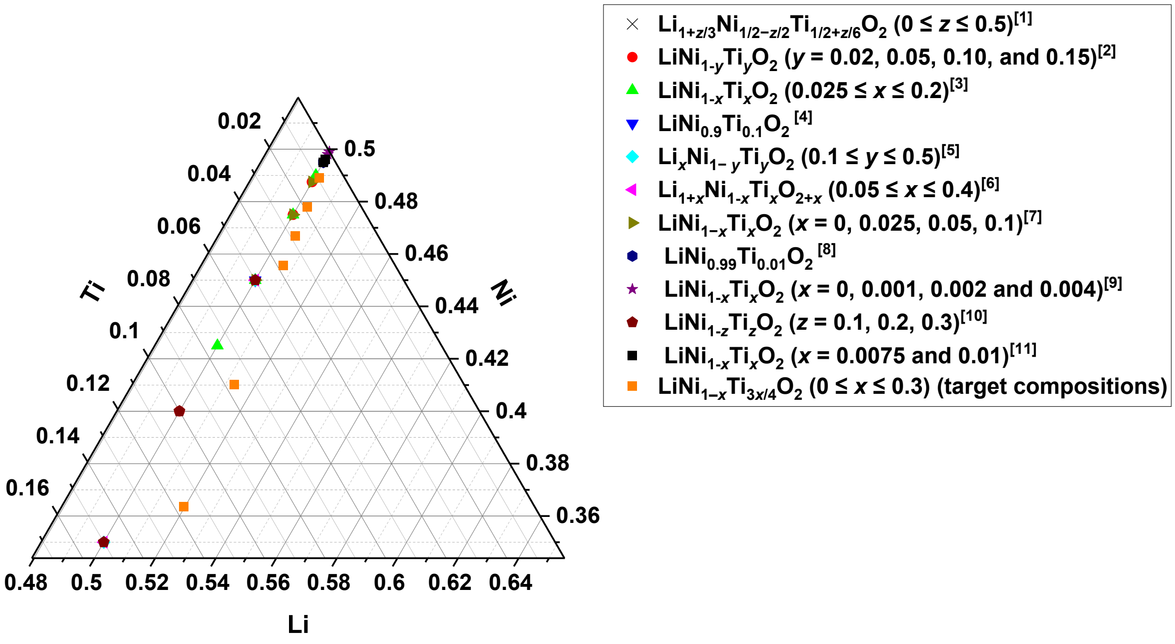

Figure S1. Ternary plot showing the previously reported $LiNi_{1-x}Ti_xO_2$ materials and the targeted $LiNi_{1-x}Ti_{3x/4}O_2$ ($0 \le x \le 0.3$) compositions in this work (orange stars).

2. LiOH precursor preparation

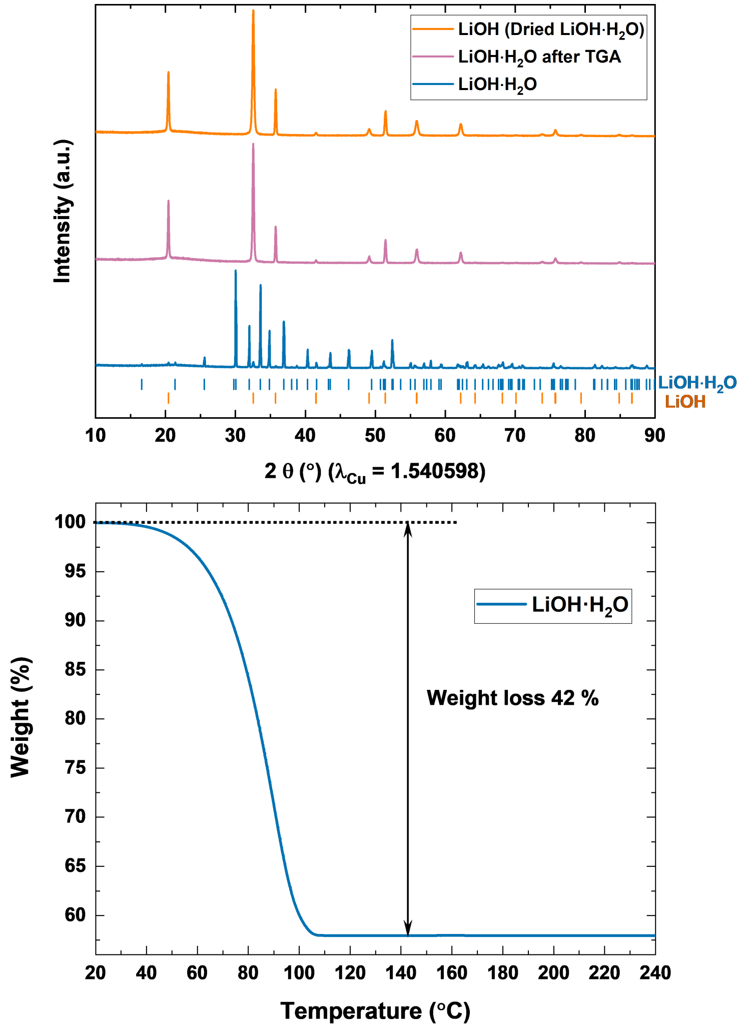

Figure S2: (a) X-ray diffraction pattern of as-purchased LiOH·H_2O (blue), LiOH·H_2O following thermogravimetric analysis (TGA) up to 240 °C (purple), and LiOH dried under dynamic vacuum at 200 °C (orange). **(b)** TGA plot of LiOH·H_2O up to 240 °C (expected weight loss is 42.92%).

A solution for ICP-MS was produced by dissolving 14 mg of the vacuum-dried LiOH in ultra-pure water. The results of the measurements are summarized in **Table S2**.

Table S2. ICP-MS analysis of dried LiOH.

Expected Li concentration	Measured Li concentration
4.06 ppm	4.037(22) ppm

3. SPXRD of $LiNi_{1-x}Ti_{3x/4}O_2$ ($0 \leq x \leq 0.3$)

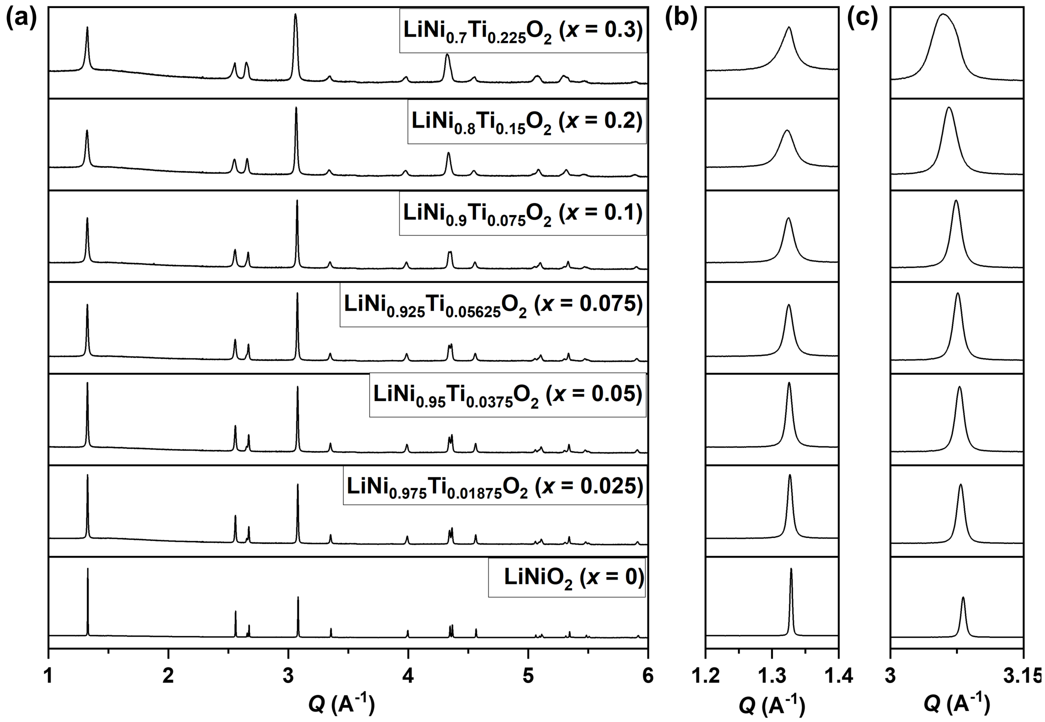

Figure S3. (a) Synchrotron powder XRD patterns (λ = 0.825005(1) Å) of $LiNi_{1-x}Ti_{3x/4}O_2$ ($0 \leq x \leq 0.3$), **(b)** demonstrating the observed broadening of the $(003)_H$ peak with increasing x, and **(c)** the observed splitting of the $(104)_H$ peak at $x = 0.3$.

4. PXRD of $LiNi_{1-x}Ti_xO_2$ ($0 \leq x \leq 0.2$)

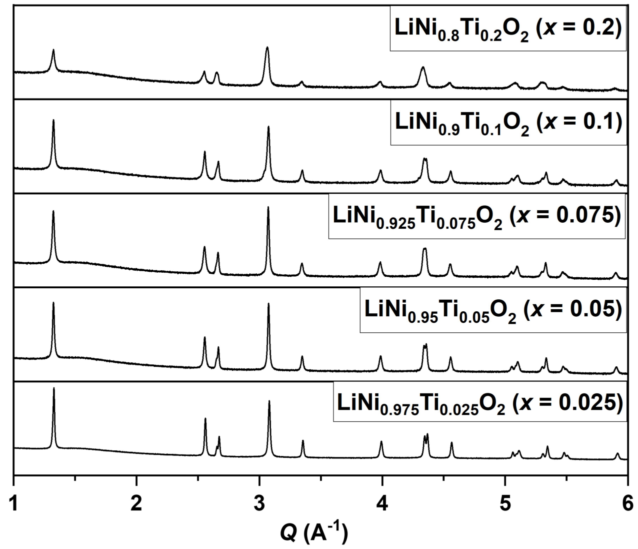

Figure S4. Powder XRD patterns (λ = 1.541874 Å) of stoichiometric $LiNi_{1-x}Ti_xO_2$ powders ($0.025 \leq x \leq 0.2$).

5. Compositional analysis of $LiNi_{1-x}Ti_{3x/4}O_2$ and $LiNi_{1-x}Ti_xO_2$ ($0 \leq x \leq 0.1$)

The synthesis of the nominally Ni^{3+} containing $LiNi_{1-x}Ti_{3x/4}O_2$ compositions reported here used stochiometric amounts of LiOH (dried), $Ni(OH)_2$ and TiO_2. The starting materials and as-made powders were always handled under inert atmosphere, including transfers to instruments for measurement. IR measurements (**Figure S5**) confirms that no LiOH or Li_2CO_3 are observed which rules out any surface-based Li impurities that are frequently encountered when dealing with Ni-rich materials.

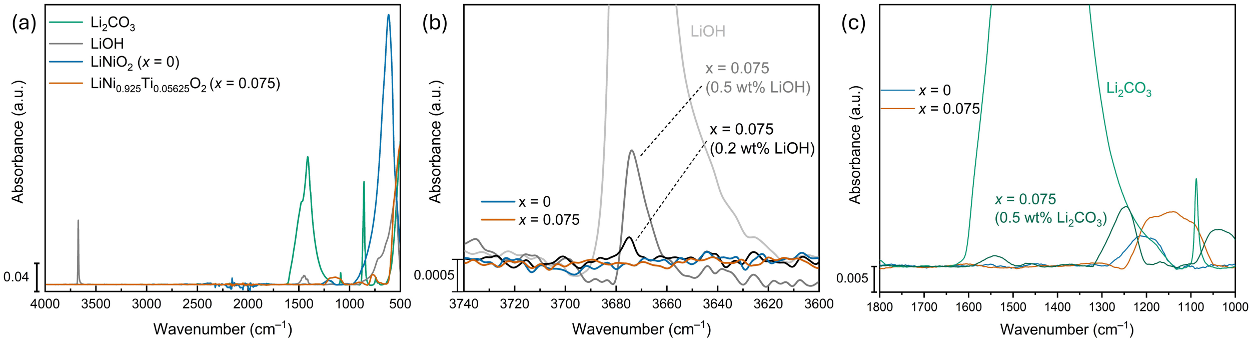

Figure S5. (a) Infrared (IR) spectra of pristine $LiNiO_2$, $LiNi_{0.925}Ti_{0.05625}O_2$, LiOH and Li_2CO_3 indicating no contamination of the as-made powders from hydroxide (OH^-) or carbonate (CO_3^{2-}) species. **(b)** Enlarged spectra in the region of the strong absorption corresponding to the O–H stretch of LiOH (3740-3600 cm^{-1}). **(c)** Enlarged spectra in the region of the strong absorption associated with CO_3^{2-} stretches in Li_2CO_3 (1800-1000 cm^{-1}).

ICP-MS data were collected on triplicate solutions for each composition and confirm the nominal Ni and Ti contents alongside TEM-EDX data. The errors obtained on Li content encompass both vacancy-containing and vacancy-free compositions, and represent the highest measurement accuracy possible.

Table S3. Compositional analysis of $LiNi_{1-x}Ti_{3x/4}O_2$ and stoichiometric $LiNi_{1-x}Ti_xO_2$ ($0 \leq x \leq 0.2$) obtained by ICP-MS and TEM-EDX.

Target composition		ICP-MS	TEM-EDX (Ti/Ni)	
			**Measured**	**Expected**
$LiNiO_2$ (x =0)		$Li_{1.019(20)}Ni_{1.000(27)}O_2$	-	-
$LiNi_{1-x}Ti_{3x/4}O_2$	$LiNi_{0.975}Ti_{0.01875}O_2$ (x =0.025)	$Li_{1.010(21)}Ni_{0.975(22)}Ti_{0.0184(4)}O_2$	0.022(5)	0.019
	$LiNi_{0.95}Ti_{0.0375}O_2$ (x =0.05)	$Li_{1.023(17)}Ni_{0.950(17)}Ti_{0.0373(5)}O_2$	0.043(9)	0.039
	$LiNi_{0.925}Ti_{0.05625}O_2$ (x =0.075)	$Li_{1.027(20)}Ni_{0.925(11)}Ti_{0.0553(8)}O_2$	0.049(7)	0.060
	$LiNi_{0.9}Ti_{0.075}O_2$ (x =0.1)	$Li_{1.024(14)}Ni_{0.900(12)}Ti_{0.079(2)}O_2$	0.092(9)	0.083
	$LiNi_{0.8}Ti_{0.15}O_2$ (x =0.2)	$Li_{1.056(9)}Ni_{0.800(4)}Ti_{0.144(1)}O_2$	-	-
$LiNi_{1-x}Ti_xO_2$	$LiNi_{0.975}Ti_{0.025}O_2$ (x = 0.025)	$Li_{1.084(22)}Ni_{0.975(21)}Ti_{0.0230(5)}O_2$	-	-
	$LiNi_{0.95}Ti_{0.05}O_2$ (x = 0.05)	$Li_{1.035(13)}Ni_{0.950(11)}Ti_{0.0473(4)}O_2$	-	-
	$LiNi_{0.925}Ti_{0.075}O_2$ (x = 0.075)	$Li_{1.027(22)}Ni_{0.925(18)}Ti_{0.0703(2)}O_2$	-	-
	$LiNi_{0.9}Ti_{0.1}O_2$ (x = 0.1)	$Li_{1.022(15)}Ni_{0.900(23)}Ti_{0.102(3)}O_2$	-	-
	$LiNi_{0.8}Ti_{0.2}O_2$ (x = 0.2)	$Li_{1.032(9)}Ni_{0.800(5)}Ti_{0.197(2)}O_2$	-	-

Magnetisation data were collected for the nominally Ni^{3+} compositions, $LiNiO_2$ and vacancy-containing $LiNi_{0.9}Ti_{0.075}O_2$, and were compared with an additional vacancy-free sample of composition $LiNi_{0.9}Ti_{0.1}O_2$, in which the substitution of Ti^{4+} introduces an equivalent amount of Ni^{2+}. **Figure S6a** shows the Zero-Field cooled (ZFC) and field-cooled (FC) Magnetic susceptibility data collected at 100 Oe from which the magnetic freezing temperature (T_f) is determined from the observed maxima. A temperature of 14 K is extracted for $LiNiO_2$ (**Figure S6a**), and alongside the inverse susceptibility high-field (45 kOe) data which show linear Curie-Weiss behavior (**Figure S6b**), this confirms the as-made sample of $LiNiO_2$ has a stoichiometry that matches that of the nominal.[12-15] A T_f of 14 K is also extracted for $LiNi_{0.9}Ti_{0.075}O_2$, identical to $LiNiO_2$ (**Figure S6a**), and this composition also displays linear Curie-Weiss behaviour in high-field measurements (**Figure S6b**). The Curie-Weiss fitting parameters (**Table S4**) and extracted effective moments of 1.731(2) and 1.7966(1) for $LiNiO_2$ and $LiNi_{0.9}Ti_{0.075}O_2$, respectively, are highly comparable and close to the S = ½ value expected (1.73 μ_B) for Ni^{3+}. We contrast this against a sample synthesised with nominal composition of $LiNi_{0.9}Ti_{0.1}O_2$, which displays significant deviation from linear behavior observed in the inverse susceptibility data and an increased freezing temperature of 22 K. Such observations are known to be consistent with the introduction of Ni^{2+}.[12-15]

These measurements provide direct evidence that the electronic structure of the formally Ni^{3+} containing compositions $LiNiO_2$ and vacancy-containing $LiNi_{0.9}Ti_{0.075}O_2$ are very similar, whereas the vacancy-free composition of $LiNi_{0.9}Ti_{0.1}O_2$ displays significantly different behavior. These data thus support the vacancy-containing Rietveld model which is consistent with both the diffraction data and the nominal composition for the $LiNi_{1-x}Ti_{3x/4}O_2$ materials.

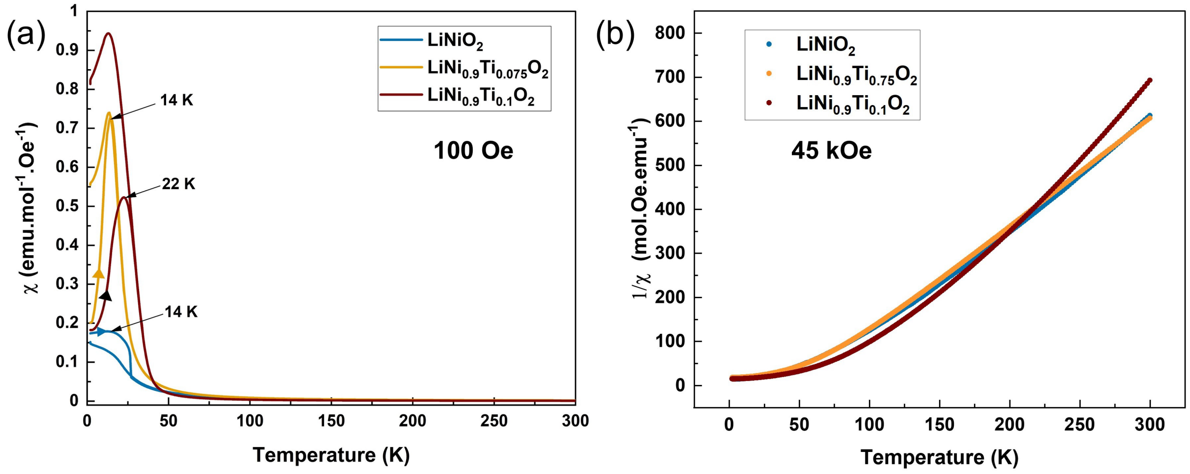

Figure S6. (a) Zero-Field cooled (highlighted by an arrow on the data) and field-cooled magnetic susceptibility data collected under an applied field of 100 Oe for nominally Ni^{3+} $LiNiO_2$ and $LiNi_{0.9}Ti_{0.075}O_2$ compositions, and the vacancy-free composition of $LiNi_{0.9}Ti_{0.1}O_2$. The magnetic freezing temperature (T_f) shown for each composition is determined from the maxima of the ZFC curves. **(b)** Field-cooled reciprocal magnetic susceptibility collected under an applied field of 45 kOe for $LiNiO_2$, $LiNi_{0.9}Ti_{0.075}O_2$ and $LiNi_{0.9}Ti_{0.1}O_2$.

Table S4: Parameters extracted from magnetisation data of $LiNiO_2$ and vacancy-containing $LiNi_{0.9}Ti_{0.075}O_2$. The Weiss temperature (θ_w), Curie constant (C), and effective magnetic moment (μ_{eff}) were determined by fitting the inverse susceptibility data at high temperatures (200-300 K). The magnetic freezing temperature (T_f) is determined from the maxima observed in ZFC curves shown in **Figure S6a**. The similar values obtained here are consistent with the vacancy-containing models obtained from diffraction and the nominal $LiNi_{1-x}Ti_{3x/4}O_2$ compositions.

	T_f (K)	C	μ_{eff} (μ_B)	θ_w (K)
$LiNiO_2$	14	0.377(1)	1.731(2)	69.95(69)
$LiNi_{0.9}Ti_{0.075}O_2$	14	0.4059(1)	1.7966(1)	53.45(4)

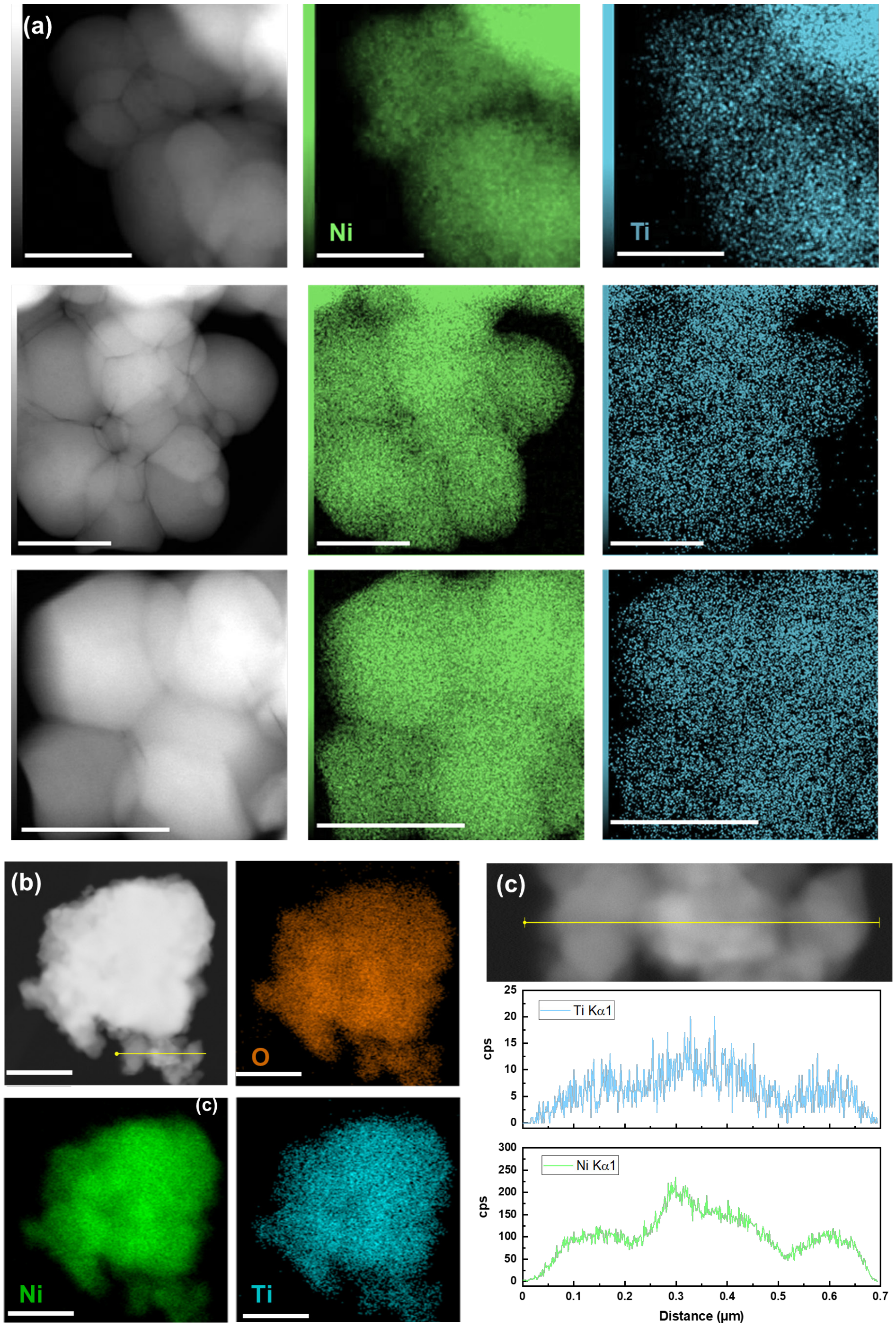

Figure S7. STEM image showing particles of x = 0.075 ($LiNi_{0.925}Ti_{0.05625}O_2$). EDX mapping for O (orange), Ni (green) and Ti (blue) collected at 20 keV (scale bar: **(a)** 500 nm **(b)** 100 nm). **(c)** EDX elemental line scan profile collected at 20 keV confirming homogeneous distribution of Ti and Ni throughout the bulk of the particles. Additional STEM images and EDX maps collected on a statistically significant number of particles further confirm the homogeneous distributions.

6. Structural analysis and structural models for $LiNi_{1-x}Ti_{3x/4}O_2$ (x = 0, 0.05 and 0.1)

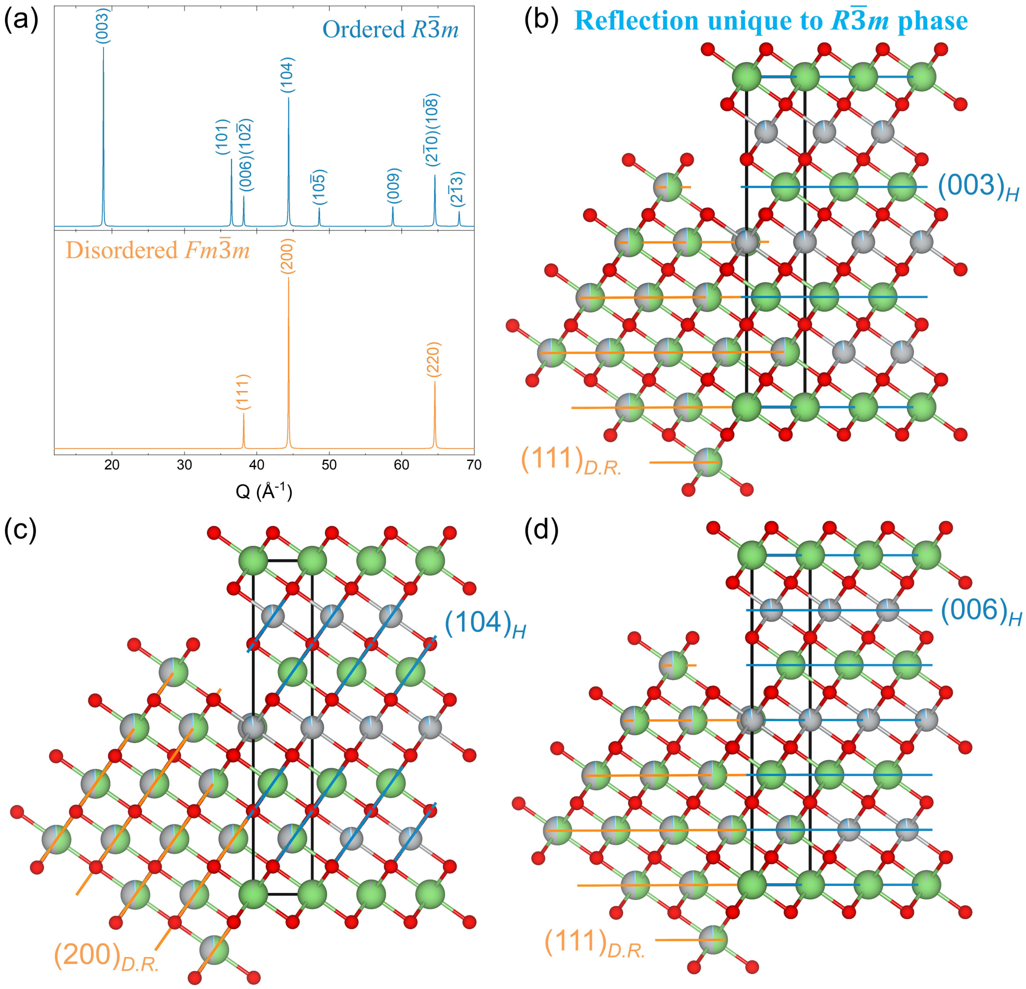

Figure S8. (a) Simulated PXRD patterns for ordered $R\bar{3}m$ (top) and disordered $Fm\bar{3}m$ (bottom) $LiNiO_2$ structures with indexed reflections. In the absence of a distortion from cubic symmetry, the two sub-cells perfectly relate to one another with exactly overlapping lattices. **(b)** Some reflections are unique to the ordered hexagonal $R\bar{3}m$ supercell, for example $(003)_H$. Examples of the relationship between the ordered hexagonal $R\bar{3}m$ and disordered cubic $Fm\bar{3}m$ structures are provided in **(c)** and **(d)** which demonstrate Bragg reflections that are common to both structures. (c) shows the equivalency of the $(200)_{D.R.}$ and $(104)_H$ reflections and (d) the $(111)_{D.R.}$ and $(006)_H$ reflections. Bragg reflections with Miller indices that have even values of $l(hkl_{\text{even}})$) arise from cubic $Fm\bar{3}m$ cation disordered domains, while Bragg reflections which are forbidden in the cubic unit cell that exhibit odd values of $l(hkl_{\text{odd}})$ arise from domains of the cation-ordered $R\bar{3}m$ structure.

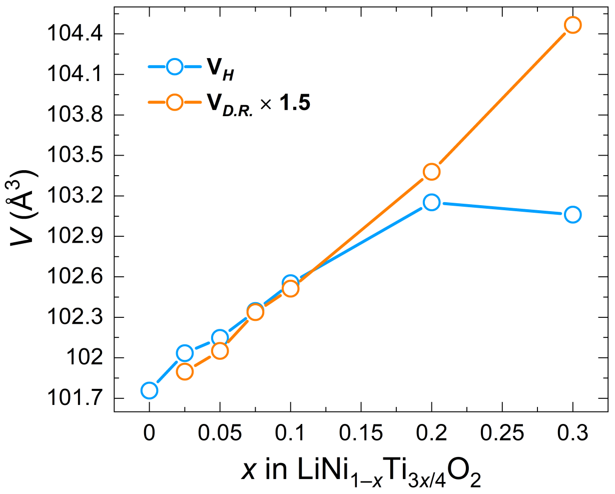

Figure S9. Trend in unit cell volumes of ordered $R\bar{3}m$ (*H*) and the disordered (*D.R.*) $Fm\bar{3}m$ structures when fitting SPXRD data (λ = 0.825005(1) Å) with a model containing two separate phases and a fixed overall composition. The observed divergence in unit cell volume for $x > 0.2$ indicates compositional inhomogeneity and likely segregation of cations and vacancies between the two phases. The compositions $0 < x < 0.2$ are described by a multidomain single-phase hexagonal $R\bar{3}m$ model described in the text.

Table S5. Unit cell parameters, volume and hexagonal distortion parameter (σ) obtained by refinement of a multidomain single-phase hexagonal $R\bar{3}m$ model against synchrotron powder XRD data (λ = 0.825005(1) Å) for of $LiNi_{1-x}Ti_{3x/4}O_2$ ($0 \leq x \leq 0.2$). The hexagonal c lattice parameter is calculated from $c = a\,\sigma\sqrt{24}$.

Composition	*a* (Å)	*c* (Å)	*V* (Å³)	*σ*
$LiNiO_2$ (x =0)	2.87749(1)	14.1933(1)	101.776(2)	1.00685(1)
$LiNi_{0.975}Ti_{0.01875}O_2$ (x =0.025)	2.87985(3)	14.2097(2)	102.059(4)	1.00718(1)
$LiNi_{0.95}Ti_{0.0375}O_2$ (x =0.05)	2.88047(4)	14.2182(3)	102.165(5)	1.00758(1)
$LiNi_{0.925}Ti_{0.05625}O_2$ (x =0.075)	2.88259(6)	14.2233(4)	102.352(6)	1.00719(2)
$LiNi_{0.9}Ti_{0.075}O_2$ (x =0.1)	2.88442(2)	14.2264(3)	102.505(3)	1.00677(2)
$LiNi_{0.8}Ti_{0.15}O_2$ (x =0.2)	2.89165(10)	14.2503(7)	103.212(11)	1.00614(3)

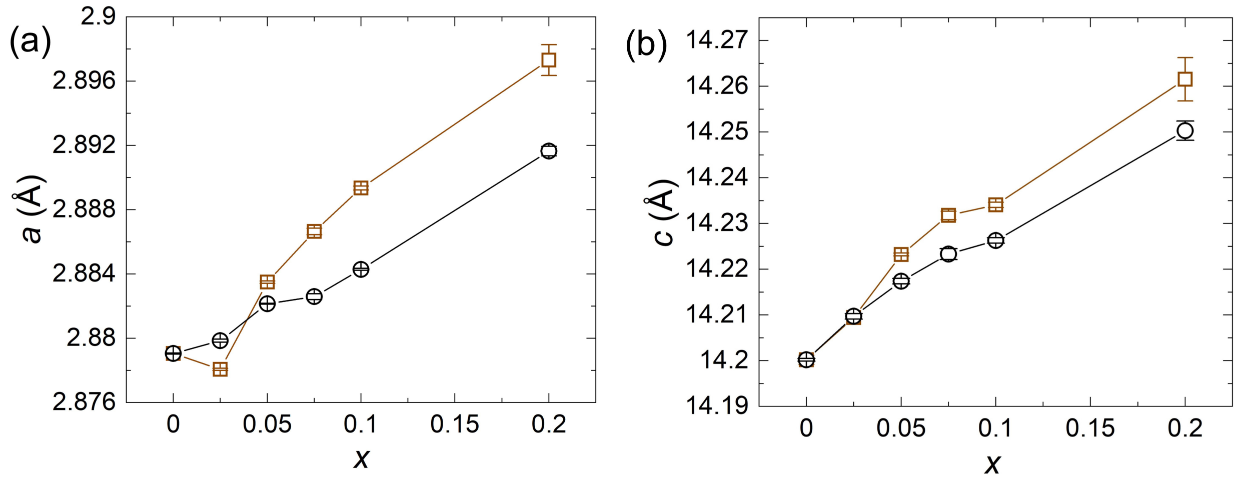

Figure S10. Trend in **(a)** hexagonal *a* lattice parameter and **(b)** hexagonal *c* lattice parameter (error bars are multiplied by 3) for $LiNi_{1-x}Ti_{3x/4}O_2$ and $LiNi_{1-x}Ti_xO_2$ ($0 \leq x \leq 0.2$) obtained by structural refinement of the multidomain single-phase hexagonal $R\overline{3}m$ model described in the main text. The hexagonal *c* lattice parameter is calculated from the hexagonal *a* lattice parameter and hexagonal distortion parameter (σ) through $c = a\ \sigma\sqrt{24}$. Unit cell parameters were extracted for the stoichiometric $LiNi_{1-x}Ti_xO_2$ ($0 \leq x \leq 0.2$) compositions via the internal standard method using NIST SRM 660c LaB_6 powder.

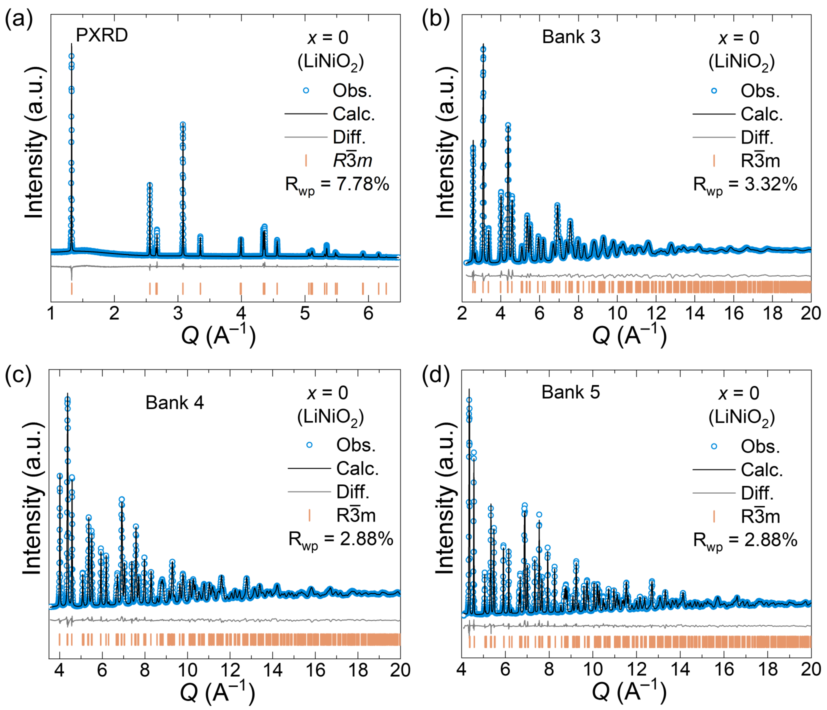

Figure S11. Combined Rietveld refinement of $^7LiNiO_2$ against **(a)** powder XRD and time-of-flight neutron powder diffraction data collected from **(b)** Bank 3 (2θ = 67°), **(c)** Bank 4 (2θ = 122°), and **(d)** Bank 5 (2θ = 154°) of NOMAD at room temperature using the multidomain single-phase hexagonal $R\bar{3}m$ model described in the main text. Traces I_{obs} (blue circles), I_{calc} (black line), $I_{obs}-I_{calc}$ (grey line), and Bragg reflections (orange tick marks) are shown. The combined refinement including XRD data and NPD data across three detector banks has an R_{wp} of 3.08%, R_{exp} of 0.38%, and GoF of 8.06.

Table S6. Fractional atomic coordinates and isotropic displacement parameters of $^7LiNiO_2$. The combined refinement including XRD data and NPD data across four detector banks has an R_{wp} of 3.08%, R_{exp} of 0.38%, and GoF of 8.06.

$\boldsymbol{R\bar{3}m}$: a = 2.879048(14) Å, c = 14.20017(13) Å, V =100.935(2) Å^3						
**Atom**	**Wyck.**	**S.O.F.**	***x***	***y***	***z***	**B_{iso} (Å^2)**
Li1	3*b*	0.9764(5)	0.00000	0.00000	0.00000	0.83(2)
Ni1	3*b*	0.0236(5)	0.00000	0.00000	0.00000	0.83(2)
Li2	3*a*	0.0236(5)	0.00000	0.00000	0.50000	0.241(2)
Ni2	3*a*	0.9764(5)	0.00000	0.00000	0.50000	0.241(2)
O	6*c*	1	0.00000	0.00000	0.24119(2)	0.757(5)

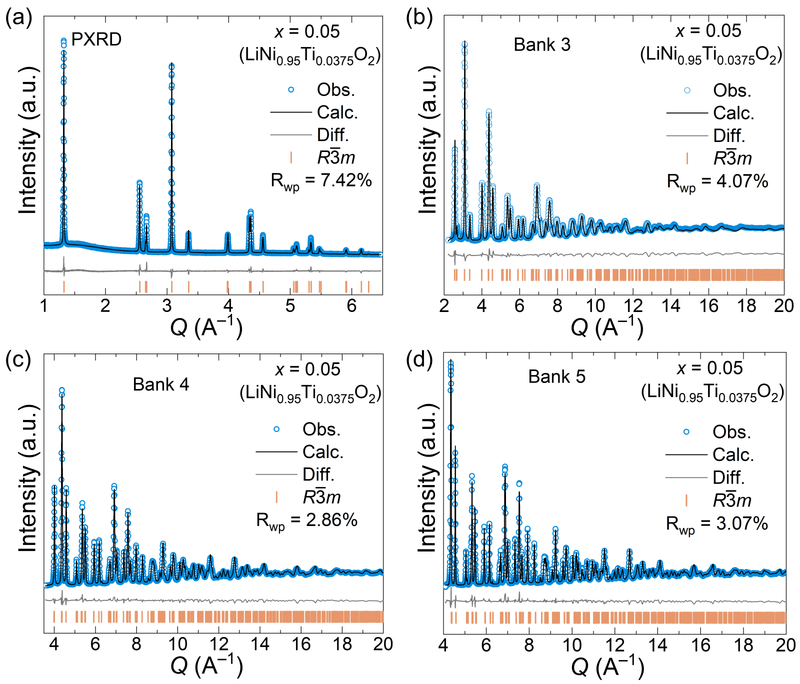

Figure S12. Combined Rietveld refinement of $^{7}LiNi_{0.95}Ti_{0.0375}O_2$ (x=0.05) against **(a)** powder XRD and time-of-flight neutron powder diffraction data collected from **(b)** Bank 3 (2θ = 67°), **(c)** Bank 4 (2θ = 122°), **(d)** Bank 5 (2θ = 154°) of NOMAD at room temperature using the multidomain single-phase hexagonal $R\bar{3}m$ model described in the main text. Traces I_{obs} (blue circles), I_{calc} (black line), I_{obs}–I_{calc} (grey line), and Bragg reflections (orange tick marks) are shown. The combined refinement including XRD data and NPD data across four detector banks has an R_{wp} of 3.2996%, R_{exp} of 0.4%, and GoF of 8.44.

Table S7. Fractional atomic coordinates, site occupancy factors (S.O.F.), and isotropic displacement parameters from simultaneous Rietveld refinement against PXRD and NPD (three detector banks) data collected from a powder sample of $^{7}LiNi_{0.95}Ti_{0.0375}O_2$ (x = 0.05). The combined refinement has an R_{wp} of 3.2996%, R_{exp} of 0.4%, and GoF of 8.44. The total content of Li, Ni and Ti across the 3a and 3b positions was refined under a global composition restraint which incorporated the information from ICP-MS measurement. The total Li content was set to 1.02, the total Ni content was set to 0.95 and the total Ti content was set to 0.03, each with penalties of 10^{10}.

R*3̄*m : a = 2.882147(14) Å, c = 14.21744(18) Å, V = 102.2785(19) Å^3						
**Atom**	**Wyck.**	**S.O.F.**	x	y	z	B_{iso} (Å^2)
Li1	3a	0.956(11)	0	0	0	1.08(3)
Ni1	3a	0.039(1)	0	0	0	1.08(3)
Ti1	3a	0.014(2)	0	0	0	1.08(3)
Li2	3b	0.067(7)	0	0	0.5	0.172(1)
Ni2	3b	0.911(1)	0	0	0.5	0.172(1)
Ti2	3b	0.023(4)	0	0	0.5	0.172(1)
O	6c	1	0	0	0.24144(2)	0.680(5)

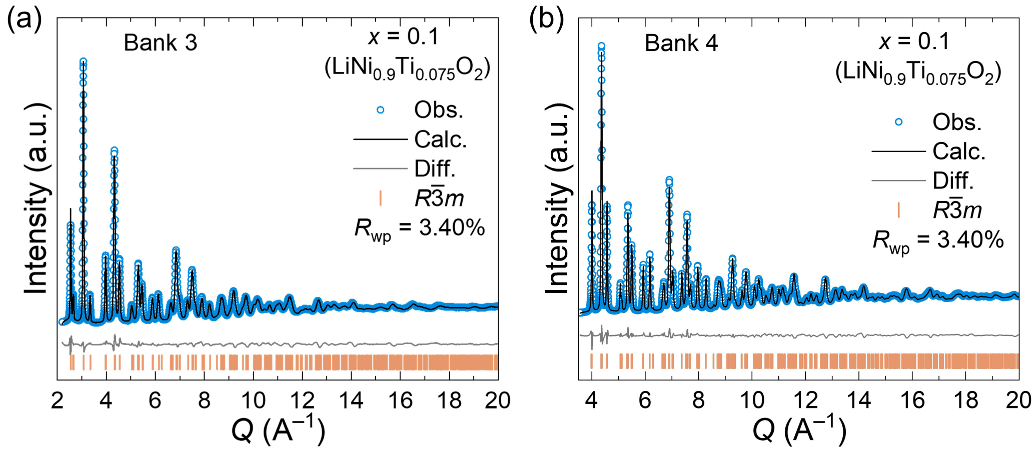

Figure S13. Combined Rietveld refinement of $^{7}LiNi_{0.9}Ti_{0.075}O_2$ (x=0.1) against **(a)** Bank 3 (2θ = 67°) and **(b)** Bank 4 (2θ = 122°) of NOMAD at room temperature using the multidomain single-phase hexagonal $R\bar{3}m$ model described in the main text. Traces I_{obs} (blue circles), I_{calc} (black line), $I_{obs}-I_{calc}$ (grey line), and Bragg reflections (orange tick marks) are shown. The combined refinement including PXRD data and NPD data across three detector banks has an R_{wp} of 3.2267%, R_{exp} of 0.4%, and GoF of 7.94.

Table S8. Fractional atomic coordinates, site occupancy factors (S.O.F.), and isotropic displacement parameters from simultaneous Rietveld refinement against PXRD and NPD (four detector banks) data collected from a powder sample of $^{7}LiNi_{0.9}Ti_{0.075}O_2$ (x = 0.1). The combined refinement has an R_{wp} of 3.2267%, R_{exp} of 0.4%, and GoF of 7.94. The total content of Li, Ni and Ti across the 3a and 3b positions was refined under a global composition restraint which incorporated the information from ICP-MS measurement. The total Li content was set to 1.02, the total Ni content was set to 0.9 and the total Ti content was set to 0.08, each with penalties of 10^7.

$\boldsymbol{R\bar{3}m}$: a = 2.884293(17) Å, c = 14.2263(2) Å, V = 102.495(2) Å^3						
**Atom**	**Wyck.**	**S.O.F.**	$\boldsymbol{x}$	$\boldsymbol{y}$	$\boldsymbol{z}$	$\boldsymbol{B_{iso}}$ (Å^2)
Li1	3a	0.917(9)	0	0	0	1.09(4)
Ni1	3a	0.056(1)	0	0	0	1.09(4)
Ti1	3a	0.028(2)	0	0	0	1.09(4)
Li2	3b	0.106(6)	0	0	0.5	0.194(2)
Ni2	3b	0.845(1)	0	0	0.5	0.194(2)
Ti2	3b	0.050(3)	0	0	0.5	0.194(2)
O	6c	1	0	0	0.24180(2)	0.640(5)

7. Scanning Transmission Electron Microscopy (STEM) and Continuous Rotation Electron Diffraction (CRED) data for pristine $LiNiO_2$ (x = 0), $LiNi_{0.925}Ti_{0.05625}O_2$ (x = 0.075) and $LiNi_{0.9}Ti_{0.075}O_2$ (x = 0.1)

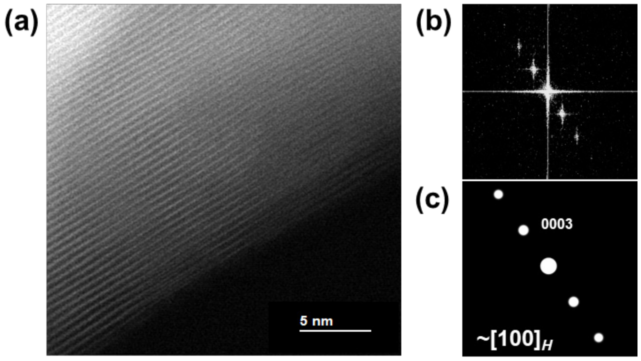

Figure S14. (a) STEM-HAADF image for x = 0 ($LiNiO_2$) aligned along the (a) ~$[100]_H$ direction. **(b)** The corresponding experimental FFT pattern is compared against **(c)** patterns simulated from the refined $R\bar{3}m$ structure for $LiNiO_2$ reported above.

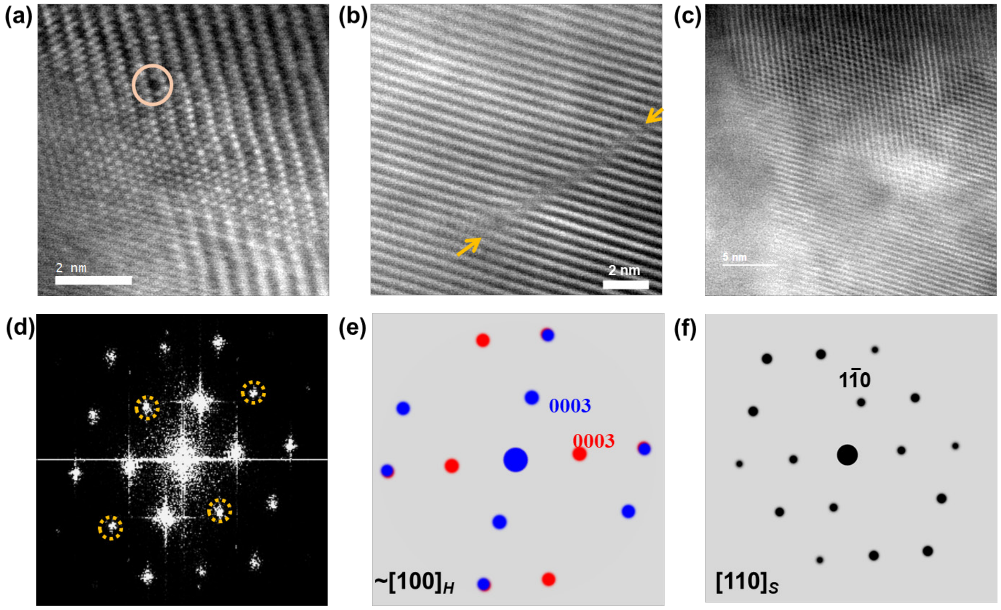

Figure S15. HAADF-STEM images for x = 0.075 ($LiNi_{0.925}Ti_{0.05625}O_2$) showing **(a)** vacancy/defect (marked with pink circle), **(b)** dislocation array (marked with yellow arrows), **(c)** ordered-ordered intergrowth with **(d)** corresponding FFT and **(e)** simulated patterns of two ordered structures oriented along ~$[100]_H$ separated by an angle of ~70°. Contributions of reflections from two ordered domains give rise to weak multiple diffraction (marked with orange circles in d), which could be mistaken for reflections associated with a spinel unit cell (e.g. see simulated pattern for spinel along $[110]_S$ in **(f)**).

The FFT pattern of two overlapping ordered rock salt domains (Figure **S54. d.**) can be confused with the spinel structure (Figure **S15. f.**) which produces a similar diffraction pattern. To confirm the absence of a spinel phase, CRED analysis has been performed on a single particle of $LiNi_{0.925}Ti_{0.05625}O_2$ (x = 0.075). On attempting to fit the data with the spinel unit cell (a = 8.4 Å), three consecutive missing reflections were found in the 0kl and h0l planes which excludes the spinel cell from the analysis (**Figure S16a. and b**). Fitting the data with the $Fm\bar{3}m$ cell (a = 4.2 Å) the systematic absences assume a well-known pattern. The k, l absences in the 0kl plane (panel c), the h+l absences in the hhl plane (panel d) and the l absences in the 00l axes (both panel c and d) show that the DR cell can be indexed with a face centred (F) space group with an extinction symbol *F*– – –. Further data analysis using ShelXT[16] shows that the space group for the cubic cell is $Fm\bar{3}m$ and the refinement done with Olex2[17], forcing the composition to be $LiNi_{0.925}Ti_{0.05625}O_2$, confirms the results of PXRD and NPD with an R_{int} = 26.16% and a completeness of 100%.

As shown in **Figure 1g**, each crystallite contains both $R\bar{3}m$ and $Fm\bar{3}m$ phases. Multi-crystal analysis of CRED data performed using Crysalis pro, reveals an interesting multiphase intergrowth mechanism between the structures. A 3D distribution of seven $R\bar{3}m$ unit cells overlapped with a single $Fm\bar{3}m$ cell oriented along the [001] zone axes is shown in **Figure S17a**. Some of the found $R\bar{3}m$ unit cell shared the same c axes and are grouped together in **Figure S17b-e** for simplicity. The finding shows that all the $R\bar{3}m$ unit cells have the c axes perpendicular to the family of (111) planes of the $Fm\bar{3}m$ unit cell and as such there are specific angles between the different unit cells (**Figure S18**). These angles, measured from CRED data, agree very well with those extracted from FFTs of STEM images; ~70° between $R\bar{3}m$ and $R\bar{3}m$ (**Figure S15**), and ~55° between $R\bar{3}m$ and $Fm\bar{3}m$ unit cells (**Figure 1g**).

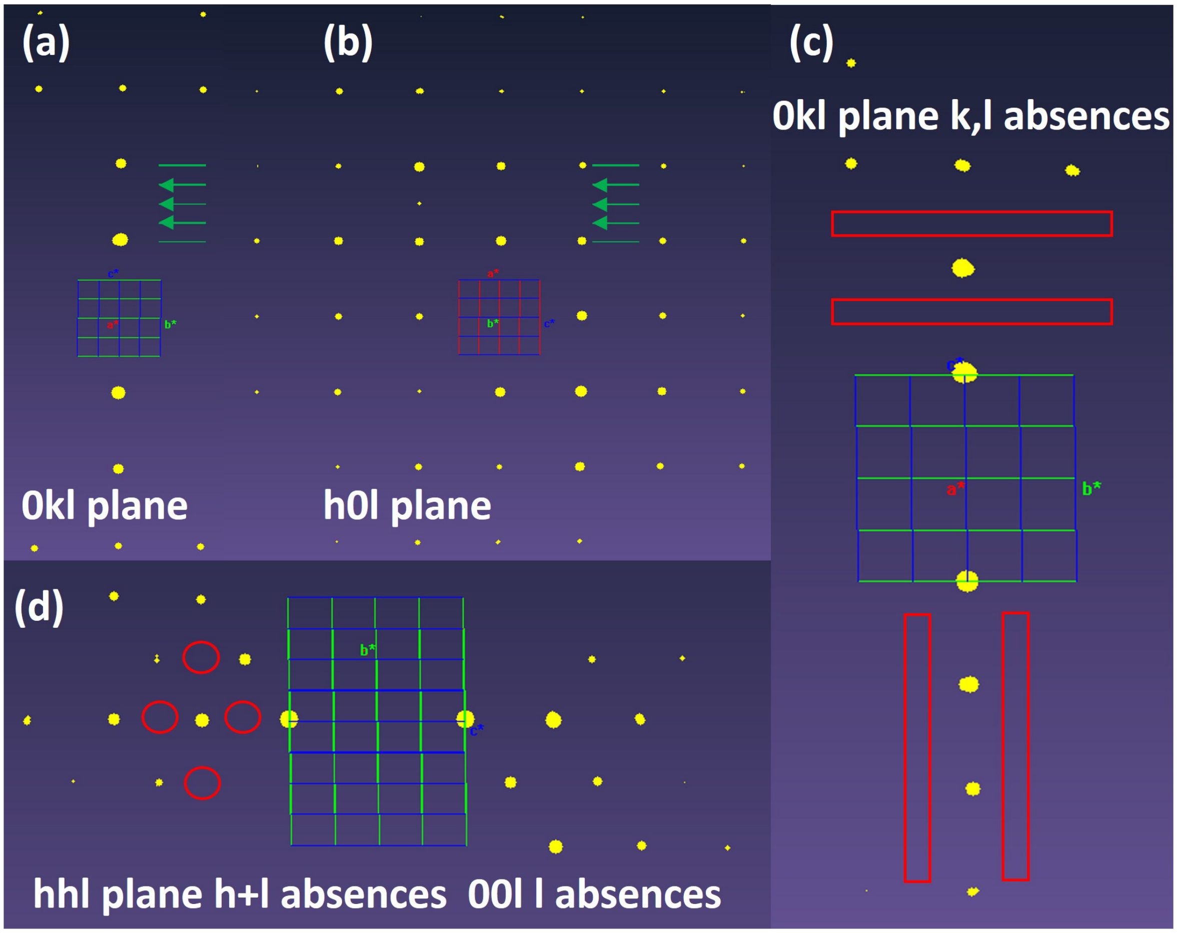

Figure S16. Comparison between the CRED reflections on the 0kl, h0l and hhl planes fitted with the 8.4 × 8.4 × 8.4 Å spinel (panels a and b) and the 4.2 × 4.2 × 4.2 Å *Fm*$\bar{3}$*m* cell (panel c and d) for $LiNi_{0.925}Ti_{0.05625}O_2$ ($x = 0.075$). The three consecutive missing reflections found in a) and b) exclude the spinel cell from the analysis. The k, l absences in the 0kl plane (panel c), the h+l absences in the hhl plane (panel d) and the l absences in the 00l axes (both panel c and d) show that the *Fm*$\bar{3}$*m* unit cell has a space group with a *F*– – – extinction symbol.

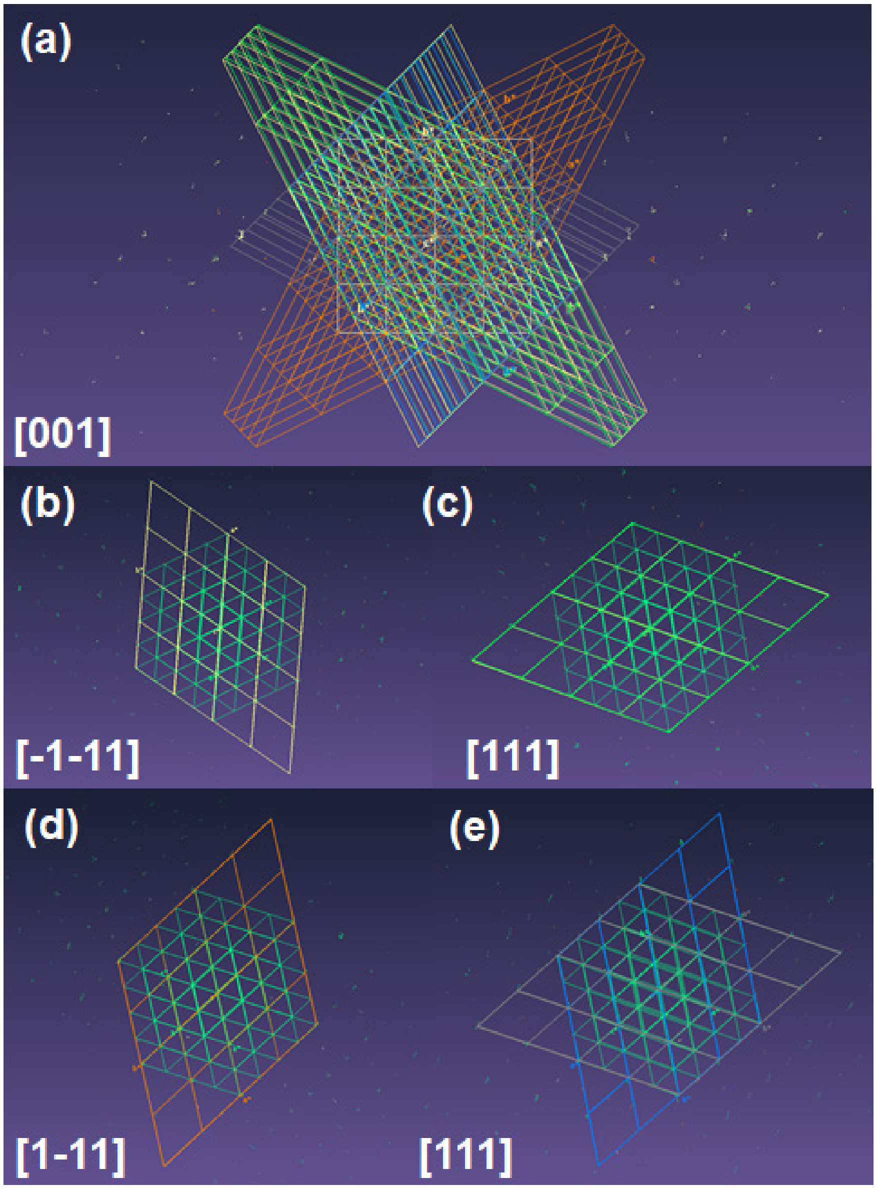

Figure S17. Multi-crystal analysis of CRED data for $LiNi_{0.925}Ti_{0.05625}O_2$ (x = 0.075). (a) 3D distribution of seven $R\bar{3}m$ unit cells compared with a single $Fm\bar{3}m$ cell oriented along the [001] zone axes. (b), c) d) and e) panels show that all the $R\bar{3}m$ unit cells have the *c* axes perpendicular to the family of (111) planes of the cubic unit cells.

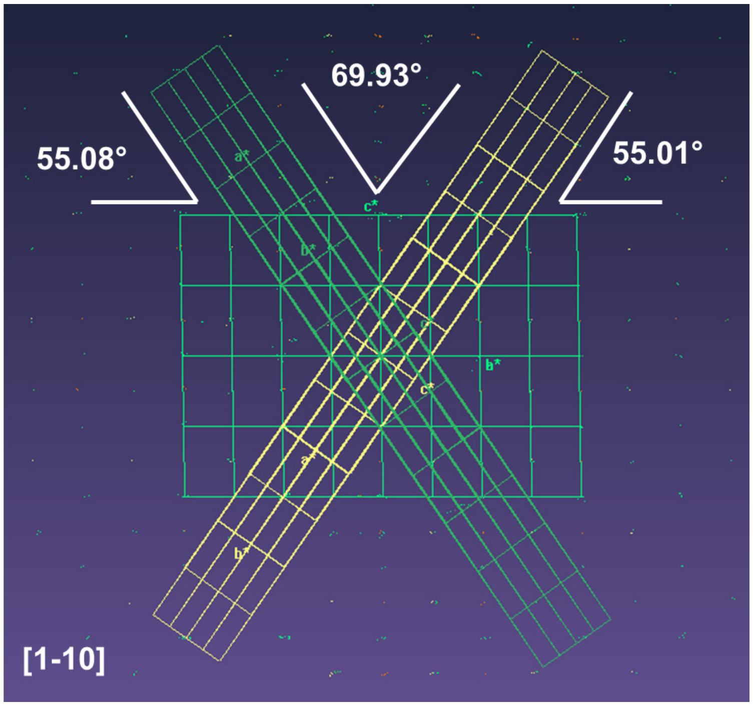

Figure S18. Multi-crystal analysis of CRED data for $LiNi_{0.925}Ti_{0.05625}O_2$ ($x = 0.075$). We observe an $Fm\bar{3}m$ cell oriented in the [1$\bar{1}$0] and two $R\bar{3}m$ unit cells with c axes perpendicular to the [$\bar{1}\bar{1}$1] and [111] separated by angles of ~70° (between $R\bar{3}m$ and $R\bar{3}m$ domains) and ~55° (between $R\bar{3}m$ and $Fm\bar{3}m$ unit cells).

8. Particle size and morphology of $LiNi_{1-x}Ti_{3x/4}O_2$ ($0 \leq x \leq 0.1$)

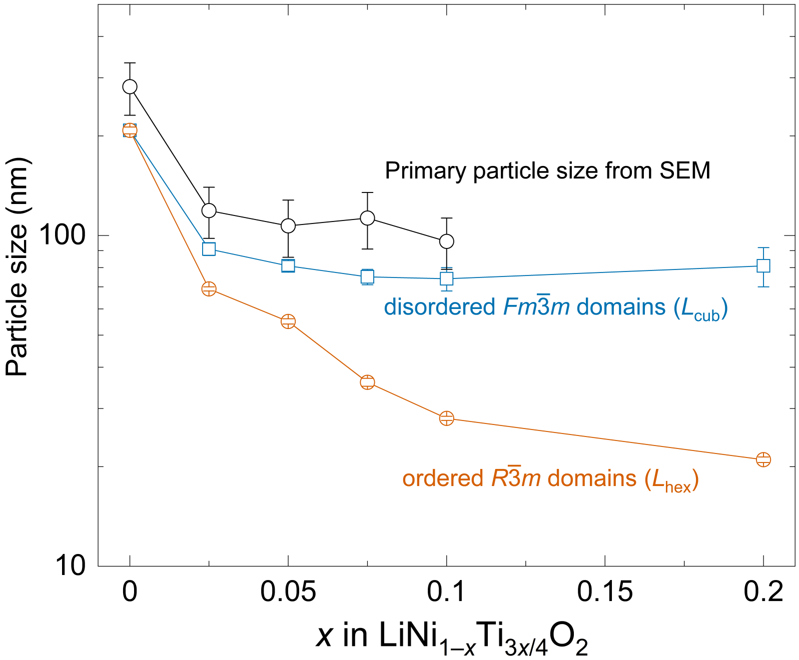

Figure S19. The coherence lengths for the $LiNi_{1-x}Ti_{3x/4}O_2$ ($0 \leq x \leq 0.2$) compositions obtained by refining SPXRD data (λ = 0.825005(1) Å) using the multidomain single-phase hexagonal model described in the main text that simultaneously models ordered ($R\bar{3}m$) and disordered ($Fm\bar{3}m$) rock salt domains (L_{hex} and L_{cub}, respectively). Primary particle sizes of $LiNi_{1-x}Ti_{3x/4}O_2$ ($0 \leq x \leq 0.1$) powders extracted by processing SEM (black circles) images shown in **Figure S20** through ImageJ are also plotted.[18] The instrumental contribution to the PXRD peak broadening was incorporated based on a LaB_6 (NIST SRM 660a) line profile standard.

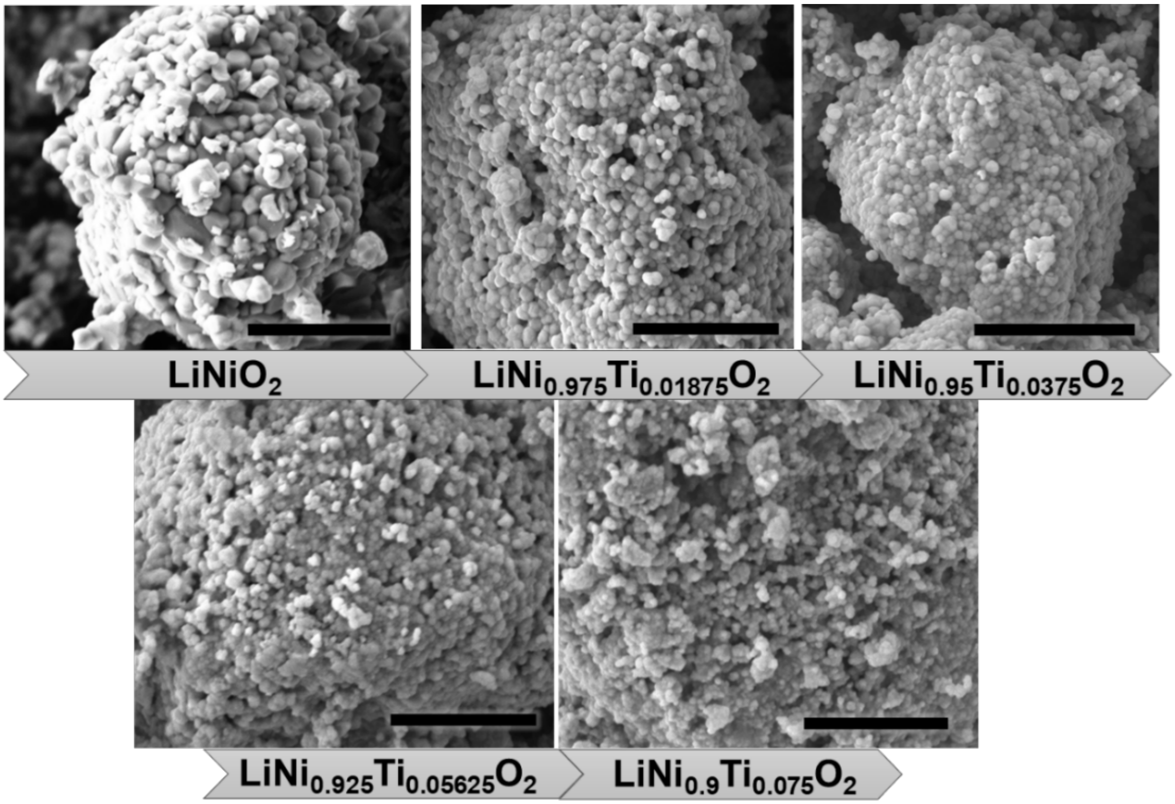

Figure S20. SEM images comparing the primary particles sizes of pristine materials $LiNi_{1-x}Ti_{3x/4}O_2$ ($0 \leq x \leq 0.1$). Black bars shown in each micrograph indicate 2 μm scale.

Table S9. Particle sizes of $LiNi_{1-x}Ti_{3x/4}O_2$ ($0 \leq x \leq 0.1$) extracted by processing of SEM images in Figure S18 through ImageJ,[18] compared against volume-weighted column height crystallite sizes extracted from powder diffraction data for disordered $Fm\overline{3}m$ (L_{cub}) and ordered $R\overline{3}m$ (L_{hex}) domains. The instrumental contribution to the PXRD peak broadening was incorporated based on a LaB_6 (NIST SRM 660a) line profile standard.

x	Composition	Particle size (nm)	L_{cub} (nm)	L_{hex} (nm)
0	$LiNiO_2$	282(51)	208(5)	208(5)
0.025	$LiNi_{0.975}Ti_{0.01875}O_2$	119(21)	91(4)	69(1)
0.05	$LiNi_{0.95}Ti_{0.0375}O_2$	107(21)	81(3)	55(1)
0.075	$LiNi_{0.925}Ti_{0.05625}O_2$	113(22)	75(4)	36(1)
0.1	$LiNi_{0.9}Ti_{0.075}O_2$	96(17)	74(6)	28.0(4)
0.2	$LiNi_{0.8}Ti_{0.15}O_2$	-	81(11)	21.0(4)

9. Comparison of electrochemical performance between $LiNi_{1-x}Ti_{3x/4}O_2$ and stoichiometric $LiNi_{1-x}Ti_xO_2$

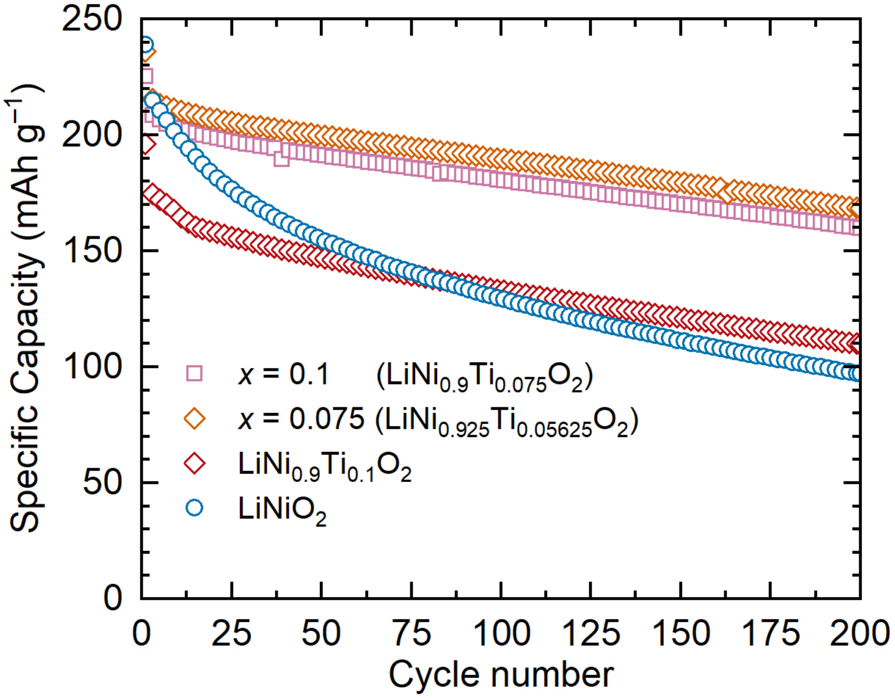

Figure S21. Cycling stability of $LiNi_{1-x}Ti_{3x/4}O_2$ compositions x = 0 ($LiNiO_2$), 0.075 ($LiNi_{0.925}Ti_{0.05625}O_2$) and 0.1 ($LiNi_{0.9}Ti_{0.075}O_2$) compared against that of $LiNi_{0.9}Ti_{0.1}O_2$ from the stoichiometric solid solution $LiNi_{1-x}Ti_xO_2$ with cycle 1 at a current density of 20 mA g^{-1} followed by 100 mA g^{-1} for cycle 2 onwards between 3-4.3 V vs Li^+/Li in 1 M $LiPF_6$ in EC:DMC (1:1, v/v) at 30 °C.

10. Full cell test for $LiNi_{0.925}Ti_{0.05625}O_2$ ($x = 0.075$)

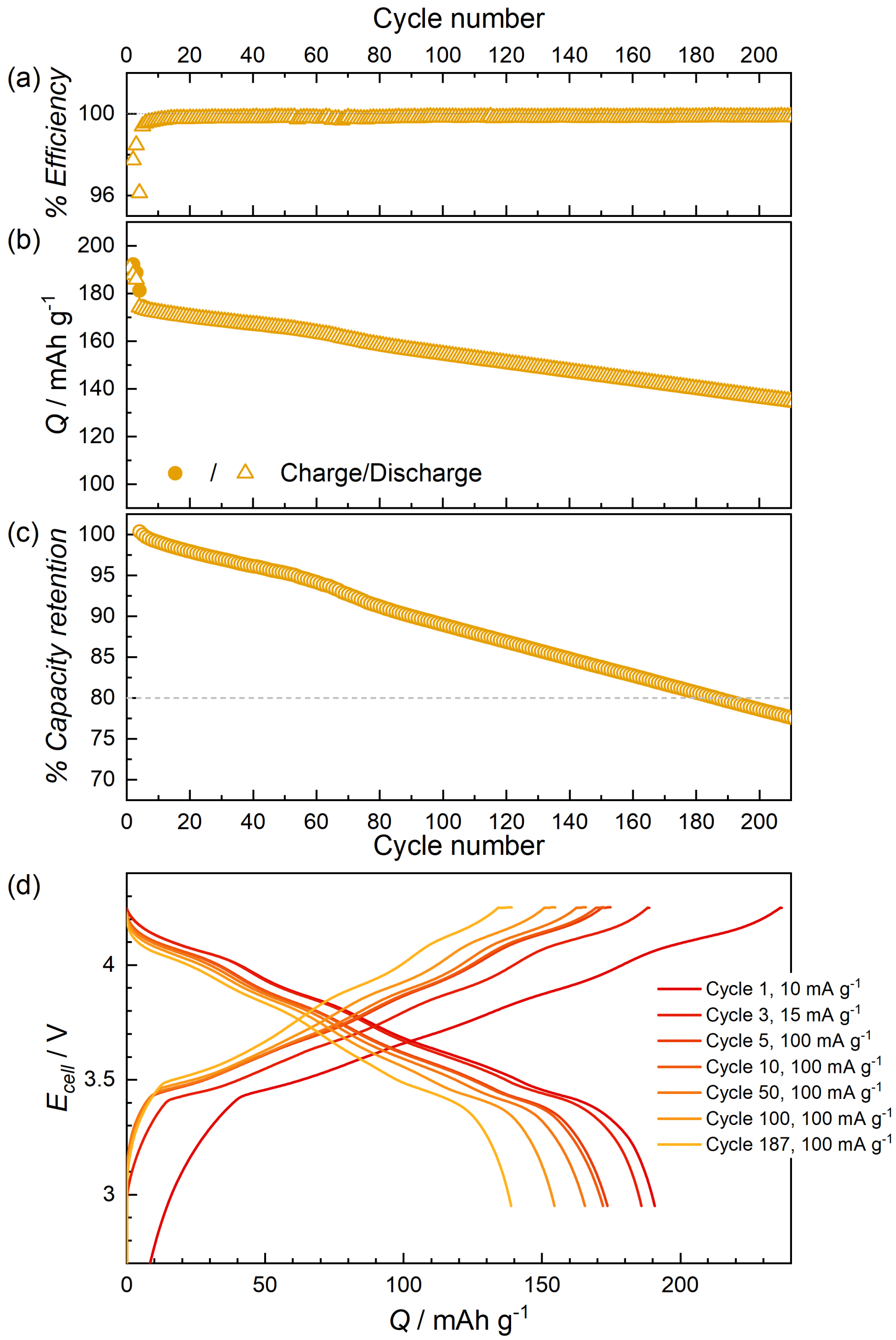

Figure S22. Potential profile and cycling stability of $LiNi_{0.925}Ti_{0.05625}O_2$ with cycle 1 at a current density of 10 mA g^{-1} followed by 2 cycles at 15 mA g^{-1} then 100 mA g^{-1} from cycle 4 onwards between 2.95-4.25 V vs. graphite (SFG44) in 1M $LiPF_6$ with 2 wt% vinylene carbonate in EC:EMC (3:7 vol%).

11. *Ex situ* analysis of cycled $LiNiO_2$ and $LiNi_{0.925}Ti_{0.05625}O_2$ (x = 0.075)

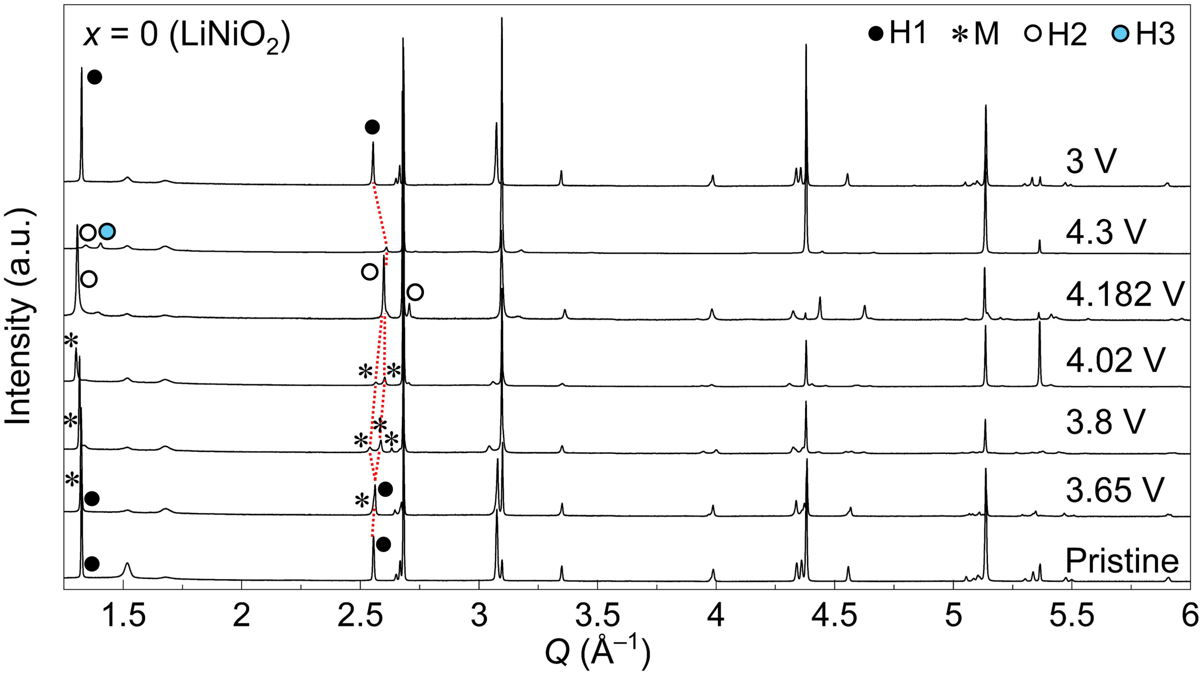

Figure S23. *Ex situ* SPXRD patterns (λ = 0.823899(1) Å) of $LiNiO_2$ at different stages of charge (3.65, 3.8, 4.02, 4.182 and 4.3 V) and full discharge (3 V) in cycle 1 with labels highlighting the structural change of $LiNiO_2$ during charge (red circle for Hexagonal 1 (H1), pink star for monoclinic (M), blue circle for Hexagonal 2 (H2) and green circle for Hexagonal 3 (H3)).

Table S10. Unit cell parameter evolution of $LiNiO_2$ at different stages of charge (3.65, 3.8, 4.02, 4.182 and 4.3 V) and full discharge (3 V) in cycle 1, obtained by Pawley fitting *ex situ* SPXRD patterns (λ = 0.823899(1) Å).

	Phase & unit cell parameters	
Pristine	Hexagonal (a = 2.88209(1) Å, c = 14.21917(11) Å, V = 102.287(1) Å^3	H1
3.65 V	Hexagonal (a = 2.87270(2) Å, c = 14.24410(14) Å, V = 101.800(2) Å^3)<br>Monoclinic (a = 4.9895(3) Å, b = 2.8738(3) Å, c = 5.0242(3) Å, β = 109.322(4)°, V = 67.98(1) Å^3)	H1<br>M
3.8 V	Monoclinic (a = 5.0114(1) Å, b = 2.83414(8) Å, c = 5.0729(1) Å, β = 109.872(2)°, V = 67.761(3) Å^3)	M
4.02 V	Monoclinic (a = 4.9646(2) Å, b = 2.8330(2) Å, c = 5.0888(3) Å, β = 109.415(4)°, V = 67.502(7) Å^3)	M
4.182 V	Hexagonal (a = 2.83029(2) Å, c = 14.4110(2) Å, V = 99.974(2) Å^3)	H2
4.3 V	Hexagonal (a = 2.82483(16) Å, b = 13.4045(11) Å, V = 92.633(13) Å^3)<br>Hexagonal (a = 2.82277(7) Å, c = 14.0466(5) Å, V = 96.928(6) Å^3)	H2 (minor)<br>H3
3 V (dis.)	Hexagonal (a = 2.88309(1) Å, c = 14.21604(8) Å, V = 102.336(1) Å^3)	H1

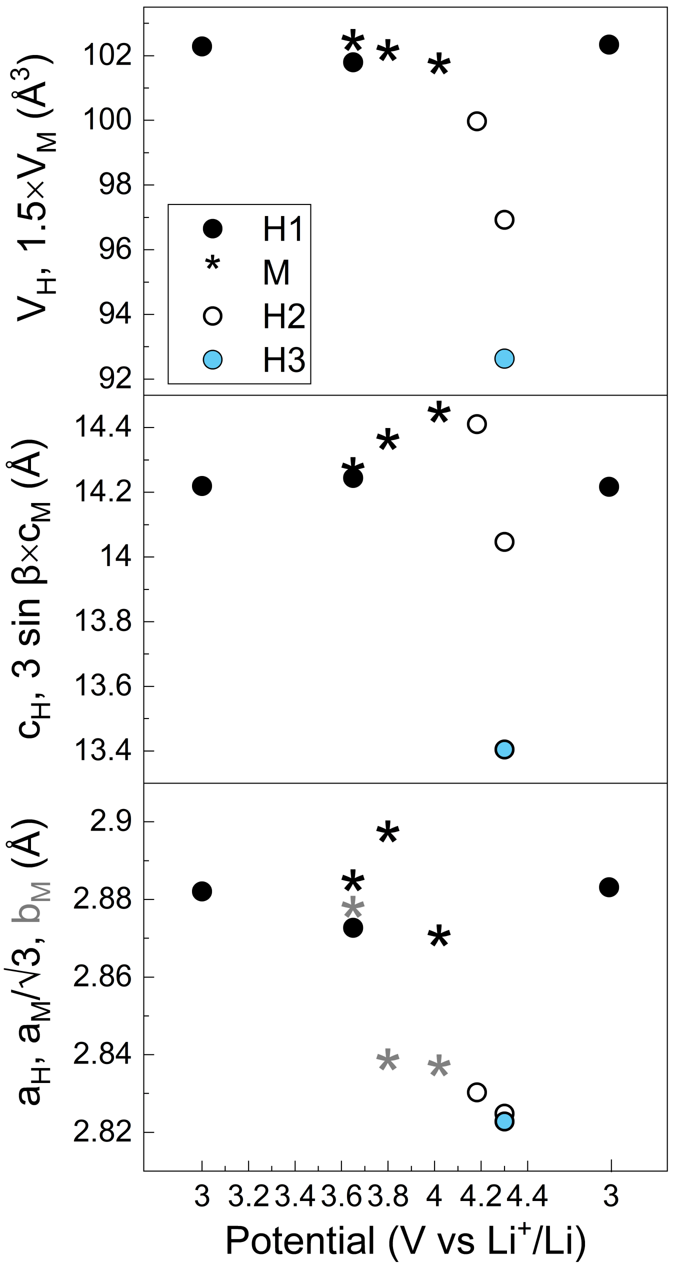

Figure S24. Trend in unit cell parameters refined against SPXRD patterns for *ex situ* samples collected from $LiNiO_2$ at different stages of charge (3.65, 3.8, 4.02, 4.182 and 4.3 V) and full discharge (3 V) in cycle 1. NB: black asterisks in the lower panel correspond to the primitive *a* unit cell parameter of the monoclinic M phase ($a_M/\sqrt{3}$), while grey asterisks correspond to the b_M unit cell parameter of the monoclinic M phase.

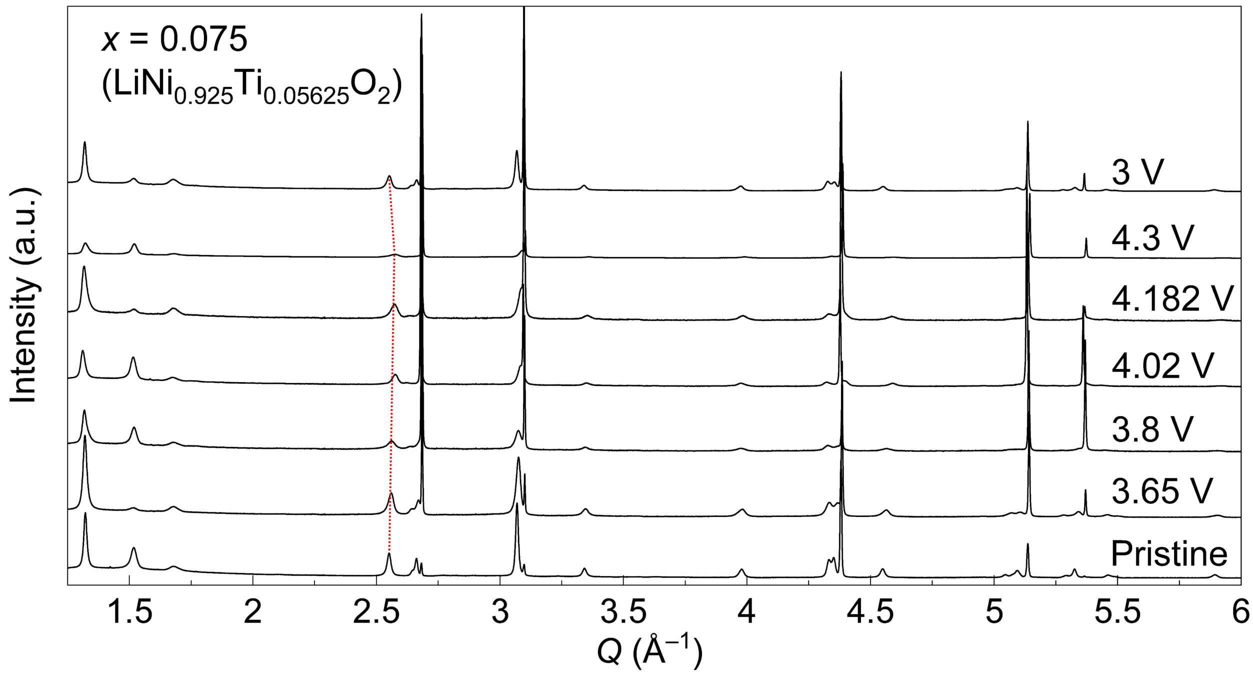

Figure S25. *Ex situ* Synchrotron XRD patterns (λ = 0.823899(1) Å) of $LiNi_{0.925}Ti_{0.05625}O_2$ (x = 0.075) at different stages of charge (3.65, 3.8, 4.02, 4.182 and 4.3 V) and full discharge (3 V) in cycle 1. The $(10\bar{2})_H$ and $(104)_H$ reflections overlap with those of the aluminium current collector.

Table S11. Unit cell parameter evolution obtained by Pawley fitting the *ex situ* SPXRD patterns (λ = 0.823899(1) Å) of $LiNi_{0.925}Ti_{0.05625}O_2$ (x = 0.075) at different stages of charge (3.65, 3.8, 4.02, 4.182 and 4.3 V) and full discharge (3 V) in cycle 1.

State of charge	Phase & unit cell parameters
Pristine	Hexagonal (a = 2.88259(6) Å, c = 14.2233(4) Å, V = 102.35(1) Å^3)
3.65 V	Hexagonal (a = 2.88226(4) Å, c = 14.2902(4) Å, V = 102.81(1) Å^3)
3.8 V	Hexagonal (a = 2.8803(2) Å, c = 14.298(2) Å, V = 102.73(3) Å^3)
4.02 V	Hexagonal (a = 2.8553(4) Å, c = 14.302(4) Å, V = 101.98(5) Å^3)
4.182 V	Hexagonal (a = 2.8655(1) Å, c = 14.289(1), V = 101.61(2) Å^3)
4.3 V (full charge)	Hexagonal (a = 2.8673(1) Å, c = 14.271(1) Å, V = 101.61(1) Å^3)
3 V (discharge)	Hexagonal (a = 2.89101(5) Å, c = 14.2899(6) Å, V = 103.433(6) Å^3)

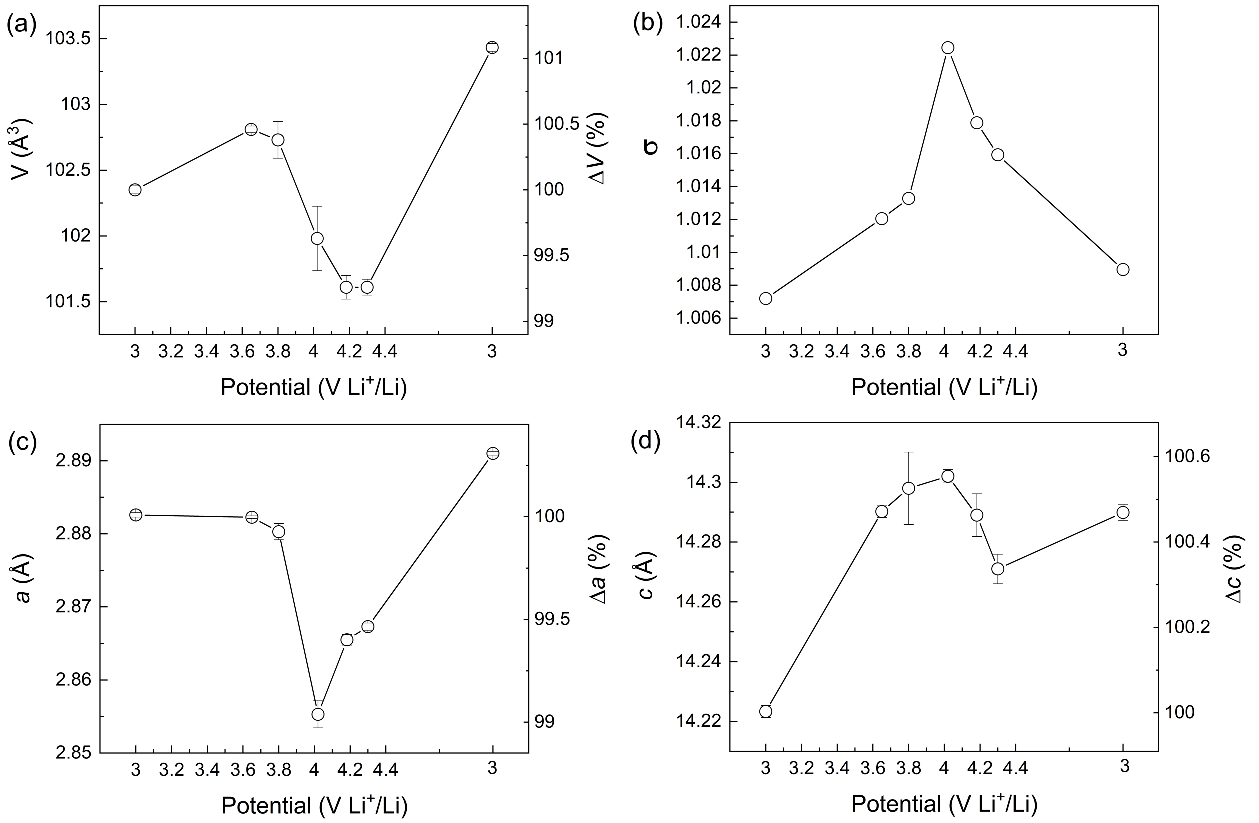

Figure S26. Trend in unit cell **(a)** volume, **(b)** hexagonal distortion parameter (σ), and unit cell **(c)** a and **(d)** c lattice parameters refined against SPXRD patterns using the multidomain single-phase hexagonal model described in the main text from *ex situ* samples collected from $LiNi_{0.925}Ti_{0.05625}O_2$ ($x = 0.075$) at different stages of charge (3.65, 3.8, 4.02, 4.182 and 4.3 V) and full discharge (3 V) in cycle 1. Error bars are multiplied by 5. The hexagonal c lattice parameter is calculated from the hexagonal a lattice parameter and hexagonal distortion parameter through ($c = a\ \sigma\sqrt{24}$).

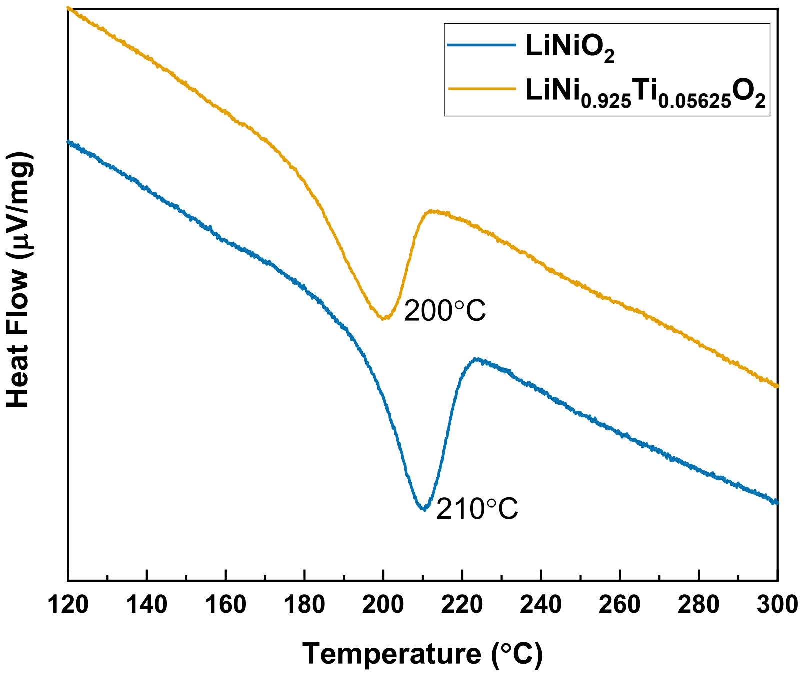

Figure S27. Differential calorimetry (DSC) profiles for fully charged (4.3V) $LiNiO_2$ and $LiNi_{0.925}Ti_{0.05625}O_2$ materials.

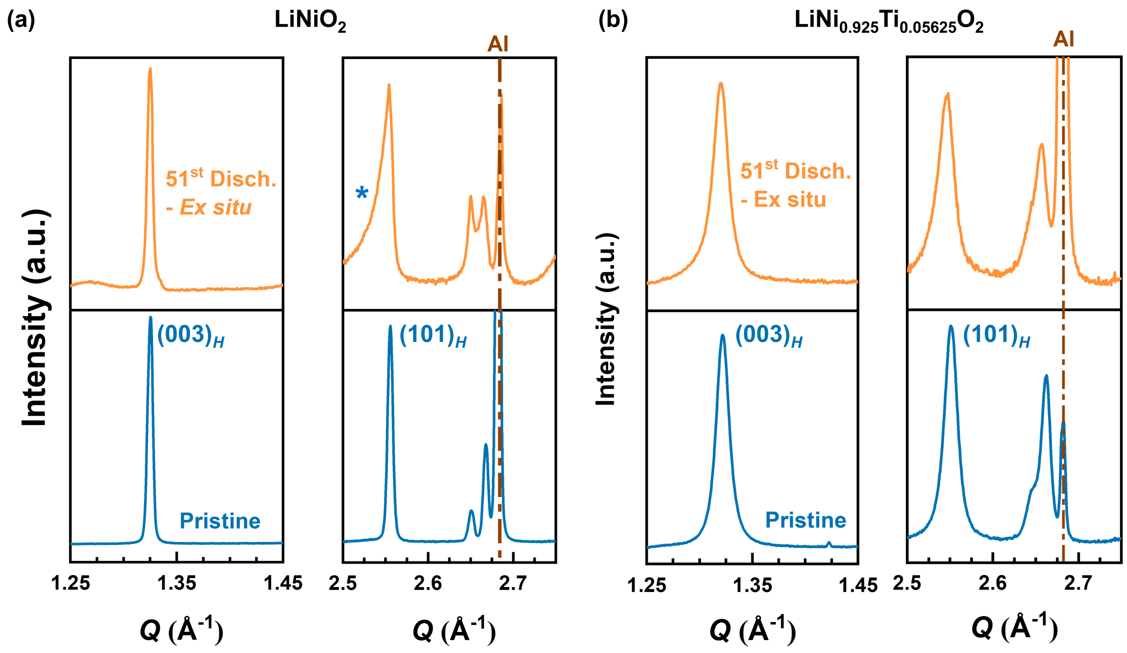

Figure S28. *Ex situ* SPXRD patterns ($\lambda = 0.823899(1)$ Å) of **(a)** $LiNiO_2$ and **(b)** $LiNi_{0.925}Ti_{0.05625}O_2$ ($x = 0.075$) after 50 cycles at a current density of 100 mA g^{-1}. Blue Asterisk in (a) indicates the $(110)_M$ reflection arising from the monoclinic structure.

Table S12. Unit cell parameter evolution obtained by Pawley fitting the *ex situ* SPXRD patterns (λ = 0.823899(1) Å) for $LiNiO_2$ and (b) $LiNi_{0.925}Ti_{0.05625}O_2$ (x = 0.075) after 50 cycles at a current density of 100 mA g^{-1}.

	$LiNiO_2$	$LiNi_{0.925}Ti_{0.05625}O_2$ (x = 0.075)
Pristine	Hexagonal: (a = 2.882094(12) Å, c = 14.21917(11) Å, V = 102.287(1) Å^3)	Hexagonal: (a = 2.88259(6) Å, c = 14.2233(4) Å, V = 102.35(1) Å^3)
After 50 cycles at 100 mA g^{-1}	Hexagonal: (a = 2.8845(1) Å, c = 14.2258(8) Å, V = 102.504(9) Å^3) Monoclinic: (a = 4.9496(8) Å, b = 2.9080(4) Å, c = 5.0216(8) Å, β = 109.093(12)°, V = 68.30(2) Å^3)	Hexagonal: (a = 2.89649(7) Å, c = 14.2818(9) Å, V = 103.77(1) Å^3)

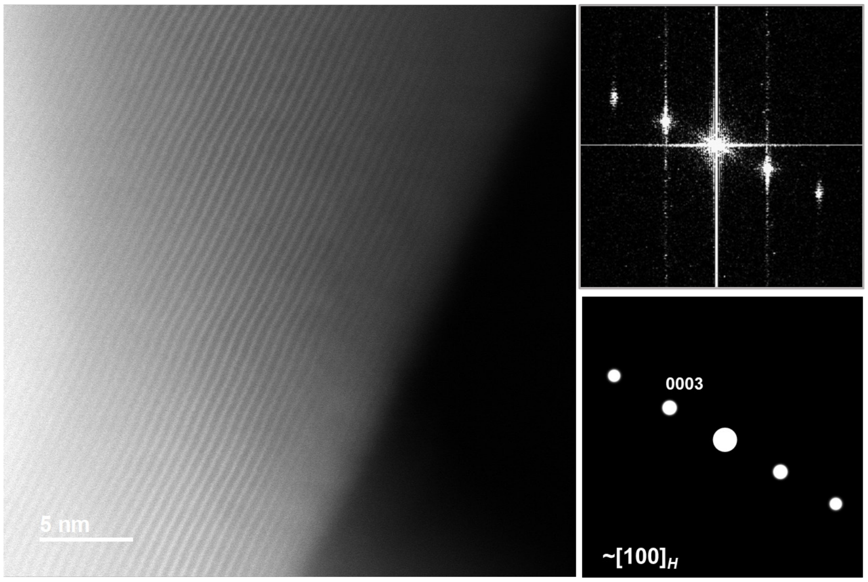

Figure S29. *Ex situ* STEM images for $LiNiO_2$ (x = 0) after 50 cycles at a current density of 100 mA g^{-1} with corresponding FFT (top right) and the simulated FFT pattern (bottom right) of the ordered $R\bar{3}m$ structure oriented along ~[100]$_H$.

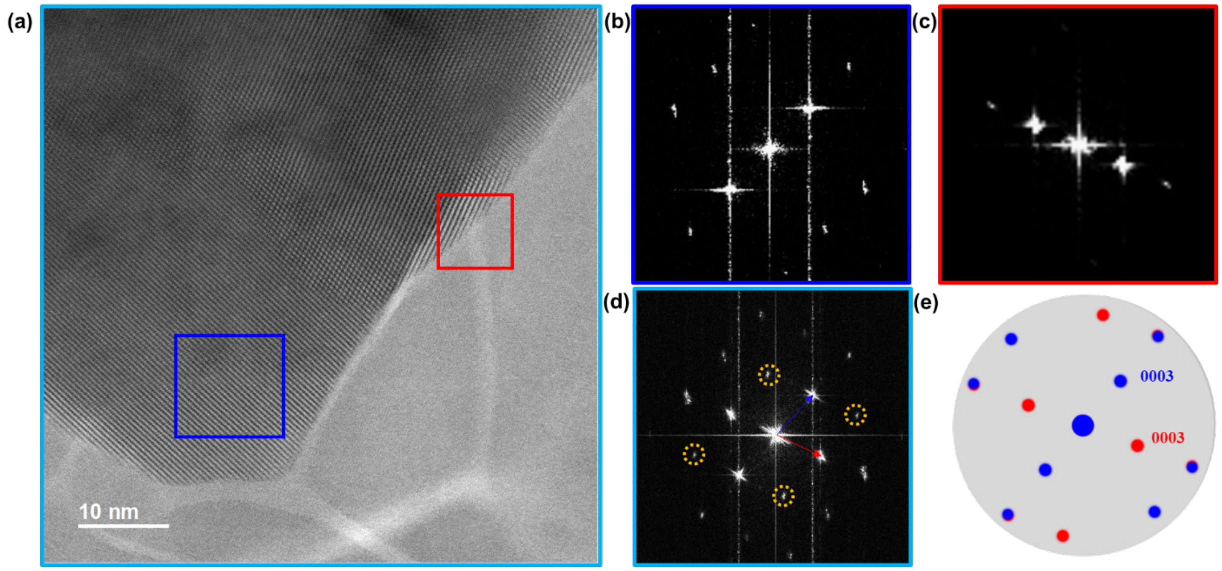

Figure S30. *Ex situ* STEM images for $LiNi_{0.925}Ti_{0.05625}O_2$ ($x = 0.075$) after 50 cycles at a current density of 100 mA g^{-1} showing **(a)** an ordered-ordered $R\bar{3}m$ intergrowth with FFT of the two layered domains highlighted in **(b)** dark blue and **(c)** red in the STEM image. **(d)** The FFT of the entire STEM image (light blue) and **(e)** simulated patterns of two overlapping ordered structures oriented along $[100]_H$ with an angle of ~70°. The contributions of reflections from two ordered domains give rise to weak multiple diffraction (marked with orange circles in (**d**)).

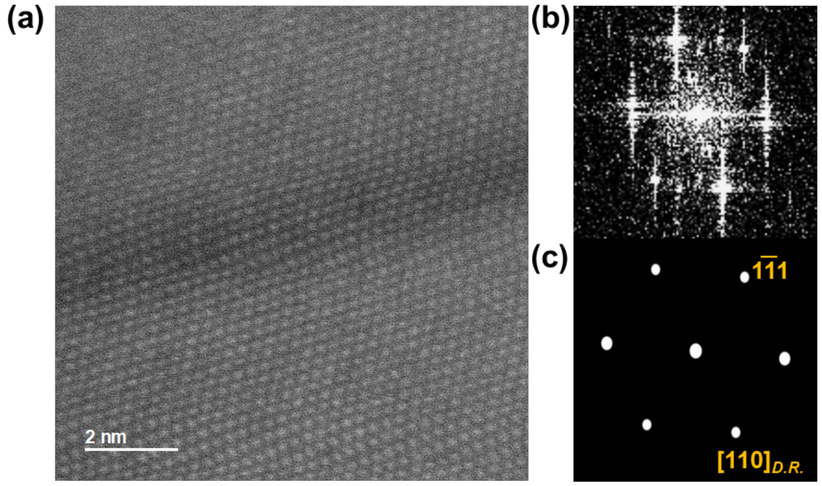

Figure S31. *Ex situ* STEM images for $LiNi_{0.925}Ti_{0.05625}O_2$ ($x = 0.075$) after 50 cycles at a current density of 100 mA g^{-1} showing **(a)** a disordered $Fm\bar{3}m$ rock salt domain with **(b)** corresponding FFT and **(c)** the simulated FFT pattern of disordered $Fm\bar{3}m$ rocksalt domain oriented along $[110]_{D.R.}$.

12. Electrokinetics and structural stability in $LiNi_{1-x}Ti_{3x/4}O_2$

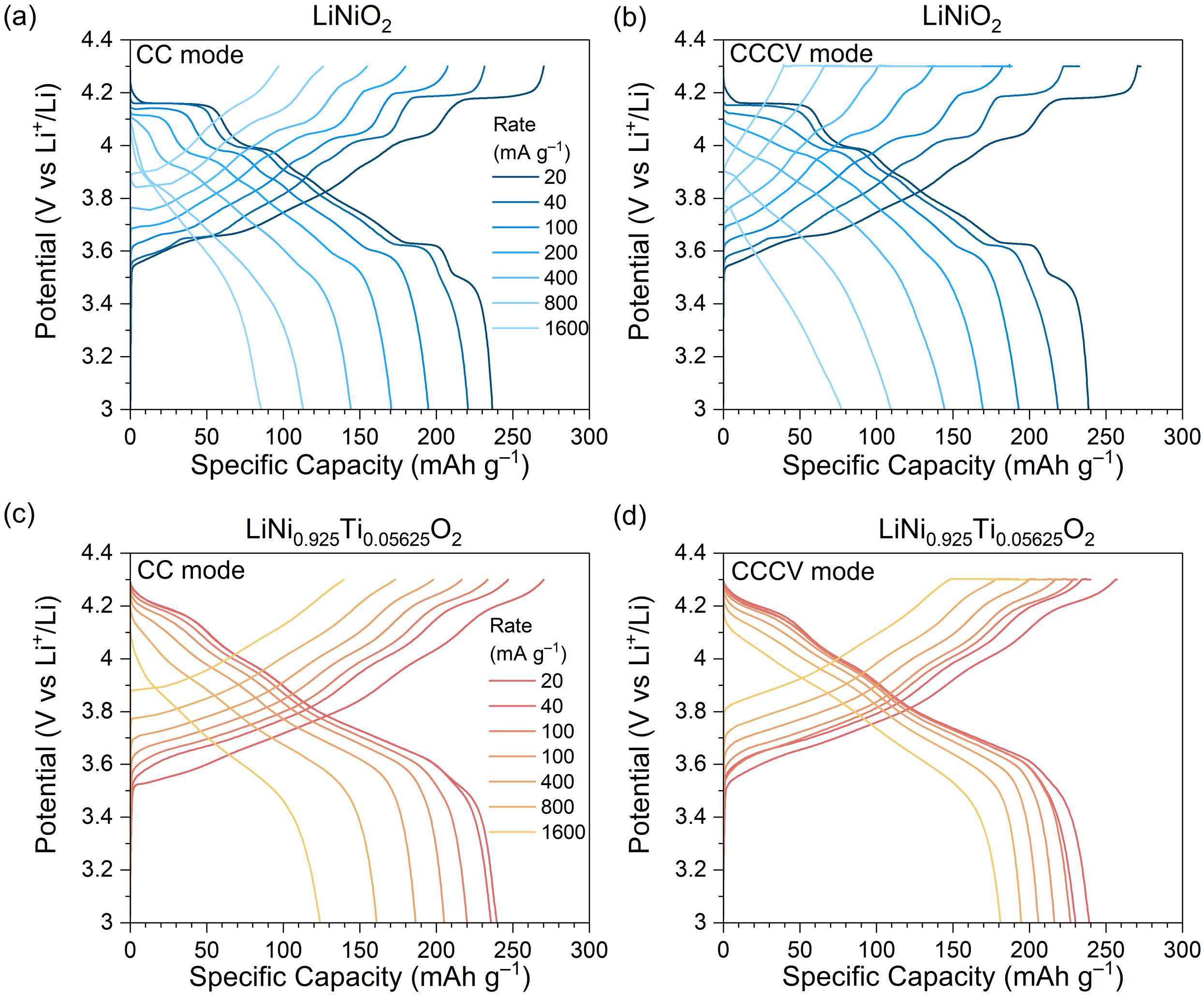

Figure S32. Rate performance of **(a,b)** $LiNiO_2$ and **(c,d)** $LiNi_{0.925}Ti_{0.05625}O_2$ tested between 3 and 4.3 V vs Li^+/Li at current densities between 20 and 1600 mA g^{-1}, under **(a,c)** constant current (CC) mode and **(b,d)** constant current constant voltage (CCCV) mode.

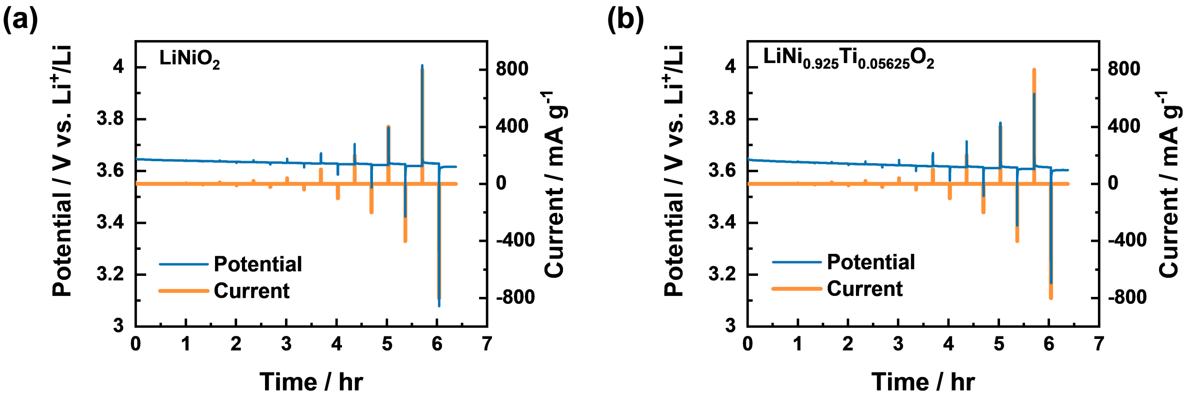

Figure S33. The direct current internal resistance profiles of (a) $LiNiO_2$ and (b) $LiNi_{0.925}Ti_{0.05625}O_2$ at 40% charged state.

Table S13. Recently reported diffusivity values of pristine $LiNiO_2$ and doped-$LiNiO_2$ calculated from galvanostatic intermittent titration technique (GITT) measurements.

**Current density (mA g^{-1})**	**Pulse time (min)**	**Rest time (min)**	**Diffusivity (cm^2 s^{-1})**	**Composition**	**Ref**
20	15	60	10^{-12}-10^{-10}	$LiNiO_2$	This work
			10^{-11}-10^{-10}	$LiNi_{0.925}Ti_{0.05625}O_2$	
22	20	120	10^{-10}-10^{-8}	$LiNiO_2$	[19]
27	10	10	10^{-13}-10^{-11}	$LiNiO_2$	[20]
			10^{-13}-10^{-12}	$LiNi_{0.99}Nb_{0.01}O_2$	
18	15	60	10^{-13}-10^{-10}	$LiNiO_2$	[21]
10	60	600	10^{-11}-10^{-10}	$LiNi_{0.96}Mg_{0.02}Ti_{0.02}O_2$	[22]
18	30	240	10^{-10}-10^{-9}	$LiNiO_2$	[23]
18	10	20	10^{-12}-10^{-9}	$LiNiO_2$	[24]
			10^{-11}-10^{-9}	$LiNi_{1-x}W_xO_2$ (x = 0.01, 0.015 and 0.02)	

13. High current and high loading capability of $x = 0.075$ ($LiNi_{0.925}Ti_{0.05625}O_2$)

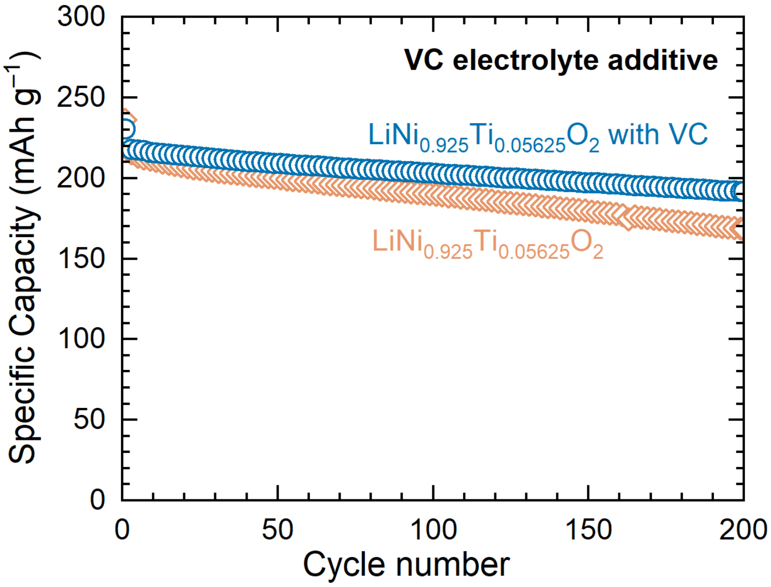

Figure S34. The influence of VC additive in the electrolyte (1M $LiPF_6$, EC: ethyl methyl carbonate (EMC) 3:7, 2wt.% vinylene carbonate (VC)) on the cycling stability of $LiNi_{0.925}Ti_{0.05625}O_2$ tested under same electrochemical conditions (current density of 100 mA g^{-1}).

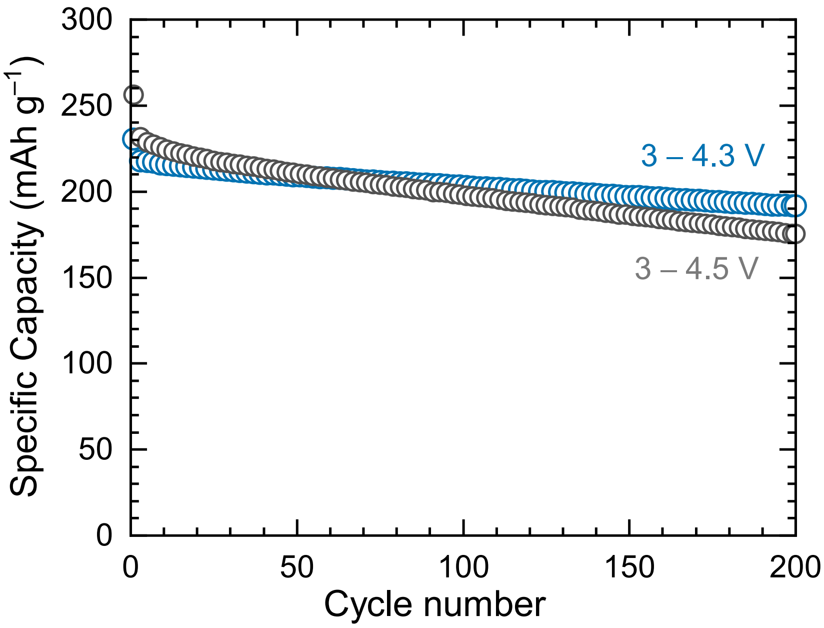

Figure S35. The influence of charge cutoff potential on the cycling stability of $LiNi_{0.925}Ti_{0.05625}O_2$ tested under same electrochemical conditions (current density of 100 mA g^{-1}) using VC additive in the electrolyte (1M $LiPF_6$, EC: ethyl methyl carbonate (EMC) 3:7, 2wt.% vinylene carbonate (VC)).

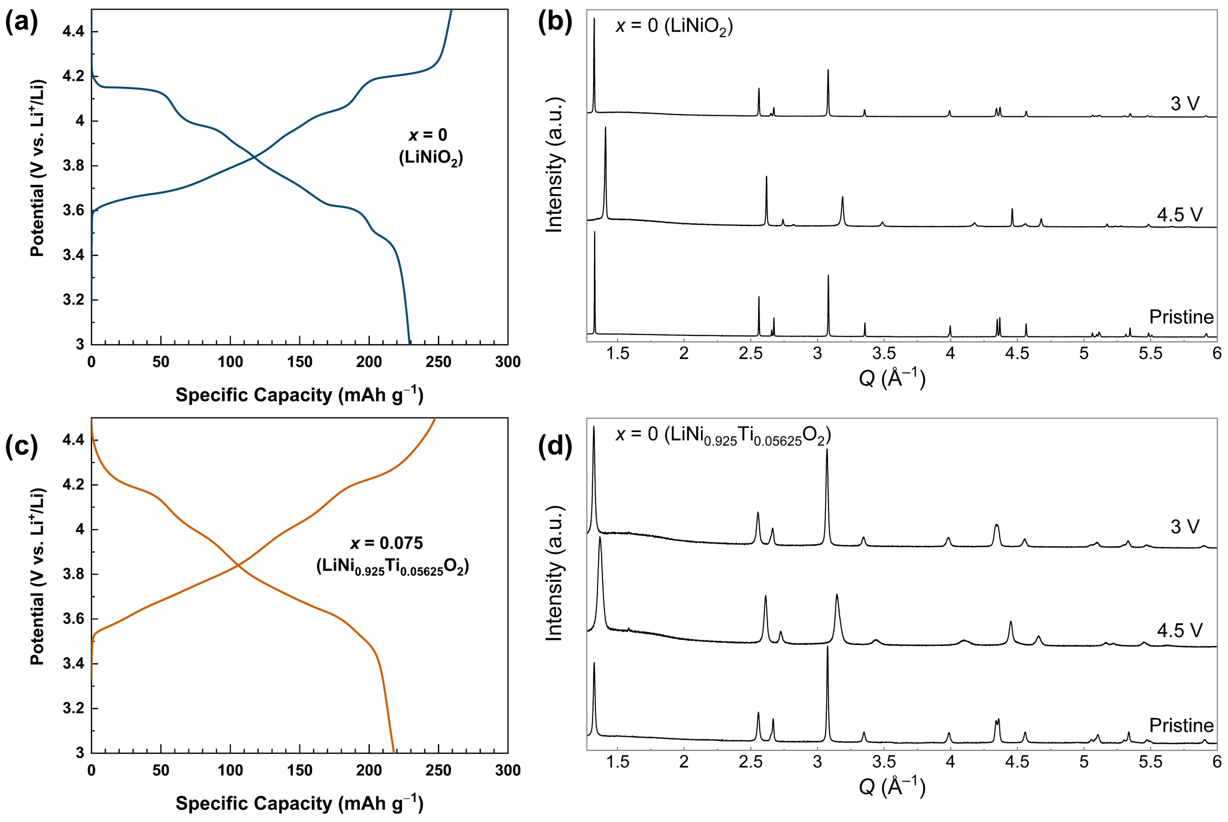

Figure S36. Charge/discharge curves and *ex situ* PXRD patterns ($\lambda = 1.541874$ Å) of **(a, b)** $LiNiO_2$ and **(c, d)** $LiNi_{0.925}Ti_{0.05625}O_2$ at different states of charge (pristine, charged to 4.5V vs. Li^+/Li and discharged 3V vs. Li^+/Li).

Table S14. Unit cell parameter evolution obtained by Pawley fitting *ex situ* PXRD patterns of $LiNiO_2$ (x = 0) and $LiNi_{0.925}Ti_{0.05625}O_2$ ($x = 0.075$) when charged to 4.5 V vs. Li^+/Li and discharged to 3 V vs. Li^+/Li. LaB_6 (NIST SRM 660a) was used as a standard for accurate extraction of unit cell parameters.

	State of charge	*a* (Å)	*c* (Å)	Volume (Å^3)
$LiNiO_2$	Pristine	2.88209(1)	14.21917(11)	102.287(1)
	charged to 4.5 V	2.81723(8)	13.3713(6)	91.907(7)
	discharged to 3 V	2.87622(5)	14.2237(4)	101.903(4)
$LiNi_{0.925}Ti_{0.05625}O_2$	Pristine	2.88259(6)	14.2233(4)	102.35(1)
	charged to 4.5 V	2.8241(2)	13.761(2)	95.04(2)
	discharged to 3 V	2.88497(11)	14.22339(9)	102.598(10)

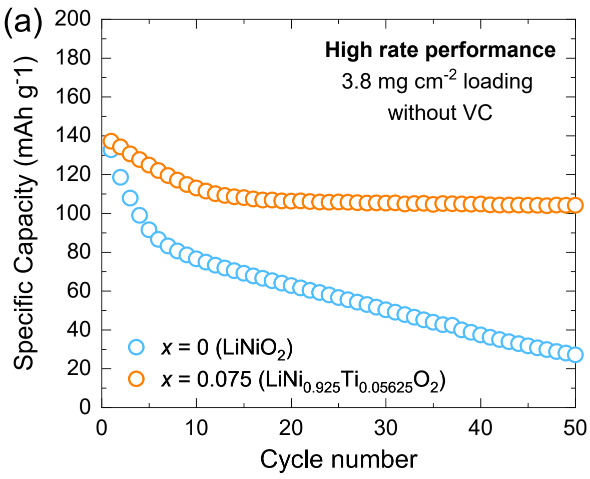

Figure S37. High-rate performance of x = 0 ($LiNiO_2$) (blue circles) and x = 0.075 ($LiNi_{0.925}Ti_{0.05625}O_2$) (orange circles) over 50 cycles at current density of 3200 mA g^{-1} **(a)** without VC and **(b)** with VC electrolyte additive.

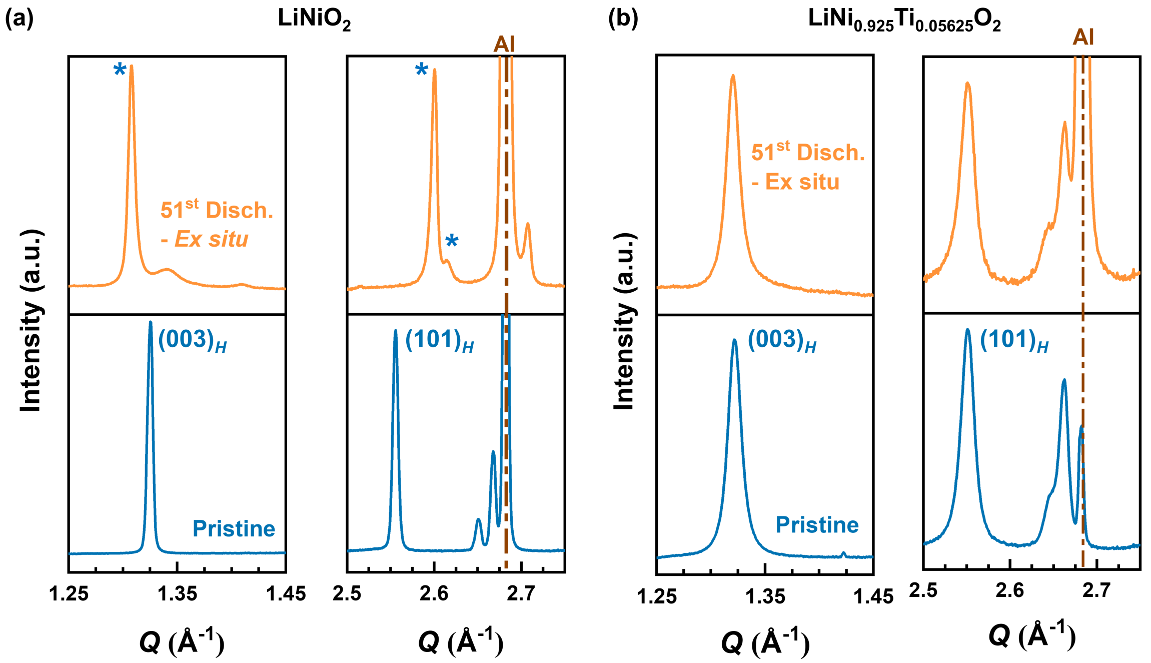

Figure S38. *Ex situ* SPXRD patterns (λ = 0.823899(1) Å) of **(a)** x = 0 ($LiNiO_2$) and **(b)** x = 0.075 ($LiNi_{0.925}Ti_{0.05625}O_2$) after 50 cycles at a current density of 3200 mA g^{-1} with VC electrolyte additive. Blue Asterisk in panel a (top) indicates reflections arising from the monoclinic structure of $LiNiO_2$.

Table S15. Unit cell parameter evolution obtained by Pawley fitting the *ex situ* Synchrotron XRD patterns (λ = 0.823899(1) Å) for $x = 0$ ($LiNiO_2$) and $x = 0.075$ ($LiNi_{0.925}Ti_{0.05625}O_2$) after 50 cycles at a current density of 3200 mA g^{-1}.

	$x = 0$ ($LiNiO_2$)	$x = 0.075$ ($LiNi_{0.925}Ti_{0.05625}O_2$)
Pristine	Hexagonal: (a = 2.882094(12) Å, c = 14.21917(11) Å, V = 102.287(1) Å^3)	Hexagonal: (a = 2.88259(6) Å, c = 14.2233(4) Å, V = 102.35(1) Å^3)
After 50 cycles at 3200 mA g^{-1}	Hexagonal: (a = 2.8381(5) Å, c = 14.0445(11) Å, V = 97.97(3) Å^3) (Hex3-minor) Monoclinic: (a = 4.89299(8) Å, b = 2.83168(11) Å, c = 5.07203(12) Å, β = 108.951(3)°, V = 66.466(3) Å^3)	Hexagonal: (a = 2.89273(12) Å, c = 14.2696(15) Å, V = 103.41(2) Å^3)

References

[1] L. Q. Zhang, H. Noguchi, D. C. Li, T. Muta, X. Q. Wang, M. Yoshio, I. Taniguchi, *J Power Sources* **2008**, 185, 534.
[2] L. Croguennec, E. Suard, P. Willmann, C. Delmas, *Chem. Mater.* **2002**, 14, 2149.
[3] J. Kim, K. Amine, *Electrochem. Commun.* **2001**, 3, 52.
[4] H. Arai, M. Tsuda, Y. Sakurai, *J Power Sources* **2000**, 90, 76.
[5] S. H. Chang, S. G. Kang, S. W. Song, J. B. Yoon, J. H. Choy, *Solid State Ionics* **1996**, 86-8, 171.
[6] L. Q. Zhang, X. Q. Wang, H. Noguchi, M. Yoshio, K. Takada, T. Sasaki, *Electrochim. Acta* **2004**, 49, 3305.
[7] H. W. Ha, K. H. Jeong, K. Kim, *J Power Sources* **2006**, 161, 606.
[8] S. N. Kwon, R. R. Park, M. Y. Song, *Ceram Int* **2014**, 40, 11131.
[9] S. Deng, Y. Li, Q. Dai, J. Fu, Y. Chen, J. Zheng, T. Lei, J. Guo, J. Gao, W. Li, *Sustainable Energy & Fuels* **2019**, 3, 3234.
[10] H. Arai, S. Okada, Y. Sakurai, J. Yamaki, *J. Electrochem. Soc.* **1997**, 144, 3117.
[11] G. T. Park, S. B. Kim, B. Namkoong, N. Y. Park, H. Kim, C. S. Yoon, Y. K. Sun, *Mater. Today* **2023**, 71, 38.
[12] M. Bianchini, M. Roca-Ayats, P. Hartmann, T. Brezesinski, J. Janek, *Angew. Chem., Int. Ed.* **2019**, 58, 10434.
[13] A. Rougier, P. Gravereau, C. Delmas, *J. Electrochem. Soc.* **1996**, 143, 1168.
[14] D. Goonetilleke, B. Schwarz, H. Li, F. Fauth, E. Suard, S. Mangold, S. Indris, T. Brezesinski, M. Bianchini, D. Weber, *J. Mater. Chem. A* **2023**, 11, 13468.
[15] P. T. Barton, Y. D. Premchand, P. A. Chater, R. Seshadri, M. J. Rosseinsky, *Chem. - Eur. J.* **2013**, 19, 14521.
[16] G. Sheldrick, *Acta Crystallogr A* **2015**, 71, 3.
[17] O. V. Dolomanov, L. J. Bourhis, R. J. Gildea, J. A. K. Howard, H. Puschmann, *J. Appl. Cryst.* **2009**, 42, 339.
[18] C. A. Schneider, W. S. Rasband, K. W. Eliceiri, *Nat. Methods* **2012**, 9, 671.
[19] R. A. Yuwono, F. M. Wang, N. L. Wu, Y. C. Chen, H. Chen, J. M. Chen, S. C. Haw, J. F. Lee, R. K. Xie, H. S. Sheu, P. Y. Chang, C. Khotimah, L. Merinda, R. C. Hsing, *Chem. Eng. J.* **2023**, 456, 141065.
[20] G. X. Huang, R. H. Wang, X. Y. Lv, J. Su, Y. F. Long, Z. Z. Qin, Y. X. Wen, *J Electrochem Soc* **2022**, 169, 040533.
[21] L. Su, K. Jarvis, H. Charalambous, A. Dolocan, A. Manthiram, *Adv. Funct. Mater.* **2023**, 33, 2213675.
[22] L. Q. Mu, R. Zhang, W. H. Kan, Y. Zhang, L. X. Li, C. G. Kuai, B. Zydlewski, M. M. Rahman, C. J. Sun, S. Sainio, M. Avdeev, D. Nordlund, H. L. L. Xin, F. Lin, *Chem. Mater.* **2019**, 31, 9769.
[23] H. Li, W. Hua, X. Liu-Théato, Q. Fu, M. Desmau, A. Missyul, M. Knapp, H. Ehrenberg, S. Indris, *Chem. Mater.* **2021**, 33, 9546.
[24] H.-H. Ryu, G.-T. Park, C. S. Yoon, Y.-K. Sun, *J. Mater. Chem. A* **2019**, 7, 18580.
